# Approximation of martingale couplings on the line in the adapted weak topology

**DOI:** 10.1007/s00440-021-01103-y

**Published:** 2022-02-02

**Authors:** M. Beiglböck, B. Jourdain, W. Margheriti, G. Pammer

**Affiliations:** 1 University of Vienna, Vienna, Austria; 2 CERMICS, Ecole des Ponts, INRIA, Marne-la-Vallée, Paris, France; 3 ETH Zürich, Zürich, Switzerland

**Keywords:** Martingale optimal transport, Adapted Wasserstein distance, Stability

## Abstract

Our main result is to establish stability of martingale couplings: suppose that $\pi$ is a martingale coupling with marginals $\mu , \nu$. Then, given approximating marginal measures $\tilde{\mu }\approx \mu , \tilde{\nu }\approx \nu$ in convex order, we show that there exists an approximating martingale coupling $\tilde{\pi }\approx \pi$ with marginals $\tilde{\mu }, \tilde{\nu }$. In mathematical finance, prices of European call/put option yield information on the marginal measures of the arbitrage free pricing measures. The above result asserts that small variations of call/put prices lead only to small variations on the level of arbitrage free pricing measures. While these facts have been anticipated for some time, the actual proof requires somewhat intricate stability results for the adapted Wasserstein distance. Notably the result has consequences for several related problems. Specifically, it is relevant for numerical approximations, it leads to a new proof of the monotonicity principle of martingale optimal transport and it implies stability of weak martingale optimal transport as well as optimal Skorokhod embedding. On the mathematical finance side this yields continuity of the robust pricing problem for exotic options and VIX options with respect to market data. These applications will be detailed in two companion papers.

## Introduction

Before carefully explaining all required notation and describing relevant literature, let us give a first description of our main result and its relevance for the martingale transport theory.

While classical transport theory is concerned with the set $\Pi (\mu , \nu )$ of *couplings* or *transport plans* of probability measures $\mu ,\nu$, the martingale variant restricts the problem to the set $\Pi _M(\mu , \nu )$ of *martingale couplings*, that is, transport plans which preserve the barycenter of each particle. Even though the main interest lies in the case where $\mu , \nu$ are probabilities on the real line, many of the basic arguments and results appear significantly more involved in the martingale context. A basic explanation lies in the rigidity of the martingale condition that makes classically simple approximation results quite intricate. Specifically, the martingale theory has been missing a counterpart to the following straightforward fact of the classical transport theory:

### Fact 1.1

(Stability of couplings) Let $\pi \in \Pi (\mu , \nu )$ and assume that $\mu ^k,\nu ^k$, $k\in \mathbb {N}$, are probabilities that converge weakly to $\mu$ and $\nu$. Then there exist couplings $\pi ^k\in \Pi (\mu ^k,\nu ^k), k\in \mathbb {N}$ converging weakly to $\pi$.

This result is so basic and straightforward that its implicit use is easily overlooked. Note however that it plays a crucial role in a number of occasions, e.g. for stability of optimal transport, providing numerical approximations, or in the characterisation of optimality through cyclical monotonicity.

The main result of this article is to establish Fact 1.1 for martingale transports on the real line, see Theorem 2.6 below. This closes a gap in the theory of martingale transport and yields basic fundamental results in a unified fashion that is much closer to the classical theory. It allows to address questions in martingale optimal transport, optimal Skorokhod embedding and robust finance that have previously remained open. These applications are considered systematically in two accompanying articles, see [12] for the first of the two. Among other results, we establish therein the stability of the superreplication bound for VIX futures as well as the stability of the stretched Brownian motion. Moreover, we derive sufficiency of a monotonicity principle, in the spirit of cyclical monotonicity of classical optimal transport, for the weak martingale optimal transport problem and are able to generalize the results concerning the corresponding notion of monotonicity in martingale optimal transport.

We note that while virtually all (to the best of our knowledge) applications of martingale optimal transport are concerned with the case where $\mu , \nu$ are supported on $\mathbb {R}$, it is a highly intriguing challenge to extend the martingale transport theory to the case where $\mu , \nu$ are supported on $\mathbb {R}^d, d> 1$. In a remarkable contrast to our main result, stability of martingale optimal transport breaks down in higher dimensions as has been recently established by Brückerhoff and Juillet [17].

### The martingale optimal transport problem

Let $(X,d_X)$, $(Y,d_Y)$ be Polish spaces and $C:X\times Y\rightarrow \mathbb {R}_+$ be a nonnegative measurable function. Denote by ${\mathcal {P}}(X)$ the set of probability measures on *X*. For $\mu \in {\mathcal {P}}(X)$ and $\nu \in {\mathcal {P}}(Y)$, the classical Optimal Transport problem consists in minimising

OT $$\begin{aligned} \inf _{\pi \in \Pi (\mu ,\nu )}\int _{X\times Y}C(x,y)\,\pi (dx,dy), \end{aligned}$$

where $\Pi (\mu ,\nu )$ denotes the set of probability measures in ${\mathcal {P}}(X\times Y)$ with the first marginal $\mu$ and the second marginal $\nu$. When $X=Y$ and $C=d_X^r$ for some $r\ge 1$, (OT) corresponds to the well-known Wasserstein distance with index *r* to the power *r*, denoted ${\mathcal {W}}_r^r(\mu ,\nu )$, see [4, 51, 53, 54] for a study in depth.

The theory of OT goes back to Monge [44] in its original formulation and Kantorovich [39] in its modern formulation. It was rediscovered many times under various forms and has an impressive scope of applications. A variant of OT that is motivated by applications in mathematical finance, in particular in model-independent pricing, was introduced in [11] in a discrete time setting and in [26] in a continuous time setting. Compared to the usual OT, the difference is that one requires an additional martingale constraint to (OT) which reflects the condition for a financial market to be free of arbitrage.

In detail, the Martingale Optimal Transport (MOT) problem is formulated as follows: given $\pi \in {\mathcal {P}}(\mathbb {R}\times \mathbb {R})$, we denote by $(\pi _x)_{x\in X}$ a regular conditional disintegration with respect to its first marginal $\mu$. We then write $\pi (dx,dy)=\mu (dx)\,\pi _x(dy)$, or with a slight abuse of notation, $\pi =\mu \times \pi _x$ if the context is not ambiguous. Let $C:\mathbb {R}\times \mathbb {R}\rightarrow \mathbb {R}_+$ be a nonnegative measurable function and $\mu$, $\nu$ be two probability distributions on the real line with finite first moment. Then the MOT problem consists in minimising

MOT $$\begin{aligned} \inf _{\pi \in \Pi _M(\mu ,\nu )}\int _{\mathbb {R}\times \mathbb {R}}C(x,y)\,\pi (dx,dy), \end{aligned}$$

where $\Pi _M(\mu ,\nu )$ denotes the set of martingale couplings between $\mu$ and $\nu$, that is

$$\begin{aligned} \Pi _M(\mu ,\nu )=\left\{ \pi =\mu \times \pi _x\in \Pi (\mu ,\nu )\mid \mu (dx)\text {-almost everywhere},\ \int _\mathbb {R}y\,\pi _x(dy)=x\right\} . \end{aligned}$$

According to Strassen’s theorem [52], the existence of a martingale coupling between two probability measures $\mu ,\nu \in {\mathcal {P}}(\mathbb {R})$ with finite first moment is equivalent to $\mu \le _c\nu$, where $\le _c$ denotes the convex order. We recall that two finite positive measures $\mu ,\nu$ on $\mathbb {R}$ with finite first moment and are said to be in the convex order if and only if we have

$$\begin{aligned} \int _{\mathbb {R}}f(x)\,\mu (dx)\le \int _{\mathbb {R}}f(y)\,\nu (dy), \end{aligned}$$

for every convex function $f:\mathbb {R}\rightarrow (-\infty , \infty ]$. Note that there holds equality for all affine functions, from which we deduce that $\mu$ and $\nu$ have equal masses and satisfy $\int _{\mathbb {R}} x\,\mu (dx)=\int _{\mathbb {R}} y\,\nu (dy)$.

For adaptations of classical optimal transport theory to the MOT problem, we refer to [33, 34, 36]. Concerning duality results, we refer to [15, 18, 21, 23]. We also refer to [20, 22, 27, 46] for the multi-dimensional case and to [10, 14] for connections to Skorokhod embedding problem.

Concerning the numerical resolution of the MOT problem, we refer to the articles [2, 3, 19, 30, 32]. When $\mu$ and $\nu$ are finitely supported, then the MOT problem amounts to linear programming. In the general case, once the MOT problem is discretised by approximating $\mu$ and $\nu$ by probability measures with finite support and in the convex order, Alfonsi, Corbetta and Jourdain [3] raised the question of the convergence of optimal costs of the discretised problem towards the costs of the original problem. A first partial result was obtained by Juillet [38] who established stability of left-curtain coupling. Guo and Obłój [30] establish the result under moment conditions. More recently, [9, 55] independently gave a definite positive answer.

### The adapted Wasserstein distance

The stability result shown in [9] involves Wasserstein convergence. More precisely, let $\mu ^k,\nu ^k\in {\mathcal {P}}(\mathbb {R})$, $k\in \mathbb {N}$ be in the convex order and respectively converge to $\mu$ and $\nu$ in ${\mathcal {W}}_r$. Under mild assumption, for all $k\in \mathbb {N}$ there exists $\pi ^k\in \Pi _M(\mu ^k,\nu ^k)$, optimal for (MOT), and any accumulation point of $(\pi ^k)_{k\in \mathbb {N}}$ with respect to the ${\mathcal {W}}_r$-convergence is a martingale coupling between $\mu$ and $\nu$ optimal for (MOT).

**Fig. 1:**
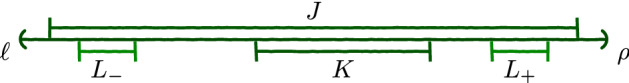
Intervals involved in the proof. The boundaries of the closed intervals are vertical bars and those of the open intervals are parentheses

However, it turns out that the usual weak topology / Wasserstein distance is not well suited in setups where accumulation of information plays a distinct role, e.g. in mathematical finance. Indeed, the symmetry of this distance does not take into account the temporal structure of stochastic processes. It is easy to convince oneself that two stochastic processes very close in Wasserstein distance can yield radically unalike information, as illustrated in [5, Figure 1]. Therefore, one needs to strengthen, the usual topology of weak convergence accordingly. Over time numerous researchers have independently introduced refinements of the weak topology, we mention Hellwig’s information topology [31], Aldous’s extended weak topology [1], the nested distance / adapted Wasserstein distance of Plug-Pichler [47] and the optimal stopping topology [6]. Strikingly, all those seemingly different definitions lead to same topology in the present discrete time [6, Theorem 1.1] framework. We refer to this topology as the *adapted weak topology*. A natural compatible metric is given by the *adapted Wasserstein distance*, see [16, 43, 47–50] among others.

Fix $x_0\in X$ and $r\ge 1$. We denote the set of all probability measures on *X* with finite *r*-th moment by ${\mathcal {P}}_r(X)$, i.e.

$$\begin{aligned} {\mathcal {P}}_r(X)=\left\{ p\in \mathcal P(X)\mid \int _Xd_X^r(x,x_0)\,p(dx)<+\infty \right\} . \end{aligned}$$

Let ${\mathcal {M}}(X)$ (resp. ${\mathcal {M}}_r(X)$) denote the set of all finite positive measures (resp. with finite *r*-th moment). The sets ${\mathcal {M}}(X)$ and ${\mathcal {M}}_r(X)$, resp. are equipped with the weak topology induced by the set $C_b(X)$ of all real-valued absolutely bounded continuous functions on *X* and, resp., the set $\Phi _r(X)$ of all real-valued continuous functions on *X*, *C*(*X*), which satisfy the growth constraint

$$\begin{aligned} \Phi _r(X)=\left\{ f\in C(X)\mid \exists \alpha >0,\ \forall x\in X,\ \vert f(x)\vert \le \alpha \left( 1+d_X^r(x,x_0)\right) \right\} . \end{aligned}$$

A sequence $(\mu ^k)_{k\in \mathbb {N}}$ converges in ${\mathcal {M}}_r(X)$ to $\mu$ if and only if

1.1 $$\begin{aligned} \forall f\in \Phi _r(X),\quad \mu ^k(f) \underset{k\rightarrow +\infty }{\longrightarrow } \mu (f). \end{aligned}$$

If moreover $\mu$ and $\mu ^k$, $k\in \mathbb {N}$, have equal masses, then the convergence (1.1) can be equivalently formulated (see for instance [54, Theorem 6.9]) in terms of the Wasserstein distance with index *r*:

$$\begin{aligned} {\mathcal {W}}_r(\mu ^k,\mu ) :=\inf _{\pi \in \Pi (\mu ^k,\mu )} \left( \int _{X\times X} d_X^r(x,y)\,\pi (dx,dy)\right) ^{\frac{1}{r}}\underset{k\rightarrow +\infty }{\longrightarrow }0. \end{aligned}$$

Given $m_0>0$, we can then equip the set of finite positive measures in ${\mathcal {M}}_r(X\times Y)$ with mass $m_0$ with the Wasserstein topology. However, we can also equip it with a stronger topology, namely the adapted Wasserstein topology. It is induced by the metric ${\mathcal {A}}{\mathcal {W}}_r$ defined for all $\pi ,\pi '\in \mathcal M_r(X\times Y)$ such that $\pi (X\times Y)=\pi '(X\times Y)=m_0$ by

1.2 $$\begin{aligned} {\mathcal {A}}{\mathcal {W}}_r(\pi ,\pi ') =\inf _{\chi \in \Pi (\mu ,\mu ')} \left( \int _{X\times X} \left( d_X^r(x,x') + \mathcal W_r^r(\pi _{x},\pi '_{x'})\right) \, \chi (dx,dx')\right) ^{\frac{1}{r}}, \end{aligned}$$

where $\mu$, resp. $\mu '$, is the first marginal of $\pi$, resp. $\pi '$. It is easy to check that ${\mathcal {W}}_r \le {\mathcal {A}}{\mathcal {W}}_r$, and therefore ${\mathcal {A}}{\mathcal {W}}_r$ indeed induces a stronger topology than ${\mathcal {W}}_r$. Another useful point of view is the following: let $J:{\mathcal {M}}(X\times Y)\rightarrow \mathcal M(X\times {\mathcal {P}}(Y))$ be the inclusion map defined for all $\pi =\mu \times \pi _x\in {\mathcal {M}}(X\times Y)$ by

$$\begin{aligned} J(\pi )(dx,dp)=\mu (dx)\,\delta _{\pi _x}(dp). \end{aligned}$$

For all $\pi ,\pi '\in {\mathcal {M}}_r(X\times Y)$ with equal masses, their adapted Wasserstein distance coincides with

1.3 $$\begin{aligned} {\mathcal {A}}{\mathcal {W}}_r(\pi ,\pi ') = \mathcal W_r(J(\pi ),J(\pi ')). \end{aligned}$$

It follows that the topology induced by ${\mathcal {A}}{\mathcal {W}}_r$ coincides with the weak topology induced by *J*.

Finally, let us mention the interpretation of the adapted Wasserstein distance in terms of bicausal couplings (cf. [7]). Let $\pi ,\pi '\in {\mathcal {P}}_r(X\times Y)$. Let $Z_1,Z_2,Z'_1,Z'_2$ be random variables such that the distribution of $(Z_1,Z_2,Z'_1,Z'_2)$ is a ${\mathcal {W}}_r$-optimal coupling between $\pi$ and $\pi '$. In many cases, there exists a Monge transport map $T:X\times Y\rightarrow X\times Y$ such that $(Z'_1,Z'_2)=T(Z_1,Z_2)$. As mentioned in [5], the temporal structure of stochastic processes is then not taken into account since the present value $Z'_1$ is determined from the future value $Z_2$. Therefore, it is more suitable to restrict to couplings $(Z_1,Z_2,Z'_1,Z'_2)$ between $\pi$ and $\pi '$ such that the conditional distribution of $Z'_1$ (resp. $Z_1$) given $(Z_1,Z_2)$ (resp. $(Z'_1,Z'_2)$) is equal to the conditional distribution of $Z'_1$ (resp. $Z_1$) given $Z_1$ (resp. $Z'_1$).

Let $\mu$ and $\mu '$ denote the respective first marginal distributions of $\pi$ and $\pi '$ and let $\eta \in \Pi (\pi ,\pi ')$ be a coupling between $\pi$ and $\pi '$. Let $\chi (dx,dx')=\int _{(y,y')\in Y\times Y}\eta (dx,dy,dx',dy')\in \Pi (\mu ,\mu ')$. W e write $\chi (dx,dx')=\mu (dx)\,\chi _x(dx')=\mu '(dx')\,\overleftarrow{\chi }_{x'}(dx)$. Then $\eta$ is called bicausal if and only if

$$\begin{aligned}&\int _{y'\in Y}\eta (dx,dy,dx',dy')=\pi (dx,dy)\,\chi _x(dx')\\&\quad \text {and}\quad \int _{y\in Y}\eta (dx,dy,dx',dy')=\pi '(dx',dy')\,\overleftarrow{\chi }_{x'}(dx). \end{aligned}$$

We denote by $\Pi _{bc}(\pi ,\pi ')$ the set of bicausal couplings between $\pi$ and $\pi '$. Let $(\gamma _{(x,x')}(dy,dy'))_{(x,x')\in X\times X}$ be a probability kernel such that $\eta (dx,dy,dx',dy')=\chi (dx,dx')\,\gamma _{(x,x')}(dy,dy')$. Another useful characterisation is that $\eta$ is bicausal if and only if $\chi (dx,dx')\text {-almost everywhere}$, $\gamma _{(x,x')}(dy,dy')\in \Pi (\pi _x,\pi _{x'})$. Then the adapted Wasserstein distance coincides with

$$\begin{aligned} {\mathcal {A}}{\mathcal {W}}_r(\pi ,\pi ')=\inf _{\eta \in \Pi _{bc}(\pi ,\pi ')} \left( \int _{X\times Y}\left( d_X^r(x,x')+d_Y^r(y,y')\right) \,\eta (dx,dy,dx',dy')\right) ^{\frac{1}{r}}. \end{aligned}$$

One of the objectives of the present paper is to prove that well-known stability results for the ${\mathcal {W}}_r$-convergence also hold for the ${\mathcal {A}}{\mathcal {W}}_r$-convergence. More details are given in Sect. 2.

### Outline

Section 2 presents the main result of this article, Theorem 2.6. We also provide a discussion of the result and give a sketch of its proof in order to help seeing through the technical details provided later on.

In Sect. 3 we provide certain technical lemmas which allow us to deal with difficulties specific to the adapted Wasserstein distance with more ease. They mainly explore properties of approximations and when addition (in a sense explained below) is continuous.

Section 4 focuses on the convex order. It deals with potential functions which are a convenient tool to address the convex order in dimension one.

Section 5 is devoted to the proof of the main theorem. Before entering into actual argument, we establish that is is enough to prove ${\mathcal {A}}{\mathcal {W}}_1$-convergence for irreducible pairs of marginals.

## Main result

Our main result is Theorem 2.6 below. Before stating it, we give a proposition which enlightens us why the conclusion of the theorem should be at least hoped for. We also state a generalisation of this proposition to Polish spaces. Then, we state a proposition which is a key result to argue that the theorem needs only to be proved when the limit pair is irreducible. Next, we state the theorem together with a sketch of its proof. It is understood that $(X,d_X)$ and $(Y,d_Y)$ denote arbitrary Polish spaces and that $(x_0,y_0)$ is a fixed element of $X\times Y$.

As already mentioned above, it is well-known (and easy to show) that when one considers convergent sequences of marginals $(\mu ^k)_{k\in \mathbb {N}}$, $(\nu ^k)_{k\in \mathbb {N}}$ (with equal masses) to $\mu ,\nu \in {\mathcal {M}}_r(X)$, then, informally speaking, we have^1^

2.1 $$\begin{aligned} \Pi (\mu ^k,\nu ^k) \underset{k\rightarrow +\infty }{\longrightarrow } \Pi (\mu ,\nu )\quad \text {in }{\mathcal {W}}_r, \end{aligned}$$

i.e., any sequence with convergent marginals has accumulation points in $\Pi (\mu ,\nu )$, and for any $\pi \in \Pi (\mu ,\nu )$ it holds

2.2 $$\begin{aligned} \inf _{\pi ^k\in \Pi (\mu ^k,\nu ^k)}{\mathcal {W}}_r^r(\pi ,\pi ^k)\le {\mathcal {W}}_r^r(\mu ,\mu ^k) + \mathcal W_r^r(\nu ,\nu ^k)\underset{k\rightarrow +\infty }{\longrightarrow }0. \end{aligned}$$

Indeed, if $\eta ^k\in \Pi (\mu ^k,\mu )$, resp. $\tau ^k\in \Pi (\nu ,\nu ^k)$ is optimal for ${\mathcal {W}}_r(\mu ^k,\mu )$, resp. ${\mathcal {W}}_r(\nu ,\nu ^k)$, then the measure $\eta ^k(dx^k,dx)\,\pi _x(dy)\,\tau ^k_y(dy^k)$ is a coupling between $\pi (dx,dy)$ and $\int _{(x,y)\in X\times Y}\eta ^k(dx^k,dx)\,\pi _x(dy)\,\tau ^k_y(dy^k)$ which belongs to $\Pi (\mu ^k,\nu ^k)$ and

$$\begin{aligned}&\inf _{\pi ^k\in \Pi (\mu ^k,\nu ^k)}{\mathcal {W}}_r^r(\pi ,\pi ^k)\\&\le \int _{X\times X\times Y\times Y}\left( d_X^r(x^k,x)+d_Y^r(y,y^k)\right) \eta ^k(dx^k,dx)\,\pi _x(dy)\,\tau ^k_y(dy^k)\\&=\mathcal W^r_r(\mu ,\mu ^k)+{\mathcal {W}}^r_r(\nu ,\nu ^k). \end{aligned}$$

The next two propositions establish (2.1) with respect to ${\mathcal {A}}{\mathcal {W}}_r$ for finite positive measures with common mass. The first one is formulated for $X=Y=\mathbb {R}$ and provides under mild assumptions an estimate of $\inf _{\pi ^k\in \Pi (\mu ^k,\nu ^k)}{\mathcal {A}}{\mathcal {W}}_r^r(\pi ,\pi ^k)$ with respect to the marginals as in (2.2). Its proof relies on unidimensional tools, which we recall here. For $\eta$ a probability distribution on $\mathbb {R}$, we denote by $F_\eta :x\mapsto \eta ((-\infty ,x])$ its cumulative distribution function, and by $F_\eta ^{-1}:(0,1)\rightarrow \mathbb {R}$ its quantile function defined for all $u\in (0,1)$ by

$$\begin{aligned} F_\eta ^{-1}(u)=\inf \{x\in \mathbb {R}\mid F_\eta (x)\ge u\}. \end{aligned}$$

The following properties are standard results (see for instance [37, Section 6] for proofs):

- $F_\eta$ is càdlàg i.e. right-continuous with left-hand limits, $F_\eta ^{-1}$ is càglàd i.e. left-continuous with right-hand limits;
- For all $$(x,u)\in \mathbb {R}\times (0,1)$$,
  2.3 $$\begin{aligned} F_\eta ^{-1}(u)\le x\iff u\le F_\eta (x), \end{aligned}$$
  which implies, using the notation $F_\eta (y-)$ for the left-hand limit of $F_\eta$ at $y\in \mathbb {R}$,
  2.4 $$\begin{aligned}&F_\eta (x-)<u\le F_\eta (x)\implies x=F_\eta ^{-1}(u), \end{aligned}$$
  2.5 $$\begin{aligned} \text {and}\quad&F_\eta (F_\eta ^{-1}(u)-)\le u\le F_\eta (F_\eta ^{-1}(u)); \end{aligned}$$
- For $\eta (dx)$-almost every $x\in \mathbb {R}$,
  2.6 $$\begin{aligned} 0<F_\eta (x),\quad F_\eta (x-)<1\quad \text {and}\quad F_\eta ^{-1}(F_\eta (x))=x; \end{aligned}$$
- The image of the Lebesgue measure on (0, 1) by $F_\eta ^{-1}$ is $\eta$.

The property (d) is referred to as the inverse transform sampling.

### Proposition 2.1

Let $\mu ,\mu ^k,\nu ,\nu ^k\in {\mathcal {M}}_r(\mathbb {R})$, $k\in \mathbb {N}$, be measures of equal masses such that $\mu ^k$ (resp. $\nu ^k)$ converges to $\mu$ (resp. $\nu$) in ${\mathcal {W}}_r$. Let $\pi \in \Pi (\mu ,\nu )$. Then:

- There exists a sequence $\pi ^k\in \Pi (\mu ^k,\nu ^k)$, $k\in \mathbb {N},$ converging to $\pi$ in ${\mathcal {A}}{\mathcal {W}}_r$;
- If for all $x \in \mathbb {R}$ and $k\in \mathbb {N}$ with $\mu ^k (\{ x \}) > 0$, there exists $x' \in \mathbb {R}$ such that
  $$\begin{aligned} \mu ((-\infty ,x'))\le \mu ^k((-\infty ,x))<\mu ^k((-\infty ,x])\le \mu ((-\infty ,x']) \end{aligned}$$
  (which is for instance always satisfied for $\mu ^k$ non-atomic) then
  2.7 $$\begin{aligned} {\mathcal {A}}{\mathcal {W}}_r^r (\pi ,\pi ^k) \le {\mathcal {W}}_r^r (\mu ,\mu ^k) + {\mathcal {W}}_r^r (\nu ,\nu ^k). \end{aligned}$$

### Remark 2.2

If $\pi$ is a martingale coupling, i.e. $\int _\mathbb {R}y'\,\pi _{x'}(dy')=x'$, $\mu (dx')$-almost everywhere, then for $\chi ^k\in \Pi (\mu ^k,\mu )$ an optimal coupling for ${\mathcal {A}}{\mathcal {W}}_r(\pi ^k,\pi )$, we have

$$\begin{aligned}&\int _\mathbb {R}\left| x-\int _\mathbb {R}y\,\pi ^k_x(dy)\right| ^r\,\mu ^k(dx)\\&\quad =\int _{\mathbb {R}\times \mathbb {R}}\left| x-\int _\mathbb {R}y\,\pi ^k_x(dy)\right| ^r\,\chi ^k(dx,dx')\\&\quad \le 2^{r-1}\int _{\mathbb {R}\times \mathbb {R}}\left( \vert x-x'\vert ^r+\left| x'-\int _\mathbb {R}y\,\pi ^k_x(dy)\right| ^r\right) \,\chi ^k(dx,dx')\\&\quad =2^{r-1}\int _{\mathbb {R}\times \mathbb {R}}\left( \vert x-x'\vert ^r+\left| \int _\mathbb {R}y'\,\pi _{x'}(dy')-\int _\mathbb {R}y\,\pi ^k_x(dy)\right| ^r\right) \,\chi ^k(dx,dx')\\&\quad \le 2^{r-1}\int _{\mathbb {R}\times \mathbb {R}}\left( \vert x-x'\vert ^r+{\mathcal {W}}_1^r(\pi ^k_x,\pi _{x'})\right) \,\chi ^k(dx,dx')\\&\quad \le 2^{r-1}{\mathcal {A}}{\mathcal {W}}_r^r(\pi ,\pi ^k)\underset{k\rightarrow +\infty }{\longrightarrow }0. \end{aligned}$$

In that sense, $\pi ^k,k\in \mathbb {N}$ is almost a sequence of martingale couplings.

In the setting of Proposition 2.1 and Remark 2.2, if $\mu ^k$ and $\nu ^k$ are also in the convex order and $\pi$ is a martingale coupling, then in view of Remark 2.2 one would naturally expect that $\pi ^k$ can be slightly modified into a martingale coupling and still converge to $\pi$ in ${\mathcal {A}}{\mathcal {W}}_r$. This actually requires a considerable amount of work and is the main message of Theorem 2.6 below. We mention that the previous proposition generalises to arbitrary Polish spaces *X* and *Y*, as the next proposition states, but unfortunately without providing an estimate.

### Proposition 2.3

Let $\mu ,\mu ^k\in {\mathcal {M}}_r(X),\nu ,\nu ^k\in {\mathcal {M}}_r(Y)$, $k\in \mathbb {N}$, all with equal masses and such that $\mu ^k$ (resp. $\nu ^k)$ converges to $\mu$ (resp. $\nu$) in ${\mathcal {W}}_r$. Let $\pi \in \Pi (\mu ,\nu )$. Then there exists a sequence $\pi ^k\in \Pi (\mu ^k,\nu ^k)$, $k\in \mathbb {N},$ converging to $\pi$ in ${\mathcal {A}}{\mathcal {W}}_r$.

The next proposition is a key ingredient which allows us to reduce the proof of Theorem 2.6 below to the case of irreducible pairs of marginals. For $\mu \in {\mathcal {M}}_1(\mathbb {R})$, we denote by $u_\mu$ its potential function, that is the map defined for all $y\in \mathbb {R}$ by $u_\mu (y) = \int _\mathbb {R}|y-x|\,\mu (dx)$ (see Sect. 4 for more details). We recall that a pair $(\mu ,\nu )$ of finite positive measures in convex order is called irreducible if $I=\{u_\mu <u_\nu \}$ is an interval and then, $\mu (I)=\mu (\mathbb {R})$ and $\nu ({\overline{I}})=\nu (\mathbb {R})$.

### Remark 2.4

If $(\mu ,\nu )$ is an irreducible pair of non-zero measures in the convex order and $a\in \mathbb {R}$ is such that $\nu ([a,+\infty ))=0$, then the convex order implies $\mu ([a,+\infty ))=0$, hence

$$\begin{aligned} u_\mu (a)=a-\int _\mathbb {R}x\,\mu (dx)=a-\int _\mathbb {R}y\,\nu (dy)=u_\nu (a), \end{aligned}$$

so $a\notin I$. Similarly, $\nu ((-\infty ,a])=0\implies a\notin I$. We deduce that $\nu$ must assign positive mass to any neighbourhood of each of the boundaries of *I*.

According to [13, Theorem A.4], for any pair $(\mu ,\nu )$ of probability measures in convex order, there exist $N\subset \mathbb {N}$ and a sequence $(\mu _n,\nu _n)_{n\in N}$ of irreducible pairs of sub-probability measures in convex order such that

$$\begin{aligned} \mu =\eta +\sum _{n\in N}\mu _n,\quad \nu =\eta +\sum _{n\in \mathbb {N}}\nu _n\quad \text {and}\quad \left\{ u_\mu<u_\nu \right\} =\bigcup _{n\in N}\left\{ u_{\mu _n}<u_{\nu _n}\right\} , \end{aligned}$$

where the union is disjoint and $\eta =\mu \vert _{\{u_\mu =u_\nu \}}$. The sequence $(\mu _n,\nu _n)_{n\in N}$ is unique up to rearrangement of the pairs and is called the decomposition of $(\mu ,\nu )$ into irreducible components. Moreover, for any martingale coupling $\pi \in \Pi _M(\mu ,\nu )$, there exists a unique sequence of martingale couplings $\pi _n\in \Pi _M(\mu _n,\nu _n)$, $n\in N$ such that

$$\begin{aligned} \pi =\chi +\sum _{n\in N}\pi _n, \end{aligned}$$

where $\chi =({\text {id}},{\text {id}})_*\eta$ and $*$ denotes the pushforward operation. This sequence satisfies

2.8 $$\begin{aligned} \forall n\in N,\quad \pi _n(dx,dy)=\mu _n(dx)\,\pi _x(dy). \end{aligned}$$

### Proposition 2.5

Let $(\mu ^k,\nu ^k)_{k\in \mathbb {N}}$ be a sequence of pairs of probability measures on the real line in convex order which converge to $(\mu ,\nu )$ in ${\mathcal {W}}_1$. Let $(\mu _n,\nu _n)_{n\in N}$ be the decomposition of $(\mu ,\nu )$ into irreducible components and $\eta =\mu \vert _{\{u_\mu =u_\nu \}}$. Then there exists for any $k\in \mathbb {N}$ a decomposition of $(\mu ^k,\nu ^k)$ into pairs of sub-probability measures $(\mu ^k_n,\nu ^k_n)_{n\in N}$, $(\eta ^k,\upsilon ^k)$ which are in convex order such that

2.9 $$\begin{aligned}&\eta ^k + \sum _{n\in N} \mu ^k_n = \mu ^k,\quad \upsilon ^k + \sum _{n\in N} \nu ^k_n = \nu ^k,\quad k\in \mathbb {N}, \end{aligned}$$

2.10 $$\begin{aligned}&\quad \lim _{k\rightarrow +\infty } \eta ^k = \eta ,\quad \lim _{k\rightarrow +\infty } \mu ^k_n = \mu _n,\quad \lim _{k\rightarrow +\infty } \nu ^k_n = \nu _n,\quad \lim _{k\rightarrow +\infty } \upsilon ^k =\eta \quad \text {in }{\mathcal {W}}_1.\nonumber \\ \end{aligned}$$

We can now state our main result, namely Theorem 2.6 below. Any martingale coupling whose marginals are approximated by probability measures in convex order can be approximated by martingale couplings with respect to the adapted Wasserstein distance.

### Theorem 2.6

Let $\mu ^k,\nu ^k\in {\mathcal {P}}_r(\mathbb {R})$, $k\in \mathbb {N}$, be in convex order and respectively converge to $\mu$ and $\nu$ in ${\mathcal {W}}_r$. Let $\pi \in \Pi _M(\mu ,\nu )$. Then there exists a sequence of martingale couplings $\pi ^k\in \Pi _M(\mu ^k,\nu ^k)$, $k\in \mathbb {N}$ converging to $\pi$ in ${\mathcal {A}}{\mathcal {W}}_r$.

### Proof

(Sketch of the proof) We will first argue that it is enough to consider the case $r=1$. Thanks to Proposition 2.5, we can also reduce the proof to the case of irreducible pairs of marginals $(\mu ,\nu )$, whose single irreducible component is denoted $(\ell ,\rho )=I$.

*Step 1.* Fix a martingale coupling $\pi \in \Pi _M(\mu ,\nu )$. When directly approximating $\pi$ we would face technical obstacles. First, for *K* a compact subset of *I*, $\mu \vert _K\times \pi _x$ is not necessarily compactly supported. Moreover, $\nu$ may put mass on the boundary of *I*. To overcome successively these two difficulties, the kernel $\pi _x$ is first compactified to a compact set $[-R,R]$, where $R>0$ (when $|\ell |\vee |\rho |<\infty$, one may choose *R* equal to this maximum), and then pushed forward by the map $y\mapsto \alpha (y-x)+x$, where $\alpha \in (0,1)$. This yields a martingale coupling $\pi ^{R,\alpha }$ close to $\pi$ and easier to approximate, between $\mu$ and a probability measure $\nu ^{R,\alpha }$ dominated by $\nu$ in the convex order. We find compact sets $K,L\subset I$ such that the restriction $\pi ^{R,\alpha }\vert _{K\times \mathbb {R}}$ is compactly supported on $K\times L$ and concentrated on $K\times \mathring{L}$, where $\mathring{L}$ denotes the interior of *L*. Since, by irreducibility, $\nu$ puts mass onto any neighbourhood of the boundary of *I*, $\nu ^{R,\alpha }$ assigns positive mass to two open sets $L_-$, $L_+$ on both sides of *K* with positive distance to *K*. This is summarised in Fig. 1, where *J* denotes a compact subset of *I* that is large enough.

*Step 2.* It is possible to find an approximating sequence $({\hat{\pi }}^k={\hat{\mu }}^k\times {\hat{\pi }}^k_x)_{k\in \mathbb {N}}$ for the sub-probability martingale coupling $\pi ^{R,\alpha }\vert _{K\times \mathbb {R}}$ from step 1. Unfortunately ${\hat{\pi }}^k$ is not necessarily a martingale coupling. Therefore, we free up some mass, and use the one available on the left and right of *K* in $L_-$ and $L_+$ to adjust the barycenters of the kernels $\pi ^k_x$. Hence we find a sequence $({\tilde{\pi }}^k={\hat{\mu }}^k\times {\tilde{\pi }}^k_x)_{k\in \mathbb {N}}$ of sub-probability martingale couplings approximating $\pi ^{R,\alpha }\vert _{K\times \mathbb {R}}$.

*Step 3.* By construction, up to multiplication by a factor smaller than and close to 1, the first marginal of ${\tilde{\pi }}^k$ satisfies ${\hat{\mu }}^k\le \mu ^k$. Moreover, its second marginal denoted ${\tilde{\nu }}^k$ is such that there exists a probability measure $\nu ^{R,\alpha ,k}$ which satisfies ${\tilde{\nu }}^k\le \nu ^{R,\alpha ,k}\le _c\nu ^k$. Then by using the uniform convergence of potential functions, we show that for *k* sufficiently large there exist sub-probability martingale couplings $\eta ^k\in \Pi _M(\mu ^k-{\hat{\mu }}^k,\nu ^{R,\alpha ,k}-{\tilde{\nu }}^k)$ so that the sum $\eta ^k+{\tilde{\pi }}^k$ is a martingale coupling in $\Pi _M(\mu ^k,\nu ^{R,\alpha ,k})$, where the second marginal is dominated by $\nu ^k$ in the convex order.

*Step 4.* In the last step, we use the inverse-transform martingale coupling between $\nu ^{R,\alpha ,k}$ and $\nu ^k$, see [37], to change $\eta ^k+{\tilde{\pi }}^k$ to a martingale coupling $\pi ^k \in \Pi (\mu ^k,\nu ^k)$. Finally, we estimate the ${\mathcal {A}}{\mathcal {W}}_1$-distance of $\pi$ to $\pi ^k$. $\square$

## On the adapted weak topology

We begin this section with a lemma on uniform integrability which will prove very handy throughout the paper. We formulate it for finite positive measures on *X*, but it is understood that $(X,x_0)$ is replaced with $(Y,y_0)$ for measures on *Y*.

### Lemma 3.1

Let $r\ge 1$ and $\mu \in {\mathcal {M}}_r(X)$. For $\epsilon >0$, let

3.1 $$\begin{aligned} I_\epsilon ^r(\mu ):=\sup _{\begin{array}{c} \tau \in {\mathcal {M}}(X) \\ \tau \le \mu ,~\tau (X)\le \epsilon \end{array}}\int _{X}d^r_X(x,x_0)\,\tau (dx). \end{aligned}$$

- $I_\epsilon ^r$ is monotone in $\mu$, i.e., $\mu \le \mu ' \in {\mathcal {M}}_r(X)$ implies that $I_\epsilon ^r(\mu ) \le I_\epsilon ^r(\mu ')$.
- The value of $I_\epsilon ^r(\mu )$ vanishes as $\epsilon \rightarrow 0$.
- For any $\mu ' \in {\mathcal {M}}_r(X)$ such that $\mu (X) = \mu '(X)$ we have
  3.2 $$\begin{aligned} I_\epsilon ^r(\mu )\le 2^{r-1}\left( I_\epsilon ^r(\mu ')+\mathcal W_r^r(\mu ,\mu ')\right) . \end{aligned}$$
- Let $\mu ,\mu ^k\in {\mathcal {M}}_r(X)$, $k\in \mathbb {N}$ be with equal masses such that $\mu ^k$ converges weakly to $\mu$. Then
  $$\begin{aligned} \mathcal W_r(\mu ^k,\mu )\underset{k\rightarrow +\infty }{\longrightarrow }0\iff \sup _{k\in \mathbb {N}}I^r_\varepsilon (\mu ^k)\underset{\varepsilon \rightarrow 0}{\longrightarrow }0\quad \text {and}\quad \sup _{k\in \mathbb {N}}\int _{X}d_X^r(x,x_0)\,\mu ^k(dx)<+\infty . \end{aligned}$$
- Finally, if $X=\mathbb {R}^d$ and $\mu \le _c\nu$ with $\nu \in \mathcal M_1(\mathbb {R}^d)$, then $I^1_\epsilon (\mu )\le I^1_\epsilon (\nu )$.

### Remark 3.2

If $\mu (X)\le \epsilon$, then $I_\epsilon ^r(\mu )$ is simply the *r*-th moment of $\mu$.

### Proof

The first point (a) is an easy consequence of the definition of $I^r_\epsilon$.

Next we check (b). Let $\mu \in \mathcal M_r(X)$ be such that $\mu (X)>0$. Since

3.3 $$\begin{aligned} I^r_\varepsilon (\mu )=\mu (X)I_{\frac{\varepsilon }{\mu (X)}}^r\left( \frac{\mu }{\mu (X)}\right) , \end{aligned}$$

to check convergence of $I^r_\varepsilon (\mu )$ to 0 as $\varepsilon \rightarrow 0$, we may suppose that $\mu \in {\mathcal {P}}_r(X)$. Let $\varepsilon \in (0,1)$. For $\eta \in {\mathcal {M}}_r(X)$, we denote by ${\overline{\eta }}$ the image of $\eta$ under the map $x\mapsto d_X^r(x,x_0)$. Let $\tau \in {\mathcal {M}}(X)$ be such that $\tau \le \mu$ and $0<\tau (X)\le \varepsilon$. Since $\tau \le \mu$, we have ${\overline{\tau }}\le {\overline{\mu }}$. Using (2.5) for the last inequality, we get for all $u\in (0,1)$

$$\begin{aligned} 1-F_{{\overline{\tau }}/\tau (X)}\left( F_{{\overline{\mu }}}^{-1} (1-\tau (X)u)\right)= & {} \frac{{\overline{\tau }}((F_{{\overline{\mu }}}^{-1}(1-\tau (X)u), +\infty ))}{\tau (X)}\\\le & {} \frac{{\overline{\mu }}((F_{{\overline{\mu }}}^{-1}(1-\tau (X)u), +\infty ))}{\tau (X)}\le u, \end{aligned}$$

hence $F_{{\overline{\tau }}/\tau (X)}(F_{{\overline{\mu }}}^{-1}(1-\tau (X)u))\ge 1-u$ and by (2.3), $F_{{\overline{\tau }}/\tau (X)}^{-1}(1-u)\le F_{{\overline{\mu }}}^{-1}(1-\tau (X)u)$. Using the inverse transform sampling, we deduce

3.4 $$\begin{aligned}&\int _Xd_X^r(x,x_0)\,\tau (dx)\nonumber \\&\quad =\tau (X)\int _0^1F_{{\overline{\tau }}/\tau (X)}^{-1}(1-u)\,du\nonumber \\&\quad \le \tau (X)\int _0^1F_{{\overline{\mu }}}^{-1}(1-\tau (X)u)\,du=\int _{1-\tau (X)}^1F_{{\overline{\mu }}}^{-1}(u)\,du\le \int _{1-\varepsilon }^1F_{{\overline{\mu }}}^{-1}(u)\,du. \end{aligned}$$

Hence $I^r_\varepsilon (\mu )\le \int _{1-\varepsilon }^{1}F_{{\overline{\mu }}}^{-1}(u)\,du$ where the right-hand side vanishes as $\varepsilon \rightarrow 0$ since, as $\mu \in {\mathcal {P}}_r(X)$, $\int _0^{1}F_{{\overline{\mu }}}^{-1}(u)\,du=\int _X d_X(x,x_0)^r\,\mu (dx)<+\infty$. Let us check the equality

3.5 $$\begin{aligned} I^r_\varepsilon (\mu )=\int _{1-\varepsilon }^{1}F_{{\overline{\mu }}}^{-1}(u)\,du, \end{aligned}$$

that will come in handy for the proof of claim (e) by setting

3.6 $$\begin{aligned} \tau ^*(dx)=\left( {\mathbb {1}}_{A\varepsilon }(x) +\frac{F_{{\overline{\mu }}}(y_\varepsilon )-(1-\varepsilon )}{\mu (B_\varepsilon )}{\mathbb {1}}_{B\varepsilon }(x)\right) \,\mu (dx), \end{aligned}$$

where

$$\begin{aligned} y_\varepsilon =F_{{\overline{\mu }}}^{-1}(1-\varepsilon ),\quad A_\varepsilon =\{x\in \mathbb {R}\mid d_X^r(x,x_0)>y_\varepsilon \}\quad \text {and}\quad B_\varepsilon =\{x\in \mathbb {R}\mid d_X^r(x,x_0)=y_\varepsilon \}, \end{aligned}$$

and the second summand of the right-hand side in (3.6) is taken to be zero if $\mu (B_\varepsilon )=0$. Since $A_\varepsilon \cap B_\varepsilon =\emptyset$ and, by (2.5),

$$\begin{aligned} \mu (B_\varepsilon )={{\overline{\mu }}}(\{y_\varepsilon \})=F_{{\overline{\mu }}}(y_\varepsilon )-F_{{\overline{\mu }}}(F_{{\overline{\mu }}}^{-1}(1-\varepsilon )-)\ge F_{{\overline{\mu }}}(y_\varepsilon )-(1-\varepsilon ), \end{aligned}$$

hence $\tau ^*\le \mu$. Moreover, $\overline{\tau ^*}$ is the measure dominated by $\overline{\mu }$ with mass equal to $\varepsilon$ which is the largest in stochastic order. Indeed, one easily checks that

$$\begin{aligned}&\overline{\tau ^*}(dy)=\mathbb {1}_{y>y_\varepsilon }\overline{\mu }(dy)+\left( F_{{\overline{\mu }}}(y_\varepsilon )-(1-\varepsilon )\right) \delta _{y_\varepsilon }(dy) \text{ so } \text{ that } \overline{\tau ^*}(\mathbb {R})=\varepsilon ,\\&\forall y\in \mathbb {R},\,F_{\overline{\tau ^*}/\varepsilon }(y)=\mathbb {1}_{y\ge y_\varepsilon }\frac{F_{\overline{\mu }}(y)-(1-\varepsilon )}{\varepsilon } \text{ and } \forall u\in (0,1),\,F^{-1}_{\overline{\tau ^*}/\varepsilon }(1-u){=}F_{\overline{\mu }}^{-1}(1{-}\varepsilon u). \end{aligned}$$

With the inverse transform sampling, the latter equality implies that

$$\begin{aligned} \int _Xd_X^r(x,x_0)\,\tau ^*(dx)=\varepsilon \!\!\!\int _0^1F^{-1}_{\overline{\tau ^*}/\varepsilon }(u)du=\varepsilon \!\!\int _0^1F_{\overline{\mu }}^{-1}(1-\varepsilon u)du =\int _{1-\varepsilon }^1F_{{\overline{\mu }}}^{-1}(u)\,du \end{aligned}$$

so that (3.5) holds.

To see (c), fix $\mu ' \in {\mathcal {M}}(X)$ with $\mu (X) = \mu '(X)$. We denote by $\pi (dx,dx')=\mu (dx)\,\pi _{x}(dx') \in \Pi (\mu ,\mu ')$ a ${\mathcal {W}}_r$-optimal coupling. Let $\tau \in {\mathcal {M}}(X)$ be such that $\tau \le \mu$ and $\tau (X)\le \epsilon$. Let $\tau '\in {\mathcal {M}}(X)$ be defined by

$$\begin{aligned} \tau '(dx')=\int _{x\in X}\pi _{x}(dx')\,\tau (dx). \end{aligned}$$

Since $\pi$ is element of $\Pi (\mu ,\mu ')$, we find $\tau '\le \mu '$ and $\tau (X)=\tau '(X)$. Then

$$\begin{aligned} \int _X d_X^r(x,x_0) \, \tau (dx)&\le 2^{r-1} \int _{X \times X} \left( d_X^r(x',x_0) + d_X^r(x,x')\right) \, \pi _{x}(dx')\, \tau (dx) \\&\le 2^{r-1} \left( I_\epsilon ^r(\mu ') + \int _{X\times X }d_X^r(x,x') \, \pi (dx,dx')\right) , \end{aligned}$$

which shows by optimality of $\pi$ the assertion.

We now show (d). Let $\mu ,\mu ^k\in {\mathcal {M}}_r(X)$ be with equal masses such that $\mu ^k$ converges weakly to $\mu$. According to (3.3), we may suppose that $\mu ,\mu ^k\in {\mathcal {P}}_r(X)$.

Suppose that ${\mathcal {W}}_r(\mu ^k,\mu )$ vanishes as *k* goes to $+\infty$. Then the sequence of the *r*-th moments of $\mu ^k$, $k\in \mathbb {N}$ is bounded since it converges to the *r*-th moment of $\mu$. Let $\eta >0$. Let $k_0\in \mathbb {N}$ be such that for all $k>k_0$, ${\mathcal {W}}_r^r(\mu ^k,\mu )<\eta$. Then (c) yields for $\varepsilon >0$

$$\begin{aligned} \sup _{k\in \mathbb {N}}I^r_\varepsilon (\mu ^k)\le \sum _{k\le k_0}I^r_\varepsilon (\mu ^k)+\sup _{k>k_0}I^r_\varepsilon (\mu ^k)\le \sum _{k\le k_0}I^r_\varepsilon (\mu ^k)+2^{r-1}(I^r_\varepsilon (\mu )+\eta ). \end{aligned}$$

According to (b) we then get

$$\begin{aligned} \limsup _{\varepsilon \rightarrow 0}\sup _{k\in \mathbb {N}}I^r_\varepsilon (\mu ^k)\le 2^{r-1}\eta . \end{aligned}$$

Since $\eta >0$ is arbitrary, we deduce that $\sup _{k\in \mathbb {N}}I^r_\varepsilon (\mu ^k)$ vanishes with $\varepsilon$.

Conversely, suppose that $\sup _{k\in \mathbb {N}}I^r_\varepsilon (\mu ^k)$ vanishes with $\varepsilon$ and the sequence of the *r*-th moments of $\mu ^k$, $k\in \mathbb {N}$ is bounded. By Skorokhod’s representation theorem, there exist random variables *X* and $X^k$, $k\in \mathbb {N}$, defined on a common probability space such that *X*, resp. $X^k$ is distributed according to $\mu$, resp. $\mu ^k$ and $X^k$ converges almost surely to *X*. Then for all $M>0$,

$$\begin{aligned}&\mathcal W_r^r(\mu ^k,\mu )\le \mathbb {E}[d_X^r(X^k,X)] \\&\quad =\mathbb {E}[d_X^r(X^k,X)\mathbb {1}_{\{d_X^r(X^k,X)<M\}}]+\mathbb {E}[d_X^r(X^k,X)\mathbb {1}_{\{d_X^r(X^k,X)\ge M\}}]. \end{aligned}$$

By the dominated convergence theorem, we deduce

$$\begin{aligned} \limsup _{k\rightarrow +\infty }\mathcal W_r^r(\mu ^k,\mu )\le \limsup _{k\rightarrow +\infty }\mathbb {E}[d_X^r(X^k,X)\mathbb {1}_{\{d_X^r(X^k,X)\ge M\}}]. \end{aligned}$$

Let us then prove that the right-hand side vanishes as *M* goes to $+\infty$. Let $\eta >0$. Let $\varepsilon >0$ be such that $I_\varepsilon ^r(\mu )+\sup _{k\in \mathbb {N}}I_\varepsilon ^r(\mu ^k)<\eta$. By Markov’s inequality, we have

$$\begin{aligned} \sup _{k\in \mathbb {N}}\mathbb {E}[\mathbb {1}_{\{d_X^r(X^k,X)\ge M\}}]\le \sup _{k\in \mathbb {N}}\frac{\mathbb {E}[d_X^r(X^k,X)]}{M}\le \frac{2^{r-1}}{M}\sup _{k\in \mathbb {N}}\int _X d_X^r(x,x_0)\,(\mu ^k+\mu )(dx), \end{aligned}$$

where the right-hand side vanishes as *M* goes to $+\infty$. Therefore, there exists $M_0>0$ such that for all $k\in \mathbb {N}$ and $M>M_0$,

$$\begin{aligned}&\mathbb {E}[d_X^r(X^k,X)\mathbb {1}_{\{d_X^r(X^k,X)\ge M\}}]\\&\quad \le 2^{r-1}\left( \mathbb {E}[d^r_X(X^k,x_0)\,\mathbb {1}_{\{d_X^r(X^k,X)\ge M\}}]+\mathbb {E}[d^r_X(x_0,X)\mathbb {1}_{\{d_X^r(X^k,X)\ge M\}}]\right) \\&\quad \le 2^{r-1}\left( I^r_\varepsilon (\mu ^k)+I^r_\varepsilon (\mu )\right) <2^{r-1}\eta . \end{aligned}$$

Therefore, for all $M>M_0$,

$$\begin{aligned} \limsup _{k\rightarrow +\infty }\mathbb {E}[d_X^r(X^k,X)\mathbb {1}_{\{d_X^r(X^k,X)\ge M\}}]\le 2^{r-1}\eta . \end{aligned}$$

Since $\eta$ is arbitrary, this proves the assertion.

Finally, we want to show (e). Let $X = \mathbb {R}^d$ and $\mu \le _c \nu$ with $\nu \in {\mathcal {M}}_1(\mathbb {R}^d)$. According to (3.3), we may suppose that $\mu ,\nu \in {\mathcal {P}}_1(\mathbb {R}^d)$. Again, we write $\overline{\mu }$ and $\overline{\nu }$ for the pushforward measures of $\mu$ and $\nu$ under the map $(x \mapsto |x-x_0|^r)$. First, we note that $\overline{\mu }$ is dominated by $\overline{\nu }$ in the increasing convex order. Indeed, let $f \in C(X)$ be convex and nondecreasing, then $x \mapsto f(|x-x_0|^r)$ constitutes a convex, continuous function. Thus,

$$\begin{aligned} \int _\mathbb {R}f(y) \, \overline{\mu }(dy) =\!\!\! \int _{\mathbb {R}^d} f(|x-x_0|^r) \, \mu (dx) \le \!\!\!\int _{\mathbb {R}^d} f(|x-x_0|^r) \, \nu (dx) = \!\!\! \int _\mathbb {R}f(y) \, \overline{\nu }(dy). \end{aligned}$$

The convex increasing order is characterised by the following family of inequalities (see for instance [2, Theorem 2.4]): for all $0 \le \varepsilon \le 1$,

$$\begin{aligned} \int _{1-\varepsilon }^{1} F_{\overline{\mu }}^{-1}(y) \, dy \le \int _{1-\varepsilon }^{1} F_{\overline{\nu }}^{-1}(y) \, dy. \end{aligned}$$

The identity (3.5) concludes the proof. $\square$

We now prove Proposition 2.1. A handy tool in the construction of the approximative sequence $(\pi ^k)_{k\in \mathbb {N}}$ are copulas. Recall that a two-dimensional copula is an element *C* of $\Pi (\lambda ,\lambda )$ where $\lambda$ is the uniform distribution on (0, 1). A coupling $\pi$ is an element of $\Pi (\mu ,\nu )$ if and only if it can be written as the push-forward of a copula *C* under the quantile map $(F_\mu ^{-1}, F_\nu ^{-1}) :(0,1) \times (0,1) \rightarrow \mathbb {R}\times \mathbb {R}$. Clearly, if *C* is a copula then $\pi = (F_\mu ^{-1}, F_\nu ^{-1})_*C$ is contained in $\Pi (\mu ,\nu )$. On the other hand, if $\pi \in \Pi (\mu ,\nu )$ is given, we can construct a copula *C* by

$$\begin{aligned} C(du,dv) = \mathbb {1}_{(0,1)}(u) \, du \, C_u(dv), \end{aligned}$$

where $C_u$ is given by

3.7 $$\begin{aligned} C_u = ((y,w) \mapsto F_\nu (y-) + w \nu (\{y\}))_*(\pi _{F_\mu ^{-1}(u)} \times \lambda ). \end{aligned}$$

In particular, we have that $u\mapsto C_u$ is constant on the jumps on $F_\mu$. The fact that the second marginal distribution of *C* is indeed uniformly distributed on (0, 1) is a direct consequence of the inverse transform sampling and the well-known result (see for instance [37, Lemma 6.6] for a proof) that for any $\eta \in {\mathcal {P}}(\mathbb {R})$,

3.8 $$\begin{aligned} ((z,w) \mapsto F_\eta (z-) + w \eta (\{z\}))_*(\eta \times \lambda ) = \lambda . \end{aligned}$$

Finally, we check the identity $\pi = (F_\mu ^{-1}, F_\nu ^{-1})_*C$. Let $w \in (0,1]$ and continue by distinguishing two cases: On the one hand, if $\nu (\{y\}) > 0$ then we have by (2.4)

3.9 $$\begin{aligned} F_\nu ^{-1}(F_\nu (y-) + w \nu (\{y\}))=y. \end{aligned}$$

On the other hand, we derive from (2.6) that (3.9) holds for $\nu$-almost every $y \in \{ z \in \mathbb {R}:\nu (\{ z \}) = 0 \}$. Hence, we obtain for $\lambda$-almost every $u \in (0,1)$

3.10 $$\begin{aligned} \pi _{F_\mu ^{-1}(u)}=(F_\nu ^{-1})_*C_u \end{aligned}$$

and conclude with $\pi = (F_\mu ^{-1}, F_\nu ^{-1})_*C$.

### Proof

(Proof of Proposition 2.1) Because of homogeneity of the ${\mathcal {A}}{\mathcal {W}}_r$- and ${\mathcal {W}}_r$-distances, we can suppose w.l.o.g. that $\mu ,\mu ^k,\nu ,\nu ^k$ and $\pi$ are probability measures. Let *C* be the copula defined by $C(du,dv)=\mathbb {1}_{(0,1)}(u)\,du\,C_u(dv)$, where $C_u$ is given by (3.7).

In order to define $\pi ^k$, we construct associated copulas $C^k$ where $u\mapsto C^k_u$ is constant on the jumps of $F_{\mu ^k}$. Let

$$\begin{aligned}&\theta _k :\mathbb {R}\times (0,1) \rightarrow (0,1),\ (x,w) \mapsto F_{\mu ^k}(x-) + w \mu ^k( \{ x \} ), \\&C_u^k(dv) = \int _{w = 0}^1 C_{\theta _k\left( F_{\mu ^k}^{-1}(u),w\right) }(dv) \, dw, \\&\pi ^k = (F_{\mu ^k}^{-1}, F_{\nu ^k}^{-1})_*C^k = (F_{\mu ^k}^{-1}, F_{\nu ^k}^{-1})_*(\mathbb {1}_{(0,1)}(u) \, du \, C_u^k(dv)). \end{aligned}$$

The fact that $C^k$ is a copula, and therefore $\pi ^k\in \Pi (\mu ^k,\nu ^k)$, is a direct consequence of (3.8) and the inverse transform sampling. Since $u \mapsto C_u$ and $u\mapsto C^k_u$ are constant on the jumps of $F_\mu$ and $F_{\mu ^k}$ respectively, reasoning like in the derivation of (3.10), we have for *du*-almost every *u* in (0, 1)

$$\begin{aligned} \pi _{F_{\mu }^{-1}(u)} = (F_{\nu }^{-1})_*C_u, \quad \pi ^k_{F_{\mu ^k}^{-1}(u)} = (F_{\nu ^k}^{-1})_*C^k_u. \end{aligned}$$

Moreover, since $(u \mapsto (F_\mu ^{-1}(u),F_{\mu ^k}^{-1}(u)))_*\lambda$ is a coupling between $\mu$ and $\mu ^k$, namely the comonotonous coupling, we have using the definition of ${\mathcal {A}}{\mathcal {W}}_r(\pi ,\pi ^k)$ as an infimum over $\Pi (\mu ,\mu ^k)$, cf. (1.2),

3.11 $$\begin{aligned} \begin{aligned} {\mathcal {A}}{\mathcal {W}}_r^r(\pi ,\pi ^k)&\le \int _0^1\left( \vert F_\mu ^{-1}(u) - F_{\mu ^k}^{-1}(u) \vert ^r + {\mathcal {W}}_r^r(\pi _{F_\mu ^{-1}(u)}, \pi ^k_{F_{\mu ^k}^{-1}(u)})\right) \, du \\&= {\mathcal {W}}_r^r(\mu ,\mu ^k) + \int _0^1 {\mathcal {W}}_r^r \left( (F_\nu ^{-1})_*C_u, (F_{\nu ^k}^{-1})_*C^k_u \right) \, du. \end{aligned} \end{aligned}$$

By Minkowski’s inequality we have

3.12 $$\begin{aligned} \begin{aligned} \left( \int _0^1 {\mathcal {W}}_r^r \left( (F_\nu ^{-1})_*C_u, (F_{\nu ^k}^{-1})_*C^k_u \right) \, du \right) ^{\frac{1}{r}}&\le \left( \int _0^1 {\mathcal {W}}_r^r \left( (F_\nu ^{-1})_*C_u, (F_\nu ^{-1})_*C^k_u \right) \, du \right) ^{\frac{1}{r}} \\&\quad + \left( \int _0^1 {\mathcal {W}}_r^r \left( (F_\nu ^{-1})_*C^k_u, (F_{\nu ^k}^{-1})_*C^k_u \right) \, du \right) ^{\frac{1}{r}}. \end{aligned} \end{aligned}$$

Since for any $\eta \in {\mathcal {P}}(\mathbb {R})$ the map $F_\eta ^{-1} \circ F_{C^k_u}^{-1}$ is non-decreasing, we have (see for instance [3, Lemma A.3]) that for *dw*-almost every $w\in (0,1)$,

$$\begin{aligned} F_{\eta }^{-1}(F_{C^k_u}^{-1}(w)) = F_{(F_{\eta }^{-1})_*C^k_u}^{-1}(w). \end{aligned}$$

Hence, we deduce

3.13 $$\begin{aligned} \begin{aligned}&\int _{(0,1)} {\mathcal {W}}_r^r \left( (F_\nu ^{-1})_*C^k_u, (F_{\nu ^k}^{-1})_*C^k_u \right) \, du\\&\quad = \int _{(0,1)}\int _{(0,1)}\vert F_\nu ^{-1} (F_{C^k_u}^{-1} (w) ) - F_{\nu ^k}^{-1} (F_{C^k_u}^{-1} (w) ) \vert ^r \, dw \, du \\&\quad = \int _{(0,1)}\int _{(0,1)} \vert F_\nu ^{-1} (v) - F_{\nu ^k}^{-1} (v) \vert ^r \, C^k_u(dv) \, du \\&\quad = \int _{(0,1)}\vert F_\nu ^{-1} (v) - F_{\nu ^k}^{-1} (v) \vert ^r \, dv = {\mathcal {W}}_r^r(\nu ,\nu ^k) \rightarrow 0, \end{aligned} \end{aligned}$$

where we used inverse transform sampling in the second equality. At this stage, we can already show (b) of Proposition 2.1. Indeed, the assumption made in (b) ensures that any jump of $F_{\mu _k}$ is included in a jump of $F_\mu$. We already noted that $u\mapsto C_u$ is constant on the jumps of $F_\mu$ and therefore also constant on the jumps of $F_{\mu ^k}$. This yields for all $u,w\in (0,1)$ that $C_{\theta _k(F_{\mu ^k}^{-1}(u),w)}=C_u$ and particularly $C^k_u=C_u$, which causes the first term on the right-hand side of (3.12) to vanish. Then the estimate (2.7) follows immediately from (3.11), (3.12) and (3.13).

To obtain (a) and in view of (3.11), (3.12) and (3.13), it is sufficient to show

$$\begin{aligned} \int _0^1 {\mathcal {W}}_r^r \left( (F_\nu ^{-1})_*C_u, (F_\nu ^{-1})_*C^k_u \right) \, du \rightarrow 0. \end{aligned}$$

This is achieved in two steps: First, we show for *du*-almost every $u \in (0,1)$ that

3.14 $$\begin{aligned} {\mathcal {W}}_r((F_\nu ^{-1})_*C_u, (F_\nu ^{-1})_*C_u^k) \rightarrow 0. \end{aligned}$$

Second, we prove that

3.15 $$\begin{aligned} u \mapsto {\mathcal {W}}_r^r((F_\nu ^{-1})_*C_u, (F_\nu ^{-1})_*C_u^k) \quad k \in \mathbb {N}, \end{aligned}$$

is uniformly integrable on (0, 1) with respect to $\lambda$.

To show (3.14), note that $\mathcal W_r$-convergence is already determined by a countable family ${\mathcal {C}} \subset \Phi _r(\mathbb {R})$ (see [25, Theorem 4.5.(b)]). For this reason, it is sufficient to show that for all $f \in {\mathcal {C}}$, for *du*-almost every $u\in (0,1)$,

3.16 $$\begin{aligned} \int _{(0,1)} f(F_\nu ^{-1}(v)) \, C_u^k(dv) \rightarrow g(u):=\int _{(0,1)} f(F_\nu ^{-1}(v)) \, C_u(dv),\quad k\rightarrow +\infty ,\nonumber \\ \end{aligned}$$

where the integrals are *du*-almost everywhere well defined because of the inverse transform sampling, the fact that $f\in \Phi _r(\mathbb {R})$ and $\nu \in {\mathcal {P}}_r(\mathbb {R})$. For $u\in (0,1)$, let $x_u = F_\mu ^{-1}(u)$ and $x^k_u = F_{\mu ^k}^{-1}(u)$. Let $\mathcal U\subset (0,1)$ be the set of continuity points of $F_\mu ^{-1}$ and define

$$\begin{aligned}&{\mathcal {U}}_c=\{u\in {\mathcal {U}}\mid F_\mu \text { is continuous at }x_u\}\quad \text {and}\\&\quad {\mathcal {U}}_d=\{u\in \mathcal U\backslash {\mathcal {U}}_c\mid u\in (F_\mu (x_u-),F_\mu (x_u))\}. \end{aligned}$$

By monotonicity of $F_\mu ^{-1}$, the complement of ${\mathcal {U}}$ in (0, 1) is at most countable, and since $\mu$ has countably many atoms, the complement of ${\mathcal {U}}_d$ in $\mathcal U\backslash {\mathcal {U}}_c$ is also at most countable. We deduce that it is sufficient to show (3.16) for *du*-almost all $u\in {\mathcal {U}}_c\cup {\mathcal {U}}_d$. Let then $u\in {\mathcal {U}}$. If $\mu ^k(\{x^k_u\})=0$, then $C_u^k = C_u$ and

$$\begin{aligned} \int _{(0,1)} f(F_\nu ^{-1}(v)) \, C_u^k(dv)=g(u). \end{aligned}$$

From now on and until (3.16) is proved, we suppose w.l.o.g. that $\mu ^k(\{x^k_u\})>0$ for all $k\in \mathbb {N}$. Then

3.17 $$\begin{aligned} \int _{(0,1)} f(F_\nu ^{-1}(v)) \, C_u^k(dv) =\frac{1}{\mu ^k(\{ x_u^k \})}\int _{F_{\mu ^k}(x_u^k - )}^{F_{\mu ^k}(x_u^k)} g(w) \, dw . \end{aligned}$$

Define $l_k = \inf _{n \ge k} x_u^n$ and $r_k = \sup _{n \ge k} x_u^n$. Since $u \in {\mathcal {U}}$ we find $l_k \nearrow x_u$ and $r_k \searrow x_u$ when *k* goes to $+\infty$. Due to right continuity of $F_\mu$ and left continuity of $x \mapsto F_\mu (x-)$ we have

$$\begin{aligned} F_\mu (x_u-) = \lim _p F_\mu (l_p -)\quad \text {and}\quad \lim _p F_\mu (r_p) = F_\mu (x_u). \end{aligned}$$

By Portmanteau’s theorem and monotonicity of cumulative distribution functions we have

$$\begin{aligned} F_\mu (l_p-)\le & {} \liminf _k F_{\mu ^k}(l_p -)\le \liminf _k F_{\mu ^k}(x^k_u-)\le \limsup _k F_{\mu ^k}(x^k_u) \\\le & {} \limsup _k F_{\mu ^k}(r_p) \le F_\mu (r_p). \end{aligned}$$

By taking the limit $p\rightarrow +\infty$, we find

3.18 $$\begin{aligned} F_\mu (x_u-)\le \liminf _k F_{\mu ^k}(x^k_u-) \le \limsup _k F_{\mu ^k}(x_u^k) \le F_\mu (x_u). \end{aligned}$$

By (2.5), the interval $[F_{\mu ^k}(x^k_u-),F_{\mu ^k}(x^k_u)]$ contains *u*, and if $u\in {\mathcal {U}}_c$, then (3.18) implies that its length $\mu ^k(\{x^k_u\})$ vanishes when *k* goes to $+\infty$. Consequently, (3.17) and the Lebesgue differentiation theorem yield that for *du*-almost every $u\in {\mathcal {U}}_c$,

$$\begin{aligned} \int _{(0,1)} f(F_\nu ^{-1}(v)) \, C_u^k(dv) \rightarrow g(u). \end{aligned}$$

Suppose now $u\in {\mathcal {U}}_d$ and define

$$\begin{aligned} a_k = F_{\mu ^k}(x_u^k-) \vee F_\mu (x_u-),\quad b_k = F_{\mu ^k}(x_u^k) \wedge F_\mu (x_u). \end{aligned}$$

Note that on the interval $(a_k,b_k)$ the function *g* is constant equal to *g*(*u*), so (3.17) amounts to

$$\begin{aligned}&\int _{(0,1)} f(F_\nu ^{-1}(v)) \, C_u^k(dv) \\&\quad = \frac{1}{\mu ^k(\{x_u^k\})} \left( \int _{b_k}^{F_{\mu ^k}(x_u^k)} g(w) \, dw + \int _{a_k}^{b_k} g(u) \, dw + \int _{F_{\mu ^k}(x_u^k-)}^{a_k} g(w) \, dw \right) . \end{aligned}$$

According to (3.18),

3.19 $$\begin{aligned} a_k - F_{\mu ^k}(x_u^k-)\rightarrow 0\quad \text {and}\quad F_{\mu ^k}(x_u^k) - b_k\rightarrow 0,\quad k\rightarrow +\infty . \end{aligned}$$

Moreover, having (2.5) in mind it is clear that

3.20 $$\begin{aligned} \begin{aligned} F_{\mu ^k}(x^k_u-)<a_k \implies \mu ^k(\{x^k_u\})\ge u-F_\mu (x_u-),\\ \text {and}\quad b_k<F_{\mu ^k}(x^k_u) \implies \mu ^k(\{x^k_u\})\ge F_\mu (x_u)-u. \end{aligned} \end{aligned}$$

Using the latter fact and the equality

$$\begin{aligned} b_k-a_k=\mu _k(\{x^k_u\})-(F_{\mu _k}(x^k_u)-b_k)-(a_k-F_{\mu _k}(x^k_u-)), \end{aligned}$$

we get

$$\begin{aligned} 1-\frac{F_{\mu _k}(x^k_u)-b_k}{F_\mu (x_u)-u}-\frac{a_k-F_{\mu _k}(x^k_u-)}{u-F_\mu (x_u-)}\le \frac{b_k-a_k}{\mu _k(\{x^k_u\})}\le 1. \end{aligned}$$

Hence by (3.19) we have $\frac{b_k-a_k}{\mu _k(\{x^k_u\})}\rightarrow 1$ as *k* goes to $+\infty$, which implies that $\frac{1}{\mu ^k(\{x^k_u\})}\int _{a_k}^{b_k}g(u)\,dw\rightarrow g(u)$ as $k\rightarrow +\infty$. Therefore, we just have to show that

3.21 $$\begin{aligned} \frac{1}{\mu ^k(\{x_u^k\})} \left( \int _{b_k}^{F_{\mu ^k}(x_u^k)} g(w) \, dw + \int _{F_{\mu ^k}(x_u^k-)}^{a_k} g(w) \, dw \right) \rightarrow 0,\quad k\rightarrow +\infty . \end{aligned}$$

Note that we can assume w.l.o.g. that for all $k\in \mathbb {N}$ either $F_{\mu ^k}(x^k_u-) < a_k$ or $b_k < F_{\mu ^k}(x^k_u)$. Let $d=(u-F_\mu (x_u-))\wedge (F_\mu (x_u)-u)$, which is positive since $u\in {\mathcal {U}}_d$. Then we have by (3.20)

3.22 $$\begin{aligned} \begin{aligned}&\frac{1}{\mu ^k(\{x_u^k\})} \left| \int _{b_k}^{F_{\mu ^k}(x_u^k)} g(w) \, dw + \int _{F_{\mu ^k}(x_u^k-)}^{a_k} \right. g(w) \, dw \Bigg |\\&\qquad \qquad \qquad \qquad \qquad \qquad \qquad \quad \le \frac{1}{d} \left| \int _{b_k}^{F_{\mu ^k}(x_u^k)} g(w) \, dw + \int _{F_{\mu ^k}(x_u^k-)}^{a_k} g(w) \, dw \right| . \end{aligned} \end{aligned}$$

By the inverse transform sampling and the facts that $f\in \Phi _r(\mathbb {R})$ and $\nu \in {\mathcal {P}}_r(\mathbb {R})$, we have $\int _0^1\vert g(w)\vert \,dw=\int _\mathbb {R}\vert f(y)\vert \,\nu (dy)<+\infty$. Then (3.21) is a direct consequence of (3.22), (3.19) and the dominated convergence theorem. Hence (3.14) is proved for *du*-almost every $u\in (0,1)$.

Next, we show uniform integrability of (3.15). We can estimate

$$\begin{aligned} {\mathcal {W}}_r^r((F_\nu ^{-1})_*C_u, (F_\nu ^{-1})_*C_u^k) \le 2^{r-1} \left( \int _{(0,1)} |F_\nu ^{-1}(v)|^r \, C_u(dv) +\!\! \int _{(0,1)} |F_\nu ^{-1}(v)|^r \, C_u^k(dv) \right) . \end{aligned}$$

Since by the inverse transform sampling we have

$$\begin{aligned} \int _{(0,1)}\int _{(0,1)} |F_\nu ^{-1}(v)|^r \,C_u(dv) \, du = \int _\mathbb {R}\vert y \vert ^r \, \nu (dy) < \infty , \end{aligned}$$

it is enough to show uniform integrability of $u \mapsto \int _{(0,1)} |F_\nu ^{-1}(v)|^r \, C_u^k(dv)$, $k\in \mathbb {N}$.

On the one hand, using the inverse transform sampling and $\nu \in {\mathcal {P}}_r(\mathbb {R})$, we have

$$\begin{aligned} {\forall k\in \mathbb {N},}\;\int _{(0,1)}\int _{(0,1)}\vert F_\nu ^{-1}(v)\vert ^r C^k_u(dv)\,du=\int _\mathbb {R}\vert y\vert ^r\,\nu (dy)<+\infty . \end{aligned}$$

On the other hand, let $\epsilon > 0$ and *A* be a measurable subset of (0, 1) such that $\lambda (A) < \epsilon$. We have

$$\begin{aligned} \int _A \int _{(0,1)} |F_\nu ^{-1}(v)|^r \, C_u^k(dv) \, du = \int _\mathbb {R}|y|^r \, (F_\nu ^{-1})_*\tau ^k(dy), \end{aligned}$$

where $\tau ^k(dv) = \int _{u = 0}^1 \mathbb {1}_A(du) \, C_u^k(dv) \, du$. Note that $\tau ^k \le \lambda$, $(F_\nu ^{-1})_*\tau ^k \le \nu$ and $(F_\nu ^{-1})_*\tau ^k(\mathbb {R}) = \tau ^k((0,1)) = \lambda (A)$. Therefore,

$$\begin{aligned} \sup _{\begin{array}{c} A \in {\mathcal {B}}((0,1)), \\ \lambda (A) \le \epsilon \end{array}} \sup _k \int _A \int _{(0,1)} |F_\nu ^{-1}(v)|^r \, C_u^k(dv) \, du \le I_\epsilon ^r(\nu ), \end{aligned}$$

where $I^r_\varepsilon (\nu )$ is defined by (3.1). By Lemma 3.1, the right-hand side converges to 0 with $\epsilon \rightarrow 0$. This yields uniform integrability of (3.15), which completes the proof. $\square$

As mentioned in Sect. 2, Proposition 2.1 generalises to Polish spaces. Unsurprisingly, the proof of Proposition 2.3 requires radically different tools from its unidimensional equivalent. In particular, we need to recall the so-called Weak Optimal Transport (WOT) problem introduced by Gozlan, Roberto, Samson and Tetali [29] and studied in [28]. Let $C:X\times {\mathcal {P}}_r(Y)\rightarrow \mathbb {R}_+$ be nonnegative, continuous, strictly convex in the second argument and such that there exists a constant $K>0$ which satisfies

3.23 $$\begin{aligned} \forall (x,p)\in X\times {\mathcal {P}}_r(Y),\quad C(x,p)\le K\left( 1+d_X^r(x,x_0)+\int _Yd_Y^r(y,y_0)\,p(dy)\right) .\quad \end{aligned}$$

Then the WOT problem consists in minimising

WOT $$\begin{aligned} V_C(\mu ,\nu ):=\inf _{\pi \in \Pi (\mu ,\nu )}\int _X C(x,\pi _x)\,\mu (dx). \end{aligned}$$

In view of the definition (1.3) of the adapted Wasserstein distance which involves measures on the extended space $X\times {\mathcal {P}}(Y)$, it is natural to consider an extension of (WOT) which also involves this space. Hence we also consider the extended problem

WOT' $$\begin{aligned} V'_C(\mu ,\nu ):=\inf _{P\in \Lambda (\mu ,\nu )}\int _{X\times \mathcal P(Y)}C(x,p)\,P(dx,dp), \end{aligned}$$

where $\Lambda (\mu ,\nu )$ is the set of couplings between $\mu$ and an arbitrary measure on ${\mathcal {P}}(Y)$ with mean $\nu$, that is

3.24 $$\begin{aligned} \Lambda (\mu ,\nu )=\left\{ P\in {\mathcal {P}}(X\times \mathcal P(Y))\mid \int _{(x',p)\in X\times \mathcal P(Y)}\delta _{x'}(dx)\,p(dy)\,P(dx',dp) \in \Pi (\mu ,\nu )\right\} .\nonumber \\ \end{aligned}$$

### Remark 3.3

We gather here useful results on weak transport problems which hold under the standing assumptions on *C*:

- according to [8, Theorem 1.2] and the paragraph following this theorem, (WOT) admits a unique minimiser $\pi ^*$;
- As a consequence of the necessary optimality condition [9, Theorem 2.2], $J(\pi ^*)$ is the only minimiser of (WOT’). Indeed, if we assume the opposite then there is a minimizer $P^*\in \Lambda (\mu ,\nu )$ of (WOT’) which does not lie in the image of $\Pi (\mu ,\nu )$ under *J*. Hence, any measurable set ${\mathcal {A}} \subset X \times {\mathcal {P}}_r(Y)$ with $P^*({\mathcal {A}}) = 1$ contains $(x,p), (x,q) \in {\mathcal {A}}$ with $p \ne q$. Due to strict convexity of *C* in its second argument, we find
  $$\begin{aligned} C\left( x, \frac{p + q}{2} \right) < \frac{1}{2} \left( C(x,p) + C(x,q) \right) . \end{aligned}$$
  Since ${\mathcal {A}}$ was an arbitrary set supporting $P^*$, the strict inequality above contradicts the necessary optimality condition in [9, Theorem 2.2];
- $$V(\mu ,\nu )=V'(\mu ,\nu )$$ [8, Lemma 2.1];
- Stability of (WOT) and (WOT’): Let $\mu ^k\in {\mathcal {P}}_r(X),\nu ^k\in {\mathcal {P}}_r(Y)$, $k\in \mathbb {N}$ converge respectively to $\mu \in {\mathcal {P}}_r(X)$ and $\nu \in {\mathcal {P}}_r(Y)$ in ${\mathcal {W}}_r$. For $k\in \mathbb {N}$, let $\pi ^k\in \Pi (\mu ^k,\nu ^k)$ be optimal for $V(\mu ^k,\nu ^k)$. Then $\pi ^k$, resp. $J(\pi ^k)$, converges to the unique minimiser $\pi ^*$, resp. $J(\pi ^*)$, in ${\mathcal {W}}_r$ [9, Theorem 1.3 and Corollary 2.9]. In particular, this shows that $\pi ^k$ converges to $\pi ^*$ even in ${\mathcal {A}}{\mathcal {W}}_r$.

### Proof of Proposition 2.3

Since $\nu \in {\mathcal {P}}_r(Y)$, we have that

3.25 $$\begin{aligned} \int _X\int _Yd_Y^r(y,y_0)\,\pi _x(dy)\,\mu (dx)=\int _Yd_Y^r(y,y_0)\,\nu (dy)<+\infty , \end{aligned}$$

hence up to a modification on a $\mu$-null set, we can suppose w.l.o.g. that for all $x\in X$, $\pi _x\in {\mathcal {P}}_r(Y)$. Let $\varepsilon >0$ and $y_0\in Y$. Define for $R > 0$ the $\mathcal W_r$-open ball $B_R$ of radius $R^{1/r}$ and centre $\delta _{y_0}$ and the set By (3.25) again, $\mu$ is concentrated on $\bigcup _{R > 0}A_R$ and we can choose *R* large enough such that

$$\begin{aligned} \mu (X\backslash A_R)<\epsilon . \end{aligned}$$

Since $\mu$ is a probability measure on the Polish space *X*, it is a Radon measure. Moreover, ${\mathcal {P}}_r(Y)$ endowed with ${\mathcal {W}}_r$ is a separable metric space, hence it is second-countable. Therefore we can apply Lusin’s theorem to the map $X\ni x\mapsto \pi _x\in {\mathcal {P}}_r(Y)$ in order to deduce the existence of a closed set $F\subset A_R$ such that

$$\begin{aligned} \mu (X\backslash F)< \epsilon \quad \text {and}\quad x\mapsto \pi _x\text { restricted to { F} is continuous}. \end{aligned}$$

Let $\widetilde{{\mathcal {M}}}_r(Y)$ be the linear space of all finite signed measures on *Y*, the positive and negative parts of which are contained in ${\mathcal {M}}_r(Y)$, equipped with the weak topology induced by $\Phi _r(Y)$. Since weak topologies are locally convex, an extension of Tietze’s theorem [24, Theorem 4.1] yields the existence of a continuous map $x\mapsto {\overline{\pi }}_x$ defined on *X* with values in $\widetilde{{\mathcal {M}}}_r(Y)$ such that ${\overline{\pi }}_x=\pi _x$ for all $x\in F$ and

$$\begin{aligned} \{{\overline{\pi }}_x\mid x\in X\}\subset {\text {co}}\{\pi _x\mid x\in F\}\subset B_R, \end{aligned}$$

where ${\text {co}}$ denotes the convex hull.

Next, we define a nonnegative, continuous, strictly convex in the second argument function which satisfies a condition of the form (3.23) in order to use the results on weak transport problems detailed in Remark 3.3. Let $\{ g_k\mid k \in \mathbb {N}\}\subset \Phi _1(Y)$ be a family of 1-Lipschitz continuous functions and absolutely bounded by 1, which separates ${\mathcal {P}}(Y)$ (see [25, Theorem 4.5.(a)]). We have for any pair $p,p' \in {\mathcal {P}}(Y)$, $p \ne p'$ that there is $l \in \mathbb {N}$ such that

3.26 $$\begin{aligned} \int _Y g_l(y) \, p(dy) \ne \int _Y g_l(y) \, p'(dy). \end{aligned}$$

Define $C:X\times {\mathcal {P}}_r(Y)\rightarrow \mathbb {R}_+$ for all $(x,p)\in X\times {\mathcal {P}}_r(Y)$ by

$$\begin{aligned} C(x,p):=\rho ({\overline{\pi }}_x,p)+\sum _{k \in \mathbb {N}} \frac{1}{2^k} \left| \int _Y g_k(y)\,{\overline{\pi }}_x(dy)-\int _Yg_k(y)\,p(dy)\right| ^2, \end{aligned}$$

where $\rho :{\mathcal {P}}(Y)\times {\mathcal {P}}(Y)\rightarrow [0,1]$ is defined for all $p,p'\in {\mathcal {P}}(Y)$ by

$$\begin{aligned} \rho (p,p')=\inf _{\chi \in \Pi (p,p')}\int _{Y\times Y}(d_Y(y,y')\wedge 1)\,\chi (dy,dy'). \end{aligned}$$

Since $\rho$ can be interpreted as a Wasserstein distance with respect to a bounded distance, it is immediate that it is a metric on ${\mathcal {P}}(Y)$ which induces the weak convergence topology. On the one hand, the map $(x,p)\mapsto \rho ({\overline{\pi }}_x,p)$ is continuous by continuity of $x\mapsto {\overline{\pi }}_x$. On the other hand, by Kantorovich and Rubinstein’s duality theorem and Jensen’s inequality, we have for all $(x,p),(x',p')\in X\times \mathcal P_r(Y)$

$$\begin{aligned}&\sum _{k \in \mathbb {N}} \frac{1}{2^k} \left| \left| \int _Y g_k(y) \, {\overline{\pi }}_x(dy) - \int _Y g_k(y) \, p(dy)\right| ^2 -\left| \int _Y g_k(y) \, {\overline{\pi }}_{x'}(dy)-\int _Y g_k(y) \, p'(dy)\right| ^2 \right| \\&\quad =\sum _{k \in \mathbb {N}} \frac{1}{2^k} \left| \int _Y g_k(y) \, {\overline{\pi }}_x(dy) - \int _Y g_k(y) \, p(dy)+\int _Y g_k(y) \, {\overline{\pi }}_{x'}(dy)-\int _Y g_k(y) \, p'(dy) \right| \\&\quad \qquad \times \left| \int _Y g_k(y) \, {\overline{\pi }}_x(dy) - \int _Y g_k(y) \, p(dy)-\int _Y g_k(y) \, {\overline{\pi }}_{x'}(dy)+\int _Y g_k(y) \, p'(dy) \right| \\&\quad \le \sum _{k \in \mathbb {N}} \frac{4}{2^k} \left( \left| \int _Y g_k(y) \, {\overline{\pi }}_x(dy)-\int _Yg_k(y)\,{\overline{\pi }}_{x'}(dy)\right| +\left| \int _Yg_k(y)\,p(dy)-\int _Yg_k(y)\,p'(dy)\right| \right) \\&\quad \le 8\left( {\mathcal {W}}_1({\overline{\pi }}_x,{\overline{\pi }}_{x'})+\mathcal W_1(p,p')\right) \le 8\left( \mathcal W_r({\overline{\pi }}_x,{\overline{\pi }}_{x'})+{\mathcal {W}}_r(p,p')\right) , \end{aligned}$$

where the right-hand side vanishes when $(x',p')$ converges to (*x*, *p*) by continuity of $x\mapsto {\overline{\pi }}_x$. We deduce that *C* is continuous.

Note that $\rho$ is convex in the second argument. Therefore, to obtain strict convexity of $C(x,\cdot )$ in the second argument, it is sufficient to verify that

$$\begin{aligned} F(p) = \sum _{k \in \mathbb {N}} \frac{1}{2^k} \left| \int _Y g_k(y) \, p(dy)\right| ^2 \end{aligned}$$

is strictly convex. Let $p,p' \in {\mathcal {P}}(Y)$, $p \ne p'$ and $l \in \mathbb {N}$ such that (3.26) holds. Hence, strict convexity of the square proves

$$\begin{aligned}&\left| \alpha \int _Y g_l(y)\, p(dy) + (1 - \alpha ) \int _Y g_l(y) \, p'(dy) \right| ^2\\&\quad \quad \quad \qquad \qquad \qquad < \alpha \left| \int _Y g_l(y)\, p(dy) \right| ^2 + (1 - \alpha ) \left| \int _Y g_l(y)\, p'(dy) \right| ^2, \end{aligned}$$

which yields strict convexity of *F* on ${\mathcal {P}}(Y)$.

Moreover, we have for all $(x,p)\in X\times {\mathcal {P}}_r(Y)$, $C(x,p)\le 1+8=9$, hence *C* satisfies (3.23). Remember the definitions of $V_C$ and $V'_C$ given in (WOT) and (WOT’). Since for all $x\in F$, $C(x,\pi _x)=C(x,{\overline{\pi }}_x)=0$, we have

$$\begin{aligned} V_C(\mu ,\nu ) \le \int _{X \backslash F} C(x,\pi _x)\, \mu (dx)<9\varepsilon . \end{aligned}$$

Let $\pi ^{*,\varepsilon }\in \Pi (\mu ,\nu )$ be optimal for $V_C(\mu ,\nu )$. For $P,P'\in {\mathcal {P}}(X\times {\mathcal {P}}(Y))$, let

$$\begin{aligned} {\tilde{\rho }}(P,P')=\inf _{\chi \in \Pi (P,P')}\int _{X\times \mathcal P(Y)\times X\times \mathcal P(Y)}\left( \left( d_X(x,x')+\rho (p,p')\right) \wedge 1\right) \,\chi (dx,dp,dx',dp'). \end{aligned}$$

Since $\mu (dx)\,\delta _{\pi _x}(dp)\,\delta _{x}(dx')\,\delta _{\pi ^{*,\varepsilon }_{x'}}(dp')$ is a coupling between $J(\pi )$ and $J(\pi ^{*,\varepsilon })$, we can estimate

$$\begin{aligned}&{\tilde{\rho }}(J(\pi ),J(\pi ^{*,\varepsilon }))\\&\quad \le \int _X\rho (\pi _x,\pi ^{*,\varepsilon }_x)\,\mu (dx)\\&\quad \le \int _F\rho (\pi _x,\pi ^{*,\varepsilon }_x)\,\mu (dx)\\&\quad \quad +\int _{X\setminus F}\int _Y(d_Y(y,y_0)\wedge 1)\,(\pi _x+\pi ^{*,\varepsilon }_x)(dy)\,\mu (dx)\\&\quad \le V_C(\mu ,\nu )+2\varepsilon <11\varepsilon . \end{aligned}$$

For $k\in \mathbb {N}$, let $\pi ^{k,\varepsilon }\in \Pi (\mu ^k,\nu ^k)$ be optimal for $V_C(\mu ^k,\nu ^k)$. Then $J(\pi ^{k,\varepsilon })$ is optimal for $V'_C(\mu ^k,\nu ^k)$ by Remark 3.3 (b), and converges to $J(\pi ^{*,\varepsilon })$ in ${\mathcal {W}}_r$ and therefore weakly by Remark 3.3 (d). Then we get

3.27 $$\begin{aligned}&\limsup _{k\rightarrow +\infty }{\tilde{\rho }}(J(\pi ^{k,\varepsilon }),J(\pi )) \nonumber \\&\quad \le \limsup _{k \rightarrow + \infty }\left( {\tilde{\rho }}(J(\pi ^{k,\varepsilon }),J(\pi ^{*,\varepsilon }))+{\tilde{\rho }}(J(\pi ^{*,\varepsilon }),J(\pi ))\right) \le 11\varepsilon . \end{aligned}$$

So far $\epsilon > 0$ was arbitrary. Therefore, there exists a strictly increasing sequence $(k_N)_{N\in \mathbb {N}^*}$ of positive integers such that

$$\begin{aligned} \forall N \in \mathbb {N}^*,\quad \forall k \ge k_N, \quad {\tilde{\rho }}(J(\pi ^{k,{1/N}}),J(\pi )) \le {\frac{12}{N}}. \end{aligned}$$

For $k\in \mathbb {N}$, let $N_k = \max \{ N \in \mathbb {N}^* \mid k \ge k_N \}$, where the maximum of the empty set is defined as 1. Since $(k_N)_{N\in \mathbb {N}^*}$ is strictly increasing, we find that $N_k \rightarrow +\infty$ as $k \rightarrow +\infty$. Then the sequence of couplings

$$\begin{aligned} \pi ^k = \pi ^{k,1/N_k}\in \Pi (\mu ^k,\nu ^k),~k\in \mathbb {N}\end{aligned}$$

is such that ${\tilde{\rho }}(J(\pi ^k),J(\pi ))$ vanishes as *k* goes to $+\infty$, and therefore $J(\pi ^k)$ converges weakly to $J(\pi )$. Moreover, since ${\mathcal {W}}_r$-convergence is equivalent to weak convergence coupled with convergence of the *r*-moments, we have that the *r*-moments of $\mu ^k$ and $\nu ^k$ respectively converge to the *r*-moments of $\mu$ and $\nu$, which implies

$$\begin{aligned}&\int _{X\times {\mathcal {P}}(Y)}\left( {d_X^r(x,x_0)}+{\mathcal {W}}_r^r(p,\delta _{y_0})\right) J(\pi ^k)(dx,dp)\\&\quad =\int _X\left( {d_X^r(x,x_0)}+{\mathcal {W}}_r^r(\pi ^k_x,\delta _{y_0})\right) \mu ^k(dx)\\&\quad =\int _X{d_X^r(x,x_0)}\,\mu ^k(dx)+\int _Yd_Y^r(y,y_0)\nu ^k(dy)\\&\quad \underset{k\rightarrow +\infty }{\longrightarrow }\int _X{d_X^r(x,x_0)}\,\mu (dx)+\int _Yd_Y^r(y,y_0)\,\nu (dy)\\&\qquad =\int _{X\times {\mathcal {P}}(Y)}\left( {d_X^r(x,x_0)}+{\mathcal {W}}_r^r(p,\delta _{y_0})\right) \,J(\pi )(dx,dp). \end{aligned}$$

We deduce that $J(\pi ^k)$ converges to $J(\pi )$ in $\mathcal {W}_r$ as $k\rightarrow +\infty$. According to (1.3), $\pi ^{k,\varepsilon }$ converges to $\pi ^{*,\varepsilon }$ in ${\mathcal {A}}{\mathcal {W}}_r$, which concludes the proof. $\square$

In the proof of Theorem 2.6 we need to be able to confine approximative sequences of couplings to certain sets. The next result provides all necessary tools for this.

### Lemma 3.4

Let $\mu ,\mu ^k\in {\mathcal {M}}_r(X)$, $\nu ,\nu ^k\in {\mathcal {M}}_r(Y)$, $k\in \mathbb {N}$ all with equal masses and $\pi ^k \in \Pi (\mu ^k,\nu ^k)$, $k\in \mathbb {N}$, converge to $\pi \in \Pi (\mu ,\nu )$ in ${\mathcal {A}}{\mathcal {W}}_r$. Let also $A\subset X$ be measurable and $B\supset A$ be open.

(i) There are ${\tilde{\mu }}^k \le \mu ^k|_B$ and $\epsilon _k\ge 0$, $k\in \mathbb {N}$ such that ${\tilde{\mu }}^k(B)=(1-\epsilon _k)\mu (A)$ and ${\tilde{\pi }}^k:={\tilde{\mu }}^k\times \pi ^k_x$ satisfies
  $$\begin{aligned} {\mathcal {A}}{\mathcal {W}}_r({\tilde{\pi }}^k,(1-\epsilon _k)\pi |_{A\times Y})+\epsilon _k\underset{k\rightarrow +\infty }{\longrightarrow }0. \end{aligned}$$
(ii) Let $C\subset {Y}$ be an open set on which $\nu$ is concentrated. There are ${\hat{\mu }}^k \le {\tilde{\mu }}^k$, ${\hat{\nu }}^k\le \nu ^k$, ${\hat{\pi }}^k={\hat{\mu }}^k\times {\hat{\pi }}^k_x\in \Pi ({\hat{\mu }}^k,{\hat{\nu }}^k)$ concentrated on $B\times C$ and $\epsilon '_k\ge 0$, $k\in \mathbb {N}$ such that
  $$\begin{aligned} {\mathcal {A}}{\mathcal {W}}_r^r({\hat{\pi }}^k,(1-\epsilon '_k)\pi \vert _{A\times Y})+\int _X\mathcal W_r^r({\hat{\pi }}^k_x,\pi ^k_x)\,{\hat{\mu }}^k(dx)+\varepsilon '_k\underset{k\rightarrow +\infty }{\longrightarrow }0. \end{aligned}$$

### Proof

To give the reader some guidance we first give an informal description of the strategy of the proof: In order to find $(\tilde{\pi }^k)_{k \in \mathbb {N}}$ and $({\hat{\pi }}^k)_{k \in \mathbb {N}}$, we first pick, for $k\in \mathbb {N}$, optimizers $\chi ^k \in \Pi (\mu ^k,\mu )$ for ${\mathcal {A}}{\mathcal {W}}_r(\pi ^k,\pi )$. Denote by ${\tilde{\pi }}^k$ the composition of the first marginal of $\chi ^k|_{B \times A}$ with the kernel $(\pi ^k_x)_{x \in X}$. By approximation arguments we will then deduce that ${\tilde{\pi }}^k$ has the desired properties. In the last step, we adequately modify ${\tilde{\pi }}^k$ to a coupling ${\hat{\pi }}^k$ with second marginal concentrated on *C*.

Both assertions are trivial if $\mu (A) = 0$ (and also when $A=X$). So assume that $\mu (A) > 0$.

(i) Let $\chi ^k \in \Pi (\mu ^k,\mu )$ be optimal for ${\mathcal {A}}{\mathcal {W}}_r(\pi ^k,\pi )$ and ${\tilde{\mu }}^k$ be the first marginal of $\chi ^k|_{B\times A}$, $k\in \mathbb {N}$. We set ${\tilde{\pi }}^k={\tilde{\mu }}^k\times \pi ^k_x$ and
  3.28 $$\begin{aligned} \varepsilon _k=1-\frac{\chi ^k(B\times A)}{\chi ^k(X\times A)}=1-\frac{{\tilde{\mu }}^k(X)}{\mu (A)}\cdot \end{aligned}$$
  Let us prove that $\varepsilon _k$ goes to 0 as $k\rightarrow \infty$ before checking that the same holds for ${\mathcal {A}}{\mathcal {W}}_r({\tilde{\pi }}^k,(1-\epsilon _k)\pi |_{A\times Y})$.
  Let $\chi = ({\text {id}},{\text {id}})_*\mu$. Since $\chi ^k(dx_1,dx_2)\, \delta _{(x_2,x_2)}(dx_3,dx_4)$ defines a coupling in $\Pi (\chi ^k,\chi )$, we find
  $$\begin{aligned} \mathcal {W}_r^r (\chi ^k,\chi )&\le \int _{X^4} (d_X(x_1,x_3)^r + d_X(x_2,x_4)^r) \, \chi ^k(dx_1,dx_2) \, \delta _{(x_2,x_2)}(dx_3,dx_4) \\&= \int _{X\times X} d_X(x_1,x_2)^r \, \chi ^k(dx_1,dx_2) \le {\mathcal {A}}{\mathcal {W}}_r^r(\pi ^k,\pi ) \rightarrow 0, \ k\rightarrow {+}\infty . \end{aligned}$$
  Further, let $P:{\mathcal {P}}_r(X\times X)\rightarrow {\mathcal {P}}(X\times X)$ be the homeomorphism given by
  $$\begin{aligned} P(\eta )(dx_1,dx_2)=\frac{(1+d_X(x_1,x_0)^r+d_X(x_2,x_0)^r)\,\eta (dx_1,dx_2)}{\int _{X\times X}(1+d_X(x'_1,x_0)^r+d_X(x'_2,x_0)^r)\,\eta (dx'_1,dx'_2)}, \end{aligned}$$
  for $\eta \in {\mathcal {P}}_r(X\times X)$. Recall (1.1), then it is easy to deduce that $P(\eta ') \rightarrow P(\eta )$ weakly if and only if $\eta ' \rightarrow \eta$ in $\mathcal W_r$. In particular, we find that $P(\chi ^k) \rightarrow P(\chi )$ weakly as *k* goes to $+\infty$. Let $f\in \Phi _r(X\times X)$ and
  $$\begin{aligned} \varphi :X\times X :(x_1,x_2) \mapsto \frac{\mathbb {1}_{X\times A}(x_1,x_2) f(x_1,x_2)}{1+d_X(x_1,x_0)^r+d_X(x_2,x_0)^r}. \end{aligned}$$
  Then $\varphi$ is a bounded measurable map which is continuous w.r.t. the first coordinate. As a consequence of [42, Lemma 2.1], we find
  $$\begin{aligned} \int _{X\times X} \varphi (x_1,x_2) \, P(\chi ^k)(dx_1,dx_2) \rightarrow \int _{X\times X} \varphi (x_1,x_2) \, P(\chi )(dx_1,dx_2),\quad k\rightarrow +\infty , \end{aligned}$$
  which amounts to
  $$\begin{aligned}&\int _{X\times X} f(x_1,x_2) \, \chi ^k \vert _{X\times A} (dx_1,dx_2) \rightarrow \int _{X\times X} f(x_1,x_2) \, \chi \vert _{X\times A} (dx_1,dx_2),\quad k\rightarrow +\infty . \end{aligned}$$
  Therefore (1.1) yields ${\mathcal {W}}_r$-convergence of $\chi ^k|_{X\times A}$ to $\chi |_{X\times A}$. By Portmanteau’s theorem, we have
  $$\begin{aligned}&\chi ^k(B\times A)\le {\chi ^k(X\times A)=}\mu (A)=\chi \vert _{X\times A}(B\times B)\\&\quad \quad \quad \quad \quad \quad \quad \quad \quad \le \liminf _{k\rightarrow +\infty }\chi ^k\vert _{X\times A}(B\times B)=\liminf _{k\rightarrow +\infty }\chi ^k(B\times A), \end{aligned}$$
  By the first equality in (3.28), we deduce that $\epsilon _k$, $k\in \mathbb {N}$ is a null sequence of nonnegative real numbers. We now want to show that
  3.29 $$\begin{aligned} {\mathcal {A}}{\mathcal {W}}_r({\tilde{\mu }}^k \times \pi ^k_x, (1- \epsilon _k) \mu \vert _A \times \pi _x) \rightarrow 0. \end{aligned}$$
  On the one hand, denoting by ${\bar{\mu }}^k$ the second marginal of $\chi ^k|_{B\times A}$, we have that
  3.30 $$\begin{aligned} \begin{aligned} {\mathcal {A}}{\mathcal {W}}_r^r({\tilde{\mu }}^k \times \pi ^k_x, {\bar{\mu }}^k \times \pi _x)&\le \int _{X\times X} \left( d_X^r(x,x') + {\mathcal {W}}_r^r (\pi ^k_x,\pi _{x'})\right) \chi ^k \vert _{B\times A} (dx,dx')\\&\le \int _{X\times X} \left( d_X^r(x,x') + {\mathcal {W}}_r^r (\pi ^k_x,\pi _{x'})\right) \chi ^k (dx,dx')\\&= {\mathcal {A}}{\mathcal {W}}_r^r (\pi ^k, \pi ) \rightarrow 0,\quad k\rightarrow +\infty . \end{aligned} \end{aligned}$$
  On the other hand, let
  $$\begin{aligned} {\check{\mu }}^k = (1 - \epsilon _k) \mu \vert _A,\zeta ^k = {\check{\mu }}^k \wedge {\bar{\mu }}^k\quad \text {and} \alpha _k = {\bar{\mu }}^k(X) - \zeta ^k(X) = {\check{\mu }}^k(X) - \zeta ^k(X). \end{aligned}$$
  Let ${\overline{\chi }}^k\in \Pi ({\bar{\mu }}^k-\zeta ^k,{\check{\mu }}^k-\zeta ^k)$ be optimal for ${\mathcal {A}}{\mathcal {W}}_r^r(({\bar{\mu }}^k-\zeta ^k)\times \pi _x,({\check{\mu }}^k-\zeta ^k)\times \pi _x)$. Since $(({\text {id}},{\text {id}})_*\zeta ^k+{\overline{\chi }}^k)$ is a coupling between ${\bar{\mu }}^k$ and ${\check{\mu }}^k$, we find
  $$\begin{aligned}&{\mathcal {A}}{\mathcal {W}}_r({\bar{\mu }}^k \times \pi _x, {\check{\mu }}^k \times \pi _x) \le \int _X\left( d_X^r(x,x')+{\mathcal {W}}_r^r(\pi _x,\pi _{x'})\right) \,{\overline{\chi }}^k(dx,dx') \\&\quad ={\mathcal {A}}{\mathcal {W}}_r^r(({\bar{\mu }}^k-\zeta ^k)\times \pi _x,({\check{\mu }}^k-\zeta ^k)\times \pi _x)\\&\quad \le {\mathcal {A}}{\mathcal {W}}_r(({\bar{\mu }}^k - \zeta ^k) \times \pi _x, \alpha _k \delta _{(x_0,y_0)}) + {\mathcal {A}}{\mathcal {W}}_r(({\check{\mu }}^k - \zeta ^k) \times \pi _x, \alpha _k \delta _{(x_0,y_0)}). \end{aligned}$$
  In the next estimates we use (3.1). Note that the first marginal of $({\bar{\mu }}^k - \zeta ^k) \times \pi _x$ is dominated by $\mu$ whereas its second marginal is dominated by $\nu$. Thus, denoting $\tau ^k(dy)=\int _X\pi _x(dy)\,({\bar{\mu }}^k - \zeta ^k)(dx)$, we find
  $$\begin{aligned}&{\mathcal {A}}{\mathcal {W}}_r^r(({\bar{\mu }}^k - \zeta ^k) \times \pi _x, \alpha _k \delta _{(x_0,y_0)}) = \int _X\left( d_X^r(x,x_0) +{\mathcal {W}}_r^r(\pi _x,\delta _{y_0})\right) \,({\bar{\mu }}^k - \zeta ^k)(dx) \\&\quad =\int _X d_X^r(x,x_0)\,({\bar{\mu }}^k - \zeta ^k)(dx)+\int _Yd_Y^r(y,y_0)\,\tau ^k(dy)\\&\quad \le I_{\alpha _k}^r(\mu ) + I_{\alpha _k}^r(\nu ). \end{aligned}$$
  Similarly, we find
  $$\begin{aligned} {\mathcal {A}}{\mathcal {W}}_r^r(({\check{\mu }}^k - \zeta ^k) \times \pi _x, \alpha _k \delta _{(x_0,y_0)}) \le I_{\alpha _k}^r(\mu ) + I_{\alpha _k}^r(\nu ). \end{aligned}$$
  If we can show that $\alpha _k$ vanishes for $k\rightarrow +\infty$, then we find by Lemma 3.1 (b) that
  3.31 $$\begin{aligned} {\mathcal {A}}{\mathcal {W}}_r({\bar{\mu }}^k \times \pi _x, {\check{\mu }}^k \times \pi _x)\underset{k\rightarrow +\infty }{\longrightarrow }0, \end{aligned}$$
  and the triangle inequality together with (3.30) and (3.31) yield the assertion, (3.29).
  Since ${\check{\mu }}^k,{\bar{\mu }}^k\le \mu \vert _A$, the densities of ${\check{\mu }}^k$ and ${\bar{\mu }}^k$ with respect to $\mu \vert _A$ satisfy $\frac{d{\check{\mu }}^k}{d\mu \vert _A},\frac{d{\bar{\mu }}^k}{d\mu \vert _A} \le 1$. Then we conclude by
  $$\begin{aligned}&\alpha _k = {\bar{\mu }}^k(X) - \zeta ^k(X) \\&\quad = \int _A\left( \frac{d{\bar{\mu }}^k}{d\mu \vert _A}(x)-\frac{d{\check{\mu }}^k}{d\mu \vert _A}(x)\right) ^+\,\mu (dx)\le \int _A\left( 1-\frac{d{\check{\mu }}^k}{d\mu \vert _A}(x)\right) \,\mu (dx)\\&\quad =\mu (A)-{\check{\mu }}^k(A)=\varepsilon _k\mu (A)\underset{k\rightarrow +\infty }{\longrightarrow }0. \end{aligned}$$
(ii) Let ${\tilde{\nu }}^k$ and ${\tilde{\nu }}$ denote the second marginals of ${\tilde{\mu }}^k \times \pi ^k_x$ and $\mu \vert _A \times \pi _x$ respectively. Since $\mu \vert _A\times \pi _x\le \mu \times \pi _x$ with the second marginal $\nu$ of the right-hand side concentrated on *C*, ${\tilde{\nu }}$ is concentrated on *C* and ${\tilde{\nu }}(C)=\mu (A)$. In a similar way, since ${\tilde{\mu }}^k\times \pi ^k_x\le \mu ^k\times \pi ^k_x$, we have ${\tilde{\nu }}^k\le \nu ^k$. In order to modify ${\tilde{\mu }}^k \times \pi ^k_x$ into a coupling with second marginal concentrated on *C*, we consider ${\tilde{\mu }}^k \times \mathring{\pi }^{k}_x$ with $\mathring{\pi }^{k}_x(dz) = \int _{Y} \mathring{\chi }^{k}_y(dz)\,\pi ^k_x(dy)$ where the coupling $\mathring{\chi }^k\in \Pi ({\tilde{\nu }}^k,(1-\epsilon _k){\tilde{\nu }})$ is ${\mathcal {W}}_r$-optimal. To enable comparison of the second marginal with $\nu ^k$ as in the statement, we take advantage of the inequality ${\tilde{\nu }}^k\le \nu ^k$ and introduce ${\tilde{\mu }}^k \times {\hat{\pi }}^{k}_x$ with ${\hat{\pi }}^{k}_x(dt) = \int _Y {\hat{\chi }}^{k}_z(dt)\,\mathring{\pi }^{k}_x(dz)$ where the coupling ${\hat{\chi }}^k\in \Pi ((1-\epsilon _k)\tilde{\nu },(1-\epsilon _k)\frac{{\tilde{\nu }}(C)}{{\tilde{\nu }}^k(C)}\tilde{\nu }^k|_{C})$ is ${\mathcal {W}}_r$-optimal. The second marginal of ${{\hat{\pi }}^k} = {\tilde{\mu }}^k \times {\hat{\pi }}^{k}_x$ is $(1-\epsilon _k)\frac{{\tilde{\nu }}(C)}{{\tilde{\nu }}^k(C)}\tilde{\nu }^k|_{C}$. By the equality ${\tilde{\nu }}(C)=\mu (A)$ and (3.28) for the equality then the definition of ${\tilde{\nu }}^k$ for the inequality, one has
  $$\begin{aligned} (1-\epsilon _k)\frac{{\tilde{\nu }}(C)}{\tilde{\nu }^k(C)}=\frac{{\tilde{\mu }}^k(X)}{{\tilde{\nu }}^k(C)}\ge 1. \end{aligned}$$
  Setting ${\hat{\mu }}^k = \frac{{\tilde{\nu }}^k(C)}{{\tilde{\mu }}^k(X)}\tilde{\mu }^k \le {\tilde{\mu }}^k$ then ensures that the second marginal ${\hat{\nu }}^k= {\tilde{\nu }}^k|_{C}$ of $\hat{\mu }^k\times {\hat{\pi }}^k_x$ is both concentrated on *C* and not greater than $\nu ^k$. Moreover, ${\hat{\nu }}^k(C)={\hat{\nu }}^k(Y)={\hat{\mu }}^k(X)\le {\tilde{\mu }}^k(X)$ with the right-hand side not greater than $\mu (A)$ by (3.28). Hence
  3.32 $$\begin{aligned} \epsilon '_k := 1-\frac{{\tilde{\nu }}^k(C)}{\mu (A)}\in [0,1]\cdot \end{aligned}$$
  Then it remains to show that
  3.33 $$\begin{aligned} {\mathcal {A}}{\mathcal {W}}_r({\hat{\pi }}^k,(1-\epsilon '_k)\pi \vert _{A\times Y})+\int _X\mathcal W_r^r({\hat{\pi }}^k_x,\pi ^k_x)\,{\hat{\mu }}^k(dx)+\epsilon '_k\underset{k\rightarrow +\infty }{\longrightarrow }0. \end{aligned}$$

Since we have

$$\begin{aligned} \pi ^k_x(dy)\,\mathring{\chi }^k_y(dz)\in \Pi (\pi ^k_x,\mathring{\pi }^k_x),\quad \mathring{\pi }^k_x(dz)\,{\hat{\chi }}^k_z(dt)\in \Pi (\mathring{\pi }^k_x,{\hat{\pi }}^k_x),\\ \int _{x\in X}{\hat{\mu }}^k(dx)\,\pi ^k_x(dy)\,\mathring{\chi }^k_y(dz)=\frac{{\tilde{\nu }}^k(C)}{{\tilde{\mu }}^k(X)}\mathring{\chi }^k(dy,dz),\\ \int _{x\in X}{\hat{\mu }}^k(dx)\,\mathring{\pi }^k_x(dz)\,{\hat{\chi }}^k_z(dt)={\frac{{\tilde{\nu }}^k(C)}{{\tilde{\mu }}^k(X)}}\hat{\chi }^k(dz,dt), \end{aligned}$$

we find plugging the expressions (3.28) and (3.32) that

$$\begin{aligned}&{\mathcal {A}}{\mathcal {W}}_r^r({\hat{\mu }}^k\times \pi ^k_x,{\hat{\mu }}^k\times {\hat{\pi }}^k_x)\\&\ \le \int _X{\mathcal {W}}_r^r(\pi ^k_x,{\hat{\pi }}^k_x)\,{\hat{\mu }}^k(dx)\\&\ \le 2^{r-1}\!\!\int _X\left( {\mathcal {W}}_r^r(\pi ^k_x,\mathring{\pi }^k_x)\!{+}\!{\mathcal {W}}_r^r(\mathring{\pi }^k_x,{\hat{\pi }}^k_x)\right) {\hat{\mu }}^k(dx)\\&\ \le 2^{r-1}\!\int _X\left( \int _{Y\times Y}d_Y^r(y,z)\,\pi ^k_x(dy)\,\mathring{\chi }^k_y(dz){+}\int _{Y\times Y}d_Y^r(z,t)\,\mathring{\pi }^k_x(dz)\,{\hat{\chi }}^k_z(dt)\right) \,{\hat{\mu }}^k(dx)\\&\ =2^{r-1}\left( \frac{{\tilde{\nu }}^k(C)}{{\tilde{\mu }}^k(X)}\int _{Y\times Y}d_Y^r(y,z)\,\mathring{\chi }^k(dy,dz)+{\frac{{\tilde{\nu }}^k(C)}{{\tilde{\mu }}^k(X)}}\int _{Y\times Y}d_Y^r(z,t)\,{\hat{\chi }}^k(dz,dt)\right) \\&\ =2^{r-1}\left( \frac{1-\varepsilon '_k}{1-\varepsilon _k}\mathcal W_r^r({\tilde{\nu }}^k,(1-\varepsilon _k){\tilde{\nu }})+\frac{1}{\mu (A)}\mathcal W_r^r({\tilde{\nu }}^k(C){\tilde{\nu }},{\tilde{\nu }}(C){\tilde{\nu }}^k\vert _C)\right) . \end{aligned}$$

To see convergence to 0, note that since ${\mathcal {A}}{\mathcal {W}}_r$ dominates ${\mathcal {W}}_r$, we find by continuity of the projection on the second marginal that (3.29) implies

$$\begin{aligned} {\mathcal {W}}_r({\tilde{\nu }}^k, (1 - \epsilon _k) {\tilde{\nu }}) \rightarrow 0,\quad k\rightarrow +\infty . \end{aligned}$$

Using Portmanteau’s theorem and the fact that $(1-\varepsilon _k)\rightarrow 1$ as *k* goes to $+\infty$, we have for all nonnegative function $f\in \Phi _r(Y)$

$$\begin{aligned} \limsup _{k\rightarrow +\infty }{\tilde{\nu }}^k(\mathbb {1}_Cf)\le \limsup _{k\rightarrow +\infty }{\tilde{\nu }}^k(f)={\tilde{\nu }}(f)={\tilde{\nu }}(\mathbb {1}_Cf)\le \liminf _{k\rightarrow +\infty }{\tilde{\nu }}^k(\mathbb {1}_Cf), \end{aligned}$$

hence

3.34 $$\begin{aligned} {\tilde{\nu }}^k\vert _C(f)\rightarrow {\tilde{\nu }}(f),\quad k\rightarrow +\infty . \end{aligned}$$

Moreover, (3.34) applied with $f=1$ yields ${\tilde{\nu }}^k(C)\rightarrow {\tilde{\nu }}(C)=\mu (A)$ as *k* goes to $+\infty$, hence $\varepsilon '_k$ vanishes as *k* goes to $+\infty$ and

$$\begin{aligned}{\mathcal {W}}_r({\tilde{\nu }}^k(C) {\tilde{\nu }}, {\tilde{\nu }}(C) {\tilde{\nu }}^k\vert _C) \rightarrow 0,\quad k\rightarrow +\infty . \end{aligned}$$

We deduce that

$$\begin{aligned} {\mathcal {A}}{\mathcal {W}}_r^r({\hat{\mu }}^k\times \pi ^k_x,{\hat{\mu }}^k\times {\hat{\pi }}^k_x)\le \int _X\mathcal W_r^r(\pi ^k_x,{\hat{\pi }}^k_x)\,{\hat{\mu }}^k(dx)\rightarrow 0,\quad k\rightarrow +\infty . \end{aligned}$$

On the other hand, by the definition of ${\hat{\mu }}^k$ as $\frac{{\tilde{\nu }}^k(C)}{{\tilde{\mu }}^k(X)}{\tilde{\mu }}^k$, (3.28) and (3.32) we have ${\hat{\mu }}^k=\frac{1-\varepsilon _k'}{1-\varepsilon _k}{\tilde{\mu }}^k$, hence

$$\begin{aligned} {\mathcal {A}}{\mathcal {W}}_r({\hat{\mu }}^k\times \pi ^k_x,(1-\varepsilon '_k)\mu \vert _A\times \pi _x)=\frac{1-\varepsilon '_k}{1-\varepsilon _k}{\mathcal {A}}{\mathcal {W}}_r({\tilde{\mu }}^k\times \pi ^k_x,(1-\varepsilon _k)\mu \vert _A\times \pi _x), \end{aligned}$$

where the right-hand side vanishes as *k* goes to $+\infty$ by the first part. Then (3.33) follows by triangle inequality and the latter convergences, which completes the proof. $\square$

The addition of measures is continuous with respect to the weak and Wasserstein topology. More precisely, we have the estimate

$$\begin{aligned} {\mathcal {W}}_r^r(\mu +\mu ',\nu +\nu ')\le \mathcal W_r^r(\mu ,\nu )+{\mathcal {W}}_r^r(\mu ',\nu ') \end{aligned}$$

for all measures $\mu ,\mu ',\nu ,\nu '\in {\mathcal {P}}_r(X)$ such that $\mu$ and $\nu$, resp. $\mu '$ and $\nu '$ have equal masses.

When considering the adapted weak topology, the next example disproves a comparable statement.

### Example 3.5

Let $X = Y = \mathbb {R}$, and $\pi ^k = \delta _{\left( \frac{1}{k},1\right) }$, $\chi ^k = \delta _{\left( -\frac{1}{k},-1\right) }$, $k\in \mathbb {N}$. Then both sequences are convergent in ${\mathcal {A}}{\mathcal {W}}_1$, but

$$\begin{aligned} {\mathcal {A}}{\mathcal {W}}_1(\pi ^k + \chi ^k, \delta _{(0,1)} + \delta _{(0,-1)}) = \frac{2}{k} + 2 \end{aligned}$$

does not vanish.

However, we show in the next lemma that the addition of measures with respect to the adapted weak topology can still be considered to be continuous in a certain sense if one of the limits has mass significantly smaller than the other.

### Lemma 3.6

Let ${\hat{\mu }},{\hat{\mu }}^k,{\hat{\nu }},{\hat{\nu }}^k\in {\mathcal {M}}_r(Y)$, $k\in \mathbb {N}$ be with equal masses and ${\tilde{\mu }},{\tilde{\mu }}^k,{\tilde{\nu }},{\tilde{\nu }}^k\in {\mathcal {M}}_r(Y)$, $k\in \mathbb {N}$ be with equal masses smaller than $\varepsilon$. Let ${\hat{\pi }}^k\in \Pi ({\hat{\mu }}^k,{\hat{\nu }}^k),{\tilde{\pi }}^k\in \Pi ({\tilde{\mu }}^k,{\tilde{\nu }}^k)$, $k\in \mathbb {N}$, ${\hat{\pi }}\in \Pi ({\hat{\mu }},{\hat{\nu }})$ and ${\tilde{\pi }}\in \Pi ({\tilde{\mu }},{\tilde{\nu }})$. Let $\mu ={\hat{\mu }}+{\tilde{\mu }}$ and $\nu ={\hat{\nu }}+{\tilde{\nu }}$. Then

- We have for all $k\in \mathbb {N}$
  3.35 $$\begin{aligned} \begin{aligned}&{\mathcal {A}}{\mathcal {W}}_r^r({\hat{\pi }}^k+{\tilde{\pi }}^k,{\hat{\pi }}+{\tilde{\pi }})\\&\le {\mathcal {A}}{\mathcal {W}}_r^r({\hat{\pi }}^k,{\hat{\pi }})+2^{r-1}\left( I_\varepsilon ^r({\tilde{\mu }})+I_\varepsilon ^r({\tilde{\mu }}^k)+I_\varepsilon ^r({\tilde{\nu }})+I_\varepsilon ^r({\tilde{\nu }}^k) + 2I_\varepsilon ^r({\hat{\nu }}) + 2I_\varepsilon ^r({\hat{\nu }}^k)\right) \\&\le {\mathcal {A}}{\mathcal {W}}_r^r({\hat{\pi }}^k,{\hat{\pi }})+(2^{r-1})^2\left( {\mathcal {W}}_r^r({\tilde{\mu }}^k,{\tilde{\mu }})+{\mathcal {W}}_r^r({\tilde{\nu }}^k,{\tilde{\nu }})+2{\mathcal {W}}_r^r({\hat{\nu }}^k,{\hat{\nu }})\right) \\&\quad +2^{r-1}(1+2^{r-1})I_\varepsilon ^r(\mu )+3\cdot 2^{r-1}(1+2^{r-1})I_\varepsilon ^r(\nu ), \end{aligned} \end{aligned}$$
  where $I_\varepsilon ^r(\cdot )$ is defined by (3.1).
- If $({\hat{\pi }}^k)_{k\in \mathbb {N}}$ converges to ${\hat{\pi }}$ in ${\mathcal {A}}{\mathcal {W}}_r$ and $(\mu ^k = {\hat{\mu }}^k+{\tilde{\mu }}^k)_{k\in \mathbb {N}}$, resp. $(\nu ^k = {\hat{\nu }}^k+{\tilde{\nu }}^k)_{k\in \mathbb {N}}$, converges to $\mu$, resp. $\nu$, in ${\mathcal {W}}_r$, then
  3.36 $$\begin{aligned} \limsup _{k\rightarrow +\infty }{\mathcal {A}}{\mathcal {W}}_r^r({\hat{\pi }}^k+{\tilde{\pi }}^k,{\hat{\pi }}+{\tilde{\pi }})\le C(I^r_\varepsilon (\mu )+I^r_\varepsilon (\nu )), \end{aligned}$$
  where $C>0$ depends only on *r*.

### Proof

The second inequality of (3.35) is easily deduced from the first one, (3.2) and the fact that $I_\varepsilon ^r({\tilde{\mu }})\le I_\varepsilon ^r(\mu )$, $I_\varepsilon ^r({\tilde{\nu }})\le I_\varepsilon ^r(\nu )$ and $I_\varepsilon ^r({\hat{\nu }})\le I_\varepsilon ^r(\nu )$.

To see (b), assume for a moment that the first inequality of (3.35) holds true and suppose

$$\begin{aligned} {\hat{\pi }}^k \rightarrow {\hat{\pi }}\; \text {in }{\mathcal {A}}{\mathcal {W}}_r,\quad \mu ^k = {\hat{\mu }}^k+{\tilde{\mu }}^k \rightarrow \mu \quad \text {and}\quad \nu ^k = {\hat{\nu }}^k+{\tilde{\nu }}^k\rightarrow \nu \; \text {in }{\mathcal {W}}_r \end{aligned}$$

as $k\rightarrow +\infty$. Using Lemma 3.1 (a) and then (c), we obtain

$$\begin{aligned}&\limsup _{k\rightarrow +\infty }{\mathcal {A}}{\mathcal {W}}_r^r({\hat{\pi }}^k+{\tilde{\pi }}^k,{\hat{\pi }}+{\tilde{\pi }})\\&\quad \le C'\limsup _{k\rightarrow +\infty }\left( I^r_\varepsilon (\mu ^k)+I_\varepsilon ^r(\nu ^k)+I^r_\varepsilon (\mu )+I^r_\varepsilon (\nu )\right) \\&\quad \le C\limsup _{k\rightarrow +\infty }\left( {\mathcal {W}}_r^r(\mu ^k,\mu )+{\mathcal {W}}_r^r(\nu ^k,\nu )+I_\varepsilon ^r(\mu )+I_\varepsilon ^r(\nu )\right) \\&\quad =C(I_\varepsilon ^r(\mu )+I_\varepsilon ^r(\nu )), \end{aligned}$$

where $C,C'>0$ depend only on *r*. Hence (b) is proved.

To conclude the proof, it remains to show the first inequality in (3.35). Let ${\hat{\rho }}^k\in \Pi ({\hat{\mu }}^k,{\hat{\mu }})$ be optimal for ${\mathcal {A}}{\mathcal {W}}_r({\hat{\pi }}^k,{\hat{\pi }})$ and ${\tilde{\rho }}^k\in \Pi ({\tilde{\mu }}^k,{\tilde{\mu }})$ be arbitrary. We write $\rho ^k = {\hat{\rho }}^k + {\tilde{\rho }}^k$. Then

3.37 $$\begin{aligned}&{\mathcal {A}}{\mathcal {W}}_r^r({\hat{\pi }}^k+{\tilde{\pi }}^k, {\hat{\pi }}+{\tilde{\pi }}) \nonumber \\&\quad \le \int _{X\times X}\left( d_X^r(x,x')+\mathcal W_r^r(({\hat{\pi }}^k+{\tilde{\pi }}^k)_x,({\hat{\pi }}+{\tilde{\pi }})_{x'})\right) \,\rho ^k(dx,dx'). \end{aligned}$$

Let ${\hat{p}}=\frac{d{\hat{\mu }}}{d\mu }$ and ${\hat{p}}^k=\frac{d{\hat{\mu }}^k}{d\mu ^k}$. Notice that ${\hat{p}}$ and ${\hat{p}}^k$ take values in [0, 1]. The identities

$$\begin{aligned} ({\hat{\pi }}+{\tilde{\pi }})(dx,dx')=\mu (dx)\,\Big ({\hat{p}}(x)\,{\hat{\pi }}_x(dx')+(1-{\hat{p}}(x))\,{\tilde{\pi }}_x(dx')\Big ),\\ ({\hat{\pi }}^k+{\tilde{\pi }}^k)(dx,dx')=\mu ^k(dx)\,\Big ({\hat{p}}^k(x)\,{\hat{\pi }}^k_x(dx')+(1-{\hat{p}}^k(x))\,{\tilde{\pi }}^k_x(dx')\Big ), \end{aligned}$$

provide representations for the disintegrations of $({\hat{\pi }} + {\tilde{\pi }})$ and $({\hat{\pi }}^k + {\tilde{\pi }}^k)$ respectively for $\mu (dx)$- and $\mu ^k(dx)$-almost every *x*:

$$\begin{aligned} ({\hat{\pi }}+{\tilde{\pi }})_x={\hat{p}}(x)\,{\hat{\pi }}_x+(1-{\hat{p}}(x)){\tilde{\pi }}_x,\quad ({\hat{\pi }}^k+{\tilde{\pi }}^k)_x={\hat{p}}^k(x)\,{\hat{\pi }}^k_x+(1-{\hat{p}}^k(x))\,{\tilde{\pi }}^k_x. \end{aligned}$$

Thus, we have when letting $\alpha ^k_+(x,x') = ({\hat{p}}^k(x) - {\hat{p}}(x'))^+$, $\alpha ^k_-(x,x') = ({\hat{p}}^k(x) - {\hat{p}}(x'))^-$ and $\beta ^k(x,x') = {\hat{p}}^k(x) \wedge {\hat{p}}(x')$ that

3.38 $$\begin{aligned} \begin{aligned}&{\mathcal {W}}_r^r(({\hat{\pi }}^k+{\tilde{\pi }}^k)_x,({\hat{\pi }}+{\tilde{\pi }})_{x'})\\&\quad \le {\mathcal {W}}_r^r(\beta ^k(x,x')\,{\hat{\pi }}^k_x,\beta ^k(x,x')\,{\hat{\pi }}_{x'}) \\&\qquad + {\mathcal {W}}_r^r\Big ( \alpha ^k_+(x,x')\,{\hat{\pi }}^k_x+(1-{\hat{p}}^k(x))\,{\tilde{\pi }}^k_x,\alpha ^k_-(x,x')\,{\hat{\pi }}_{x'}+(1-{\hat{p}}(x'))\,{\tilde{\pi }}_{x'}\Big ) \\&\quad \le \beta ^k(x,x'){\mathcal {W}}_r^r({\hat{\pi }}^k_x,{\hat{\pi }}_{x'}) + 2^{r-1} \left( \alpha _+^k(x,x'){\mathcal {W}}_r^r({\hat{\pi }}^k_x, \delta _{y_0}) + (1 - {\hat{p}}^k(x)) {\mathcal {W}}_r^r({\tilde{\pi }}^k_x,\delta _{y_0}) \right. \\&\qquad \left. + \alpha _-^k(x,x') \mathcal W_r^r({\hat{\pi }}_{x'},\delta _{y_0}) + (1 - {\hat{p}}(x')) \mathcal W_r^r({\tilde{\pi }}_{x'},\delta _{y_0})\right) . \end{aligned} \end{aligned}$$

Since $\beta ^k(x,x') = {\hat{p}}^k(x) \wedge {\hat{p}}(x') \le 1$, we deduce from (3.37), (3.38) and ${\mathcal {A}}{\mathcal {W}}_r$-optimality of ${\hat{\rho }}^k$

3.39 $$\begin{aligned} \begin{aligned} {\mathcal {A}}{\mathcal {W}}_r^r({\hat{\pi }}^k + {\tilde{\pi }}^k, {\hat{\pi }} + {\tilde{\pi }})&\le {\mathcal {A}}{\mathcal {W}}_r^r({\hat{\pi }}^k,{\hat{\pi }}) + \int _{X \times X} d_X(x,x')^r \, {\tilde{\rho }}^k(dx,dx') \\&\quad + 2^{r-1}\int _{X \times X} {\hat{p}}^k(x) {\mathcal {W}}_r^r({\hat{\pi }}^k_x,\delta _{y_0}) \, {\tilde{\rho }}^k(dx,dx') \\&\quad + 2^{r-1}\int _{X \times X} {\hat{p}}(x') {\mathcal {W}}_r^r({\hat{\pi }}_{x'},\delta _{y_0}) \, {\tilde{\rho }}^k(dx,dx') \\&\quad + 2^{r-1} \int _{X \times X} \alpha _+^k(x,x') {\mathcal {W}}_r^r({\hat{\pi }}^k_x, \delta _{y_0}) \, \rho ^k(dx,dx') \\&\quad + 2^{r-1} \int _{X \times X} (1 - {\hat{p}}^k(x)) {\mathcal {W}}_r^r({\tilde{\pi }}^k_{x},\delta _{y_0}) \, \rho ^k(dx,dx') \\&\quad + 2^{r-1} \int _{X \times X} \alpha _-^k(x,x'){\mathcal {W}}_r^r({\hat{\pi }}_{x'}, \delta _{y_0}) \, \rho ^k(dx,dx') \\&\quad +2^{r-1} \int _{X \times X} (1 - {\hat{p}}(x')) {\mathcal {W}}_r^r({\tilde{\pi }}_{x'},\delta _{y_0}) \, \rho ^k(dx,dx'). \end{aligned} \end{aligned}$$

Recall that ${\tilde{\rho }}^k$ has marginals ${\tilde{\mu }}^k$ and ${\tilde{\mu }}$ with total mass smaller than $\epsilon$. By (3.1) we find

3.40 $$\begin{aligned} \int _{X\times X} d_X(x,x')^r \, {\tilde{\rho }}^k(dx,dx') \le 2^{r-1} \left( I_\varepsilon ^r({\tilde{\mu }}^k)+I_\varepsilon ^r({\tilde{\mu }})\right) . \end{aligned}$$

Concerning the marginals of ${\hat{p}}^k(x) \, \rho (dx,dx')$ and ${\hat{p}}(x') \, \rho (dx,dx')$, we find the relations

$$\begin{aligned} {\hat{p}}^k(x) \, {\tilde{\mu }}^k(dx) = (1 - {\hat{p}}^k(x)) \, {\hat{\mu }}^k(dx), \quad {\hat{p}}(x') \, {\tilde{\mu }}(dx') = (1 - {\hat{p}}(x')) \, {\hat{\mu }}(dx').\end{aligned}$$

Again by (3.1), we find since ${\tilde{\rho }}^k \in \Pi ({\tilde{\mu }}^k,{\tilde{\mu }})$, ${\hat{\pi }}^k \in \Pi (\hat{\mu }^k,{\hat{\nu }}^k)$ and ${\hat{\pi }} \in \Pi ({\hat{\mu }},\hat{\nu })$ that

3.41 $$\begin{aligned}&\int _{X \times X} {\hat{p}}^k(x) {\mathcal {W}}_r^r(\hat{\pi }^k_x,\delta _{y_0}) \, {\tilde{\rho }}^k(dx,dx') \nonumber \\&\quad = \int _{X \times X} (1 - {\hat{p}}^k(x)) {\mathcal {W}}_r^r({\hat{\pi }}^k_x,\delta _{y_0}) \, {\hat{\mu }}^k(dx) \le I_\epsilon ^r({\hat{\nu }}^k), \end{aligned}$$

3.42 $$\begin{aligned}&\int _{X \times X} {\hat{p}}(x') {\mathcal {W}}_r^r({\hat{\pi }}_{x'},\delta _{y_0}) \, \tilde{\rho }^k(dx,dx') \nonumber \\&= \int _{X \times X} (1 - {\hat{p}}(x')) {\mathcal {W}}_r^r({\hat{\pi }}_{x'},\delta _{y_0}) \, {\hat{\mu }}(dx') \le I_\epsilon ^r({\hat{\nu }}). \end{aligned}$$

We deduce from (3.39) and (3.40)-(3.42) that it is sufficient to show

3.43 $$\begin{aligned} \int _{X\times X} \alpha _+^k(x,x') {\mathcal {W}}_r^r(\hat{\pi }^k_x,\delta _{y_0}) \, \rho ^k(dx,dx')&\le I_\epsilon ^r({\hat{\nu }}^k), \end{aligned}$$

3.44 $$\begin{aligned} \int _{X\times X} (1 - {\hat{p}}^k(x)) {\mathcal {W}}_r^r(\tilde{\pi }^k_x,\delta _{y_0}) \, \rho ^k(dx,dx')&\le I_\varepsilon ^r({\tilde{\nu }}^k), \end{aligned}$$

3.45 $$\begin{aligned} \int _{X\times X} \alpha _-^k(x,x') {\mathcal {W}}_r^r(\hat{\pi }_{x'},\delta _{y_0}) \, \rho ^k(dx,dx')&\le I_\varepsilon ^r({\hat{\nu }}), \end{aligned}$$

3.46 $$\begin{aligned} \int _{X\times X} (1 - {\hat{p}}(x')) {\mathcal {W}}_r^r({\tilde{\pi }}_{x'}, \delta _{y_0}) \, \rho ^k(dx,dx')&\le I_\varepsilon ^r({\tilde{\nu }}). \end{aligned}$$

To see (3.44) and (3.46), note that

3.47 $$\begin{aligned} (1 - {\hat{p}}^k (x)) \, \mu ^k(dx) = \tilde{\mu }^k(dx)\quad \text {and}\quad (1 - {\hat{p}}(x')) \, \mu (dx') = {\tilde{\mu }}(dx'). \end{aligned}$$

As a consequence, the first marginal of $(1 - {\hat{p}}^k(x))\, \rho ^k(dx,dx')$ is ${\tilde{\mu }}^k$, whereas the second marginal of $(1 - {\hat{p}}(x')) \, \rho ^k(dx,dx')$ coincides with ${\tilde{\mu }}$. Hence, as the mass of ${\tilde{\mu }}^k$ and $\tilde{\mu }$ does not exceed $\epsilon$, we have

$$\begin{aligned}&\int _{X \times X} (1 - {\hat{p}}^k(x)){\mathcal {W}}_r^r({\tilde{\pi }}^k_x,\delta _{y_0}) \, \rho ^k(dx,dx')\\&\quad = \int _X {\mathcal {W}}_r^r({\tilde{\pi }}^k_x,\delta _{y_0}) \, {\tilde{\mu }}^k(dx) = {\mathcal {W}}_r^r({\tilde{\nu }}^k, \delta _{y_0}) = I_\epsilon ^r({\tilde{\nu }}^k), \\&\int _{X \times X} (1 - {\hat{p}}(x')) {\mathcal {W}}_r^r(\tilde{\pi }_{x'},\delta _{y_0}) \, \rho ^k(dx,dx')\\&\quad = \int _X {\mathcal {W}}_r^r({\tilde{\pi }}_{x'},\delta _{y_0}) \, {\tilde{\mu }}(dx')=\mathcal W_r^r({\tilde{\nu }}, \delta _{y_0})= I_{\epsilon }^r(\tilde{\nu }). \end{aligned}$$

Next, we show (3.43) and (3.45). To this end, denoting $\rho ^k(dx,dx')=\mu ^k(dx)\,\rho ^k_x(dx')=\mu (dx')\,\overleftarrow{\rho }^k_{x'}(dx)$, we have

$$\begin{aligned}&\alpha _+^k(x,x') \, \rho ^k(dx,dx') \le {\hat{p}}^k(x) \, \rho ^k(dx,dx') \\&\quad = \frac{d {\hat{\mu }}^k}{d \mu ^k}(x)\, \mu ^k(dx) \, \rho ^k_x(dx') = {\hat{\mu }}^k(dx) \, \rho ^k_x(dx'), \\&\alpha _-^k(x,x') \, \rho ^k(dx,dx') \le {\hat{p}}(x') \, \rho ^k(dx,dx') \\&\quad = \frac{d {\hat{\mu }}}{d \mu }(x') \, \mu (dx') \, \overleftarrow{\rho }^k_{x'}(dx) = {\hat{\mu }}(dx') \, \overleftarrow{\rho }^k_{x'}(dx). \end{aligned}$$

In particular, the first marginal of $\alpha _+^k(x,x') \, \rho ^k(dx,dx')$, denoted here by $\tau ^k$, is dominated by $\hat{\mu }^k$, whereas the second marginal of $\alpha _-^k(x,x') \, \rho ^k(dx,dx')$, denoted here by ${\tau ^k}'$, is dominated by $\hat{\mu }$. Concerning the masses of $\tau ^k$ and ${\tau ^k}'$, remember (3.47), $\alpha _+^k(x,x') \le 1 - {\hat{p}}(x')$ and $\alpha _-^k(x,x') \le 1 - {\hat{p}}^k(x)$, thus,

$$\begin{aligned} \tau ^k(X) = \int _{X \times X} \alpha _+^k(x,x') \, \rho ^k(dx,dx') \le \int _{X} (1 - {\hat{p}}(x')) \, \mu (dx') = {\tilde{\mu }}(X) \le \epsilon , \\ {\tau ^k}'(X) = \int _{X \times X} \alpha _-^k(x,x') \, \rho ^k(dx,dx') \le \int _X (1 - {\hat{p}}^k(x)) \, \mu ^k(dx) = {\tilde{\mu }}^k(X) \le \epsilon . \end{aligned}$$

Using (3.1), we conclude with

$$\begin{aligned} \int _{X \times X} \alpha _+^k(x,x') {\mathcal {W}}_r^r({\hat{\pi }}^k_x, \delta _{y_0}) \, \rho ^k(dx,dx') = \int _X {\mathcal {W}}_r^r({\hat{\pi }}^k_x,\delta _{y_0}) \, \tau (dx) \le I_\epsilon ^r({\hat{\nu }}^k), \\ \int _{X \times X} \alpha _-^k(x,x') {\mathcal {W}}_r^r({\hat{\pi }}_{x'}, \delta _{y_0}) \, \rho ^k(dx,dx') = \int _X {\mathcal {W}}_r^r({\hat{\pi }}_{x'},\delta _{y_0}) \, \tau '(dx') \le I_\epsilon ^r({\hat{\nu }}). \end{aligned}$$

$$\square $$

The addition on ${\mathcal {M}}_r(X\times Y)$ is continuous with respect to the adapted weak topology provided the limits have singular first marginal distributions. We recall that two positive measures $\mu ,\nu$ are called singular if and only if there exists a measurable set $A\subset X$ such that $\mu (A^\complement )=0=\nu (A)$.

### Lemma 3.7

Let $\pi ,\chi \in {\mathcal {M}}_r(X\times Y)$ be such that their respective first marginals are singular. Let $\pi ^k,\chi ^k\in {\mathcal {M}}_r(X\times Y)$, $k\in \mathbb {N}$ converge to $\pi$ and $\chi$ respectively in ${\mathcal {A}}{\mathcal {W}}_r$. Then

$$\begin{aligned} \pi ^k+\chi ^k\underset{k\rightarrow +\infty }{\longrightarrow }\pi +\chi \quad \text {in }{\mathcal {A}}{\mathcal {W}}_r. \end{aligned}$$

### Proof

Let $\mu _1$, $\mu _2$, $\mu _1^k$ and $\mu _2^k$ denote the respective first marginals of $\pi$, $\chi$, $\pi ^k$ and $\chi ^k$. Due to singularity, there is a measurable set $A\subset X$ such that $\mu _1(A^\complement )=0=\mu _2(A)$.

Suppose first that for all $k\in \mathbb {N}$, $\mu ^k_1(A^\complement )=0=\mu ^k_2(A)$. Let $\rho ^k_1\in \Pi (\mu ^k_1,\mu _1)$, resp. $\rho ^k_2\in \Pi (\mu ^k_2,\mu _2)$, be an optimal coupling for ${\mathcal {A}}{\mathcal {W}}_r(\pi ^k,\pi )$, resp. ${\mathcal {A}}{\mathcal {W}}_r(\chi ^k,\chi )$. Since almost surely

$$\begin{aligned} (\pi ^k+\chi ^k)_x=\mathbb {1}_A(x)\,\pi ^k_x+\mathbb {1}_{A^\complement }(x)\chi ^k_x\quad \text {and}\quad (\pi +\chi )_x=\mathbb {1}_A(x)\,\pi _x+\mathbb {1}_{A^\complement }(x)\chi _x, \end{aligned}$$

we have

$$\begin{aligned}&{\mathcal {A}}{\mathcal {W}}_r^r(\pi ^k+\chi ^k,\pi +\chi )\\&\quad \le \int _{X\times X}\left( d^r_X(x,x')+{\mathcal {W}}_r^r((\pi ^k+\chi ^k)_x,(\pi +\chi )_{x'})\right) \,(\rho ^k_1+\rho ^k_2)(dx,dx')\\&\quad =\int _{X\times X}\left( d_X^r(x,x')+{\mathcal {W}}_r^r(\pi ^k_x,\pi _{x'})\right) \,\rho ^k_1(dx,dx')\\&\qquad +\int _{X\times X}\left( d_X^r(x,x')+{\mathcal {W}}_r^r(\chi ^k_x,\chi _{x'})\right) \,\rho ^k_2(dx,dx')\\&\quad ={\mathcal {A}}{\mathcal {W}}_r^r(\pi ^k,\pi )+{\mathcal {A}}{\mathcal {W}}_r^r(\chi ^k,\chi )\rightarrow 0,\quad k\rightarrow +\infty . \end{aligned}$$

Let us now go back to the general case. Let $\varepsilon >0$. Since *X* is a Polish space, $\mu _1$ and $\mu _2$ are inner regular, so there exist two compact sets $K_1\subset A$ and $K_2\subset A^\complement$ such that

$$\begin{aligned} \mu _1(K_1^\complement )<\varepsilon \quad \text {and}\quad \mu _2(K_2^\complement )<\varepsilon . \end{aligned}$$

Since *X* is metrizable, it is normal, hence we can separate the closed, disjoint sets $K_1$ and $K_2$ by open, disjoint sets $\tilde{K}_1$ and ${\tilde{K}}_2$ where $K_1 \subset {\tilde{K}}_1$ and $K_2 \subset {\tilde{K}}_2$. Then Lemma 3.4 (i) provides sequences $({\tilde{\mu }}^k_1 \times \pi ^k_x)_{k\in \mathbb {N}}$ and $({\tilde{\mu }}^k_2 \times \chi ^k_x)_{k\in \mathbb {N}}$ with values in ${\mathcal {M}}(X\times Y)$ and null sequences $(\varepsilon _k)_{k\in \mathbb {N}}$ and $(\eta _k)_{k\in \mathbb {N}}$ with values in [0, 1], such that ${\tilde{\mu }}^k_1\le \mu ^k_1\vert _{\tilde{K}_1}$, ${\tilde{\mu }}^k_2\le \mu ^k_2\vert _{{\tilde{K}}_2}$ and, for $k\rightarrow +\infty$,

$$\begin{aligned}&{\mathcal {A}}{\mathcal {W}}_r^r({\tilde{\mu }}^k_1\times \pi ^k_x,(1-\varepsilon _k)\pi \vert _{K_1\times Y})\\&\quad +{\mathcal {A}}{\mathcal {W}}_r^r({\tilde{\mu }}^k_2\times \chi ^k_x,(1-\eta _k)\chi \vert _{K_2\times Y})\rightarrow 0. \end{aligned}$$

To apply Lemma 3.6 (b), let $0 < \epsilon ' \le \epsilon$ be such that $\epsilon ' (\mu _1(K_1) + \mu _2(K_2)) < \epsilon$. Let *k* be sufficiently large such that $\epsilon ^k \wedge \eta ^k < \epsilon '$. We consider the sequences

$$\begin{aligned} {\hat{\pi }}^k= & {} \frac{1 - \epsilon '}{1 - \epsilon ^k} {\tilde{\mu }}^k_1 \times \pi ^k_x + \frac{1 - \epsilon '}{1 - \eta ^k} {\tilde{\mu }}^k_2 \times \chi ^k_x, \quad {\hat{\pi }} = (1 - \epsilon ')\left( \pi \vert _{K_1 \times Y} + \chi \vert _{K_2 \times Y}\right) , \\ {\tilde{\pi }}^k= & {} \pi ^k + \chi ^k - {\hat{\pi }}^k, \quad {\tilde{\pi }} = \pi + \chi - {\hat{\pi }}, \end{aligned}$$

where ${\tilde{\pi }}^k$ is well-defined in ${\mathcal {M}}_r(X \times Y)$ since $\epsilon ^k < \epsilon '$ and $\eta ^k < \epsilon '$. Note that as $k\rightarrow +\infty$,

$$\begin{aligned}&{\mathcal {A}}{\mathcal {W}}_r^r\left( \frac{1 - \epsilon '}{1 - \epsilon ^k} {\tilde{\mu }}^k_1 \times \pi ^k_x, (1 - \epsilon ') \pi \vert _{K_1 \times Y}\right) \\&\qquad \qquad \qquad \qquad \qquad \qquad \quad = \frac{1 - \epsilon '}{1 - \epsilon ^k} {\mathcal {A}}{\mathcal {W}}_r^r\left( {\tilde{\mu }}^k_1 \times \pi ^k_x, (1 - \epsilon ^k) \pi \vert _{K_1 \times Y}\right) \rightarrow 0, \\&{\mathcal {A}}{\mathcal {W}}_r^r\left( \frac{1 - \epsilon '}{1 - \eta ^k} {\tilde{\mu }}^k_2 \times \chi ^k_x, (1 - \epsilon ') \chi \vert _{K_2 \times Y}\right) \\&\qquad \qquad \qquad \qquad \qquad \qquad \quad = \frac{1 - \epsilon '}{1 - \eta ^k} {\mathcal {A}}{\mathcal {W}}_r^r\left( {\tilde{\mu }}^k_2 \times \chi ^k_x, (1 - \eta ^k) \chi \vert _{K_2 \times Y}\right) \rightarrow 0. \end{aligned}$$

Since the first marginal distributions of ${\tilde{\mu }}^k_1\times \pi ^k_x$ and $(1-\varepsilon _k)\pi \vert _{K_1\times Y}$, resp. ${\tilde{\mu }}^k_2\times \chi ^k_x$ and $(1-\eta _k)\chi \vert _{K_2\times Y}$, are concentrated on ${\tilde{K}}_1$, resp. ${\tilde{K}}_2$, and since ${\tilde{K}}_1$ and ${\tilde{K}}_2$ are disjoint, we have according to the preceding part that

$$\begin{aligned} {\mathcal {A}}{\mathcal {W}}_r^r({\hat{\pi }}^k,{\hat{\pi }})\rightarrow 0,\quad k\rightarrow +\infty . \end{aligned}$$

Due to ${\mathcal {A}}{\mathcal {W}}_r$-convergence of $(\pi ^k)_{k\in \mathbb {N}}$ and $(\chi ^k)_{k\in \mathbb {N}}$, we obtain $\mathcal W_r$-convergence of the marginals of $\pi ^k + \chi ^k$ to the marginals of $\pi + \chi$. Furthermore, we have

$$\begin{aligned}{\tilde{\pi }}^k(X \times Y) = {\tilde{\pi }}(X \times Y) \le \mu _1(K_1^c) + \mu _2(K_2^c) + \epsilon ' (\mu _1(K_1) + \mu _2(K_2)) < 3 \epsilon . \end{aligned}$$

Then (3.36) yields

$$\begin{aligned} \limsup _{k\rightarrow +\infty }{\mathcal {A}}{\mathcal {W}}_r^r(\pi ^k+\chi ^k,\pi +\chi )&= \limsup _{k\rightarrow +\infty }{\mathcal {A}}{\mathcal {W}}_r^r(\hat{\pi }^k+{\tilde{\pi }}^k,{\hat{\pi }}+{\tilde{\pi }}) \\&\le C\left( I^r_{3\varepsilon }(\mu _1+\mu _2)+I^r_{3\varepsilon }(\nu _1+\nu _2)\right) , \end{aligned}$$

where $\nu _1$ and $\nu _2$ denote the respective second marginals of $\pi$ and $\chi$, and the constant *C* only depends on *r*. Therefore, the right-hand side vanishes as $\epsilon \rightarrow 0$ according to Lemma 3.1 (b), which concludes the proof. $\square$

## Auxiliary results on the convex order in dimension one

We recall that the convex order on ${\mathcal {M}}_1(\mathbb {R})$ is defined by

$$\begin{aligned} \mu \le _c \nu \iff \quad \forall f:\mathbb {R}\rightarrow \mathbb {R}\text { convex},\quad \mu (f) \le \nu (f). \end{aligned}$$

The following assertions can be found for instance be found in [35, Section 2]: for all $(m_0,m_1)\in \mathbb {R}_+^*\times \mathbb {R}$, there is a one-to-one correspondence between finite positive measures $\mu \in {\mathcal {M}}_1(\mathbb {R})$ with mass $m_0$ such that $\int _\mathbb {R}y\,\mu (dy)=m_1$ and the set of functions $u :\mathbb {R}\rightarrow \mathbb {R}^+$ which satisfy

(i) *u* is convex;
(ii) $u(y) - m_0|y-m_1|$ goes to 0 as |*y*| tends to $+\infty$.

Any function which satisfies (i) and (ii) is then called a potential function. As noted above, the potential function of $\mu$ is denoted by

$$\begin{aligned} u_\mu (y) = \int _\mathbb {R}|y-x|\,\mu (dx). \end{aligned}$$

Potential functions can of course also be considered in greater generality than on the real line, but this is not relevant for our purposes.

A sequence $(\mu ^k)_{k\in \mathbb {N}}$ of finite positive measures with equal masses on the line converges in $\mathcal {W}_1$ to $\mu$ if and only if the sequence of potential functions $(u_{\mu ^k})_{k\in \mathbb {N}}$ converges pointwise to $u_\mu$. In that case, since for all $y\in \mathbb {R}$ the map $x\mapsto \vert y-x\vert$ is Lipschitz continuous with constant 1, we have by Kantorovich and Rubinstein’s duality theorem that

$$\begin{aligned} \sup _{y\in \mathbb {R}}\vert u_{\mu ^k}(y)-u_\mu (y)\vert \le \mathcal W_1(\mu ^k,\mu )\rightarrow 0,\quad k\rightarrow +\infty , \end{aligned}$$

hence we even have uniform convergence on $\mathbb {R}$ of potential functions.

For all $m_1\in \mathbb {R}$, the set of all finite positive measures on the real line with mean $m_1$ is a lattice [40, Proposition 1.6], and even a complete lattice [41] for the convex order. Then all $\mu ,\nu \in {\mathcal {M}}_1(\mathbb {R})$ with mean $m_1$ have a supremum, denoted $\mu \vee _c\nu$, and an infimum, denoted $\mu \wedge _c\nu$, with respect to the convex order. In that context it is convenient to work with potential functions since they provide simple characterisations of those bounds:

$$\begin{aligned} \mu \vee _{c}\nu \text { is defined as the measure with potential function } u_\mu \vee u_\nu ,\\ \mu \wedge _{c} \nu \text { is defined as the measure with potential function } {\text {co}}(u_\mu \wedge u_\nu ), \end{aligned}$$

where ${\text {co}}$ is the convex hull.

### Lemma 4.1

Let $(\mu ^k)_{k\in \mathbb {N}}$, $(\nu ^k)_{k\in \mathbb {N}}$ be two sequences of ${\mathcal {M}}_1(\mathbb {R})$ converging respectively to $\mu$ and $\nu$ in ${\mathcal {W}}_1$. Suppose that there exists $(m_0,m_1)\in \mathbb {R}_+^*\times \mathbb {R}$ such that $\mu ^k(\mathbb {R})=\nu ^k(\mathbb {R})=m_0$ and $\int _\mathbb {R}x\, \mu ^k(dx)=\int _\mathbb {R}y\, \nu ^k(dy)=m_1$ for all $k \in \mathbb {N}$. Then

$$\begin{aligned} \lim _{k\rightarrow +\infty } {\mathcal {W}}_1(\mu ^k \vee _c \nu ^k, \mu \vee _c \nu ) = 0\quad \text {and}\quad \lim _{k\rightarrow +\infty } {\mathcal {W}}_1(\mu ^k\wedge _c \nu ^k, \mu \wedge _c \nu ) = 0. \end{aligned}$$

### Proof

Convergence in ${\mathcal {W}}_1$ is equivalent to pointwise convergence of the potential functions. Thus, the convergence of $\mu ^k\vee _c \nu ^k$ to $\mu \vee _c\nu$ in ${\mathcal {W}}_1$ is a consequence of the pointwise convergence of $u_{\mu ^k\vee _c \nu ^k}=u_{\mu ^k}\vee u_{\nu ^k}$ to $u_\mu \vee u_\nu =u_{\mu \vee _c\nu }$.

To show convergence of $\mu ^k\wedge _c \nu ^k$ to $\mu \wedge _c\nu$ in ${\mathcal {W}}_1$, it is sufficient to show for all $x \in \mathbb {R}$

4.1 $$\begin{aligned} u_{\mu ^k\wedge _c \nu ^k}(x)={\text {co}}(u_{\mu ^k}\wedge u_{\nu ^k})(x)\rightarrow {\text {co}}(u_\mu \wedge u_\nu )(x)=u_{\mu \wedge _c \nu }(x),\quad k\rightarrow +\infty . \end{aligned}$$

Since $u_{\mu ^k}$ and $u_{\nu ^k}$ converge uniformly on $\mathbb {R}$ to $u_\mu$ and $u_\nu$ respectively, we have uniform convergence of $u_{\mu ^k}\wedge u_{\nu ^k}$ to $u_\mu \wedge u_\nu$. Let $\varepsilon >0$ and $k_0\in \mathbb {N}$ be such that for all $k\ge k_0$,

$$\begin{aligned} \sup _{x\in \mathbb {R}}\vert (u_{\mu ^k}\wedge u_{\nu ^k})(x)-(u_\mu \wedge u_\nu )(x)\vert \le \varepsilon . \end{aligned}$$

For all $k\ge k_0$, we find

$$\begin{aligned} {\text {co}}(u_{\mu }\wedge u_{\nu })-\varepsilon \le (u_{\mu }\wedge u_{\nu }) - \epsilon \le u_{\mu ^k}\wedge u_{\nu ^k},\\ {\text {co}}(u_{\mu ^k}\wedge u_{\nu ^k})-\varepsilon \le (u_{\mu ^k}\wedge u_{\nu ^k}) - \epsilon \le u_\mu \wedge u_\nu . \end{aligned}$$

Thus, as the convex hull is the supremum over all dominated, convex functions, this yields

$$\begin{aligned} {\text {co}}(u_\mu \wedge u_\nu )-\varepsilon \le {\text {co}}(u_{\mu ^k}\wedge u_{\nu ^k})\le {\text {co}}(u_\mu \wedge u_\nu )+\varepsilon , \end{aligned}$$

which establishes (4.1) and completes the proof. $\square$

We now provide the proof of Proposition 2.5 which is the key argument to see that it is enough to prove our main result, namely Theorem 2.6, for irreducible pairs of marginals.

### Proof of Proposition 2.5

To construct the desired decomposition, pick for all $k\in \mathbb {N}$ a coupling $\pi ^k \in \Pi _M(\mu ^k,\nu ^k)$. Let $l_n$ and $r_n$ denote the left and right boundary of the open interval $\{u_{\mu _n}<u_{\nu _n}\}$ on which $\mu _n$ is concentrated, and set

$$\begin{aligned} \mu ^k_n(dx) = \int _{u = F_\mu (l_n)}^{F_\mu (r_n-)} \delta _{F_{\mu ^k}^{-1}(u)}(dx) \, du, \quad \nu _n^k(dy)=\int _{u=F_\mu (l_n)}^{F_\mu (r_n-)}\pi ^k_{F_{\mu ^k}^{-1}(u)}(dy) \, du.\end{aligned}$$

These are the respective marginals of ${\tilde{\pi }}^{k,n}$ on $\mathbb {R}^2$ given by

4.2 $$\begin{aligned} {\tilde{\pi }}^{k,n}(dx,dy)=\int _{u=F_\mu (l_n)}^{F_\mu (r_n-)}\delta _{F_{\mu ^k}^{-1}(u)}(dx)\,\pi ^k_{F_{\mu ^k}^{-1}(u)}(dy) \, du. \end{aligned}$$

Since $\pi ^k$ is a martingale coupling, we have $\mu ^k_n\le _c\nu ^k_n$. Finally define

$$\begin{aligned} J = [0,1] \backslash \bigcup _{n\in N}(F_\mu (l_n),F_\mu (r_n-)), \end{aligned}$$

and set

$$\begin{aligned} \eta ^k(dx) = \int _{u \in J} \delta _{F_{\mu ^k}^{-1}(u)}(dx) \, du,\quad \upsilon ^k(dy) = \int _{u \in J} \pi ^k_{F^{-1}_{\mu ^k}(u)}(dy) \, du. \end{aligned}$$

These are the respective marginals of ${\tilde{\pi }}^k$ defined by

$$\begin{aligned} {\tilde{\pi }}^k(dx,dy) = \int _{u \in J} \delta _{F_{\mu ^k}^{-1}(u)}(dx)\, \pi ^k_{F_{\mu ^k}^{-1}(u)}(dy) \, du, \end{aligned}$$

which is again a martingale coupling with marginals $(\eta ^k,\upsilon ^k)$, thus, $\eta ^k\le _c\upsilon ^k$.

Using inverse transform sampling for the second equality, we find

$$\begin{aligned} \left( {\tilde{\pi }}^k + \sum _{n\in N} {\tilde{\pi }}^{k,n}\right) (dx,dy)&= \int _{u=0}^{1} \delta _{F_{\mu ^k}^{-1}(u)}(dx)\,\pi ^k_{F_{\mu ^k}^{-1}(u)}(dy)\, du \\&= \int _{x^k\in \mathbb {R}}\delta _{x^k}(dx)\, \pi ^k_{x^k}(dy) \, \mu ^k (dx^k)\\&=\mu ^k(dx)\,\pi ^k_x(dy)=\pi ^k(dx,dy). \end{aligned}$$

Concerning the marginals, we deduce

$$\begin{aligned} \eta ^k + \sum _{n\in N} \mu ^k_n = \mu ^k\quad \text {and}\quad \upsilon ^k + \sum _{n\in N} \nu ^k_n = \nu ^k. \end{aligned}$$

For all $(\tau ,u,l,r)\in \mathcal P_1(\mathbb {R})\times (0,1)\times \mathbb {R}\times \mathbb {R}$, we have by (2.3):

4.3 $$\begin{aligned} F_\tau (l)<u<F_\tau (r-)\implies l<F_\tau ^{-1}(u)<r\implies F_\tau (l)<u\le F_\tau (r-). \end{aligned}$$

Since $\mu _n(dx)=\mathbb {1}_{(l_n,r_n)}(x)\,\mu (dx)$, using (4.3) for the second equality we find

$$\begin{aligned} \mu _n(dx)=\int _{x'\in (l_n,r_n)}\delta _{x'}(dx)\,\mu (dx)=\int _{u = F_\mu (l_n)}^{F_\mu (r_n-)} \delta _{F_{\mu }^{-1}(u)}(dx) \, du. \end{aligned}$$

We deduce that

$$\begin{aligned}&\eta (dx)=\left( \mu -\sum _{n\in N}\mu _n\right) (dx)\\&\quad =\int _{u=0}^1\delta _{F_\mu ^{-1}(u)}(dx)\,du-\sum _{n\in N}\int _{u = F_\mu (l_n)}^{F_\mu (r_n-)} \delta _{F_{\mu }^{-1}(u)}(dx) \, du \\&\quad = \int _{u \in J} \delta _{F_{\mu }^{-1}(u)}(dx) \, du. \end{aligned}$$

Since the monotone rearrangement yields an optimal coupling, we have

$$\begin{aligned} {\mathcal {W}}_1(\eta ^k,\eta ) + \sum _{n\in N} \mathcal W_1(\mu ^k_n,\mu _n)= \int _0^1 | F_{\mu ^k}^{-1}(u) - F_\mu ^{-1}(u) | \, du = {\mathcal {W}}_1(\mu ^k,\mu ), \end{aligned}$$

hence

$$\begin{aligned} \lim _{k\rightarrow +\infty } {\mathcal {W}}_1(\eta ^k,\eta ) = 0 = \lim _{k\rightarrow +\infty } {\mathcal {W}}_1 (\mu ^k_n,\mu _n), \quad \forall n \in N. \end{aligned}$$

Since the marginals of $\pi ^k$ converge weakly, the sequences $(\mu ^k)_{k\in \mathbb {N}}$ and $(\nu ^k)_{k\in \mathbb {N}}$ are tight, and so is $(\pi ^k)_{k\in \mathbb {N}}$. For $n \in N$, ${\tilde{\pi }}^{k,n}$ is dominated by $\pi ^k$, hence $({\tilde{\pi }}^{k,n})_{k\in \mathbb {N}}$ is tight and therefore relatively compact. Moreover, by ${\mathcal {W}}_1$-convergence of $(\mu ^k)_{k\in \mathbb {N}}$ and $(\nu ^k)_{k\in \mathbb {N}}$, the sequences $\left( \int _\mathbb {R}\vert x\vert \,\mu ^k(dx)\right) _{k\in \mathbb {N}}$ and $\left( \int _\mathbb {R}\vert y\vert \,\nu ^k(dy)\right) _{k\in \mathbb {N}}$ converge and are in particular bounded. Hence the sequences $\left( \int _\mathbb {R}\vert x\vert \,\mu ^k_n(dx)\right) _{k\in \mathbb {N}}$ and $\left( \int _\mathbb {R}\vert y\vert \,\nu ^k_n(dy)\right) _{k\in \mathbb {N}}$ are bounded as well and admit convergent subsequences. Since the ${\mathcal {W}}_1$-convergence is equivalent to the weak convergence plus convergence of the first moments, we deduce that the sequence $({\tilde{\pi }}^{k,n})_{k \in \mathbb {N}}$ is relatively compact in ${\mathcal {W}}_1$. Since $(\pi ^k)_{k\in \mathbb {N}}$ is tight, from any subsequence we can extract a further subsequence denoted by $(\pi ^{k_j})_{j\in \mathbb {N}}$ which converges weakly to some $\pi \in \Pi _M(\mu ,\nu )$. There are subsequences $({\tilde{\pi }}^{k_j,n})_{j \in \mathbb {N}}$ converging in ${\mathcal {W}}_1$ to a measure ${\tilde{\pi }}_n$. Moreover $\tilde{\pi }_n \le \pi$ with $\pi \in \Pi _M(\mu ,\nu )$ denoting the weak limit of a subsequence of the tight sequence $(\pi ^{k_j})_{j \in \mathbb {N}}$. The first marginal of ${\tilde{\pi }}_n$ coincides with $\mu _n$ due to the continuity of the projection, thus,

$$\begin{aligned} \tilde{\pi }_n \le \pi \vert _{{(l_n,r_n)} \times \mathbb {R}} ={:} \pi _n. \end{aligned}$$

As ${\tilde{\pi }}_n(\mathbb {R}\times \mathbb {R}) = \mu _n({(l_n,r_n)}) = \pi _n(\mathbb {R}\times \mathbb {R})$, there must hold equality, i.e., ${\tilde{\pi }}_n = \pi _n$ and $\int _{x\in \mathbb {R}}{\tilde{\pi }}_n(dx,dy)=\nu _n(dy)$. By continuity of the projection, we deduce that $\lim _{j\rightarrow \infty }\mathcal W_1(\nu ^{k_j}_n,\nu _n)=0$ and, since the limit does not depend on the subsequence, $(\nu ^k_n)_{k\in \mathbb {N}}$ converges in ${\mathcal {W}}_1$ to $\nu _n$. Analogously, we find that $(\upsilon ^k)_{k \in \mathbb {N}}$ converges to $\eta$. $\square$

The next two lemmas explore the influence of certain scaling and restrictions of measure on condition that the transformed measures are in convex order.

### Lemma 4.2

Let $r\ge 1$ and $\mu \in {\mathcal {M}}_r(\mathbb {R}^d)$ be a finite positive measure. Let $m_1=\int _\mathbb {R}x\,\mu (dx)$ and $\mu ^\alpha$, $\alpha \in \mathbb {R}_+$ be the image of $\mu$ by $y \mapsto \alpha (y-m_1) + m_1$. Then for all $\alpha ,\beta \in \mathbb {R}_+$,

4.4 $$\begin{aligned} \mathcal W_r(\mu ^\alpha ,\mu ^\beta )=\vert \beta -\alpha \vert \left( \int _{\mathbb {R}^d}\vert x-m_1\vert ^r\,\mu (dx)\right) ^{\frac{1}{r}}{=\vert \beta -\alpha \vert \mathcal W_r(\mu ^0,\mu ^1)}. \end{aligned}$$

Moreover, $(\mu ^\alpha )_{\alpha \in \mathbb {R}^+}$ constitutes a peacock, i.e., $\alpha \le \beta \in \mathbb {R}_+$ implies $\mu ^\alpha \le _c \mu ^\beta$.

### Proof

Let $\alpha \le \beta \in \mathbb {R}_+$. By the triangle inequality we obtain

$$\begin{aligned} \left( \int _{\mathbb {R}^d} \vert x-m_1 \vert ^r \, \mu ^\beta (dx) \right) ^{\frac{1}{r}}&= {\mathcal {W}}_r(\delta _{m_1},\mu ^\beta ) \le {\mathcal {W}}_r(\delta _{m_1},\mu ^\alpha ) + {\mathcal {W}}_r(\mu ^\alpha ,\mu ^\beta ) \\&= \left( \int _{\mathbb {R}^d} \vert x - m_1 \vert ^r \, \mu ^\alpha (dx) \right) ^{\frac{1}{r}} + {\mathcal {W}}_r(\mu ^\alpha ,\mu ^\beta ). \end{aligned}$$

Thus,

$$\begin{aligned} {\mathcal {W}}_r(\mu ^\alpha ,\mu ^\beta )&\ge \left( \int _{\mathbb {R}^d} \vert x - m_1 \vert ^r \, \mu ^\beta (dx) \right) ^{\frac{1}{r}} - \left( \int _{\mathbb {R}^d} \vert x - m_1 \vert ^r \, \mu ^\alpha (dx) \right) ^{\frac{1}{r}} \\&= ( \beta - \alpha ) \left( \int _{\mathbb {R}^d} \vert x - m_1 \vert ^r \, \mu (dx) \right) ^{\frac{1}{r}}. \end{aligned}$$

Since the image of $\mu$ under $x\mapsto (\alpha (x-m_1)+m_1,\beta (y-m_1)+m_1)$ is a coupling between $\mu ^\alpha$ and $\mu ^\beta$, we also have the reverse inequality

$$\begin{aligned} {\mathcal {W}}_r (\mu ^\alpha ,\mu ^\beta ) \le (\beta -\alpha ) \left( \int _{\mathbb {R}^d} \vert x - m_1 \vert ^r \, \mu (dx) \right) ^{\frac{1}{r}}, \end{aligned}$$

which proves (4.4).

To see that $(\mu ^\alpha )_{\alpha \in \mathbb {R}^+}$ is a peacock, we fix again $\alpha \le \beta \in \mathbb {R}_+$ and a convex function *f* on $\mathbb {R}^d$. By convexity, we have

$$\begin{aligned} \mu ^\alpha (f)&= \int _{\mathbb {R}^d} f(\alpha (x-m_1)+m_1) \, \mu (dx) \\&\le \int _{\mathbb {R}^d}\left( \frac{\alpha }{\beta } f(\beta (x-m_1)+m_1) + \left( 1 - \frac{\alpha }{\beta }\right) f(m_1)\right) \, \mu (dx) \le \mu ^\beta (f). \end{aligned}$$

$$\square $$

### Lemma 4.3

For all $p\in {\mathcal {P}}_1(\mathbb {R})$ with barycentre $m_1\in \mathbb {R}$ and $R\ge 0$, let $p^R$ be defined by

$$\begin{aligned} p^R=p\wedge _c\left( \frac{R-m_1}{2R}\,\delta _{-R}+\frac{R+m_1}{2R}\,\delta _R\right) \quad \text {if}\quad R\ge \vert m_1\vert , \end{aligned}$$

and $p^R=\delta _{m_1}$ otherwise. Then

- For all $R>0$, $p^R\le _c p$, and if $R\ge \vert m_1\vert$, then $p^R$ is concentrated on $[-R,R]$.
- We have
  $$\begin{aligned} {\mathcal {W}}_1(p^R,p)\underset{R\rightarrow +\infty }{\longrightarrow }0. \end{aligned}$$

### Proof

Let $p\in {\mathcal {P}}_1(\mathbb {R})$ be with barycentre $m_1\in \mathbb {R}$. For all $R\ge \vert m_1\vert$, let $\eta ^R=\frac{R-m_1}{2R}\,\delta _{-R}+\frac{R+m_1}{2R}\,\delta _R$, so that $p^R=p\wedge _c\eta ^R$. If $R<\vert m_1\vert$ then $p^R=\delta _{m_1}$ so we clearly have $p^R\le _c p$. Else, $p^R\le _c p$ still holds by definition of the convex infimum. Moreover, since $\eta ^R$ is concentrated on $[-R,R]$, so is $p^R$ by domination in the convex order, hence (a) is proved.

To show (b), it suffices to verify pointwise convergence of the corresponding potential functions, i.e., for all $y\in \mathbb {R}$,

4.5 $$\begin{aligned} u_{p\wedge \eta ^R}(y)={\text {co}}(u_p\wedge u_{\eta ^R})(y)\rightarrow u_p(y),\quad R\rightarrow +\infty . \end{aligned}$$

Let $\varepsilon >0$. Since $u_p(y)-\vert y-m_1\vert$ vanishes as $\vert y\vert \rightarrow +\infty$, there exists $M>0$ such that

$$\begin{aligned} \forall y\in \mathbb {R},\quad \vert y\vert > M\implies u_p(y)\le \vert y-m_1\vert +\varepsilon . \end{aligned}$$

Let $R_0=\vert m_1\vert +\sup _{{x}\in [-M,M]}u_p({x})$ and $R\ge R_0$. The map $u_{\eta ^R}$ is a piecewise affine function which changes slope at $-R$ and *R* and such that $u_{\eta ^R}(y)\rightarrow +\infty$ as $\vert y\vert \rightarrow +\infty$. It therefore attains its minimum either at $-R$ where it is equal to $R+m_1$ or at *R* where it is equal to $R-m_1$, and this minimum is equal to $R-\vert m_1\vert$. We deduce that for all $y\in \mathbb {R}$, $u_{\eta ^R}(y)\ge R-\vert m_1\vert$. Moreover, $\delta _{m_1}\le _c {\eta ^R}$, hence we also have $u_{\eta ^R}(y)\ge \vert y-m_1\vert$ for all $y\in \mathbb {R}$. Let $y\in \mathbb {R}$. If $\vert y\vert \le M$, then

$$\begin{aligned} u_p(y)\le \sup _{{x \in }[-M,M]}u_p{(x)}=R_0-\vert m_1\vert \le R-|m_1|\le u_{\eta ^R}(y). \end{aligned}$$

If, on the other hand, $\vert y\vert > M$ , then

$$\begin{aligned} u_p(y)\le \vert y-m_1\vert +\varepsilon \le u_{\eta ^R}(y)+\varepsilon . \end{aligned}$$

We deduce that for all $y\in \mathbb {R}$ and $R\ge R_0$, $u_p(y)-\varepsilon \le (u_p\wedge u_{\eta ^R})(y)$. Thus, as the convex hull is the supremum over all dominated, convex functions, this yields

$$\begin{aligned} u_p-\varepsilon \le {\text {co}}(u_p\wedge u_{\eta ^R})\le u_p, \end{aligned}$$

which proves (4.5) and completes the proof. $\square$

## Proof of the main theorem

We consider the setting of Theorem 2.6. Before entering its technical proof, we argue that it is sufficient to consider the case $r=1$ and that we can assume w.l.o.g. that $(\mu ,\nu )$ is irreducible.

When considering a sequence of couplings $(\pi ^k)_{k \in \mathbb {N}}$ which converges in ${\mathcal {A}}{\mathcal {W}}_1$ to $\pi \in \Pi (\mu ,\nu )$, whose sequence of marginal distributions $(\mu ^k,\nu ^k)_{k\in \mathbb {N}}$ is converging in ${\mathcal {W}}_r$, one can deduce ${\mathcal {A}}{\mathcal {W}}_r$-convergence for the sequence of couplings. This is due to (1.3), and $\mathcal W_r$-convergence being equivalent to weak convergence plus convergence of the *r*-moments. To see the latter, we find, when equipping $X \times {\mathcal {P}}_r(Y)$ with the product metric $((x,p),(x',p'))\mapsto (d_X^r(x,x') + {\mathcal {W}}_r^r(p,p'))^{1/r}$,

5.1 $$\begin{aligned} \begin{aligned} \int _{X \times {\mathcal {P}}_r(Y)}\left( d_X^r(x,x_0) + \mathcal W_r^r(p,\delta _{y_0})\right) \, J(\pi ^k)(dx,dp) = \mathcal W_r^r(\mu ^k,\delta _{x_0}) + {\mathcal {W}}_r^r(\nu ^k,\delta _{y_0}) \\ \underset{k\rightarrow +\infty }{\longrightarrow } \mathcal W_r^r(\mu ,\delta _{x_0}) + {\mathcal {W}}_r^r(\nu ,\delta _{y_0}) = \int _{X \times {\mathcal {P}}_r(Y)}\left( d_X^r(x,x_0) + \mathcal W_r^r(p,\delta _{y_0}) \right) \, J(\pi )(dx,dp). \end{aligned} \end{aligned}$$

A direct consequence is the following lemma, according to which proving Theorem 2.6 for $r=1$ is sufficient.

### Lemma 5.1

In the setting of Theorem 2.6, assume that there exists a sequence of martingale couplings $\pi ^k\in \Pi _M(\mu ^k,\nu ^k)$, $k\in \mathbb {N}$ converging to $\pi$ in ${\mathcal {A}}{\mathcal {W}}_1$. Then this sequence also converges to $\pi$ in ${\mathcal {A}}{\mathcal {W}}_r$.

Next, Proposition 2.5 is the key ingredient to show that it is enough to prove Theorem 2.6 when $(\mu ,\nu )$ is irreducible.

### Lemma 5.2

If the conclusion of Theorem 2.6 holds for $r=1$ and for any irreducible pair of marginals $(\mu ,\nu )$, then it holds for $r=1$ and for any pair $(\mu ,\nu )$ in the convex order.

### Proof

In the setting of Theorem 2.6, fix $\pi \in \Pi _M(\mu ,\nu )$. Denote by $(\mu _n,\nu _n)_{n\in N}$ the decomposition of $(\mu ,\nu )$ into irreducible components with

$$\begin{aligned} \mu =\eta + \sum _{n\in N} \mu _n,\quad \nu =\eta +\sum _{n\in N} \nu _n. \end{aligned}$$

By Proposition 2.5, we can find sub-probability measures $(\eta ^k,\upsilon ^k)_{k\in \mathbb {N}}$, $(\mu ^k_n)_{(k,n)\in \mathbb {N}\times N}$, $(\nu ^k_n)_{(k,n)\in \mathbb {N}\times N}$ such that

$$\begin{aligned}&\eta ^k \le _c \upsilon ^k,\quad \mu ^k_n \le _c \nu ^k_n\quad \forall (k,n)\in \mathbb {N}\times N,\\&\eta ^k \rightarrow \eta ,\quad \upsilon ^k \rightarrow \eta ,\quad \mu ^k_n \rightarrow \mu _n,\quad \nu ^k_n\rightarrow \nu _n\quad \text {in }{\mathcal {W}}_1,\quad k\rightarrow +\infty . \end{aligned}$$

For $k\in \mathbb {N}$, let $\chi ^k\in \Pi _M(\eta ^k,\upsilon ^k)$ be a martingale coupling between $\eta ^k$ and $\upsilon ^k$. Since the marginals both converge to $\eta$ in ${\mathcal {W}}_1$, $(\chi ^k)_{k\in \mathbb {N}}$ is tight and any accumulation point with respect to the weak topology belongs to $\Pi _M(\eta ,\eta )$. Since $\chi :=({\text {id}},{\text {id}})_*\eta$ is the only martingale coupling between $\eta$ and itself, $(\chi ^k)_{k\in \mathbb {N}}$ converges weakly to $\chi$ as *k* goes to $+\infty$ and even in ${\mathcal {W}}_1$ according to (5.1). We can show that this convergence also holds in ${\mathcal {A}}{\mathcal {W}}_1$.

Indeed, according to Proposition 2.1, there exists a sequence ${\tilde{\chi }}^k\in \Pi (\eta ^k,\upsilon ^k)$, $k\in \mathbb {N}$, converging to $\chi$ in ${\mathcal {A}}{\mathcal {W}}_1$. Then

$$\begin{aligned}&{\mathcal {A}}{\mathcal {W}}_1 (\chi ^k, {\tilde{\chi }}^k) \le \int _\mathbb {R}{\mathcal {W}}_1 (\chi ^k_x, {\tilde{\chi }}^k_x) \, \eta ^k(dx) \le \int _\mathbb {R}\left( {\mathcal {W}}_1 (\chi ^k_x, \delta _x) + {\mathcal {W}}_1 (\delta _x, {\tilde{\chi }}^k_x )\right) \, \eta ^k(dx)\\&\qquad =\int _\mathbb {R}\int _\mathbb {R}\vert x' - x \vert \, ( \chi ^k_x + {\tilde{\chi }}^k_x ) ( dx' ) \, \eta ^k ( dx ) = \int _\mathbb {R}\vert x' - x \vert \, ( \chi ^k + {\tilde{\chi }}^k ) ( dx, dx' ). \end{aligned}$$

Since $(x,x') \mapsto \vert x'-x \vert \in \Phi _1(\mathbb {R}^2)$ and $\chi ^k$ and ${\tilde{\chi }}^k$ converge to $\chi$ in ${\mathcal {W}}_1$, we deduce, using (1.1), that

$$\begin{aligned} \int _\mathbb {R}\vert x'-x\vert \,(\chi ^k+{\tilde{\chi }}^k)(dx,dx')\rightarrow 2\int _\mathbb {R}\vert x'-x\vert \,\chi (dx,dx')=0,\quad k\rightarrow +\infty , \end{aligned}$$

hence,

$$\begin{aligned} {\mathcal {A}}{\mathcal {W}}_1 (\chi ^k, \chi ) \le {\mathcal {A}}{\mathcal {W}}_1 (\chi ^k, {\tilde{\chi }}^k) + {\mathcal {A}}{\mathcal {W}}_1 ( {\tilde{\chi }}^k, \chi ) \rightarrow 0,\quad k\rightarrow +\infty . \end{aligned}$$

By assumption, we can find for any $n\in N$ a sequence $(\pi ^{k,n})_{k\in \mathbb {N}}$ of martingale couplings between $\mu ^k_n$ and $\nu ^k_n$, $k\in \mathbb {N}$, which converges in ${\mathcal {A}}{\mathcal {W}}_1$ to $\pi _n$ as *k* goes to $+\infty$, where $\pi _n$ denotes $\pi$ restricted to the *n*-th irreducible component given by (2.8). By Lemma 3.7, we have for all $p\in N$ that

$$\begin{aligned} \chi ^k + \sum _{n\in N,\ n\le p}\pi ^{k,n} \rightarrow \chi + \sum _{n\in N,\ n\le p} \pi _n\quad \text {in }{\mathcal {A}}{\mathcal {W}}_1,\quad k\rightarrow +\infty . \end{aligned}$$

Moreover, the respective marginals of $\chi ^k+\sum _{n\in N}\pi ^{k,n}$, namely $\mu ^k$ and $\nu ^k$, converge in ${\mathcal {W}}_1$ to the respective marginals of $\chi +\sum _{n\in N}\pi _n$, namely $\mu$ and $\nu$. Therefore, according to Lemma 3.6 (b), there exists a constant $C>0$ such that

$$\begin{aligned} \limsup _k{\mathcal {A}}{\mathcal {W}}_1\left( \chi ^k + \sum _{n\in N} \pi ^{k,n}, \chi + \sum _{n\in N} \pi _n \right) \le C\left( I_{\varepsilon _p}^1(\mu )+I_{\varepsilon _p}^1(\nu )\right) , \end{aligned}$$

where $\varepsilon _p=\sum _{n\in N,n>p}\mu _n(\mathbb {R})$ where by convention the sum over an empty set is 0. Clearly, $(\epsilon _p)_{p\in N}$ tends to 0, thus Lemma 3.1 (b) reveals that the right-hand side vanishes as *p* goes to $\sup N$. This proves that $\pi ^k = \chi ^k + \sum _{n \in N} \pi ^{k,n} \in \Pi _M(\mu ^k,\nu ^k)$ converges in ${\mathcal {A}}{\mathcal {W}}_1$ to $\pi = \chi + \sum _{n \in N} \pi ^k \in \Pi _M(\mu ,\nu )$. $\square$

### Proof of Theorem 2.6

*Step 1.* Due to Lemma 5.1 and Lemma 5.2, we may suppose w.l.o.g. that $r=1$ and $(\mu ,\nu )$ is irreducible with component $I = (\ell ,\rho )$, $\ell \in \mathbb {R}\cup \{-\infty \}$, $\rho \in \mathbb {R}\cup \{ +\infty \}$. Next, we define auxiliary martingale couplings close to $\pi$ which will be easier to approximate in the limit. We define them with the same first marginal distribution whereby the second marginal distribution is smaller with respect to the convex order. These auxiliary couplings will satisfy two key properties: first, their second marginal distribution must be concentrated on a compact subset of *I* when the first marginal distribution is itself concentrated on a certain compact subset *K* of *I*. Second, it is essential that their second marginal distribution has positive mass on some two compact subsets of *I* on both sides of *K*.

Fix $\epsilon \in (0,\frac{1}{2})$. Choose a compact subset $K=[a,b]$ of *I* with

5.2 $$\begin{aligned} \mu (K^\complement )<\varepsilon . \end{aligned}$$

Instead of directly approximating $\pi$, we initially consider the martingale coupling $\pi ^{R,\alpha }$ whose definition is given below. For any $R>0$, let $(\pi ^R_x)_{x\in \mathbb {R}}$ be the probability kernel obtained by virtue of Lemma 4.3. By Lemma 4.3 (b) we have for all $x \in \mathbb {R}$ that $\pi ^R_x\le _c\pi _x$. Therefore,

$$\begin{aligned} {\mathcal {W}}_1 ( \pi ^R_x, \pi _x ) \le 2 \int _\mathbb {R}\vert y \vert \, \pi _x (dy), \end{aligned}$$

where the right-hand side is a $\mu$-integrable function of *x*. By Lemma 4.3 (b) we find $\pi ^R_x \rightarrow \pi _x$ in ${\mathcal {W}}_1$ as $R \rightarrow +\infty$. Let $\pi ^R:=\mu \times \pi ^R_x$, then dominated convergence yields

$$\begin{aligned} {\mathcal {A}}{\mathcal {W}}_1(\pi ^R,\pi )\le \int _\mathbb {R}\mathcal {W}_1 (\pi _x^R, \pi _x) \, \mu (dx) \rightarrow 0,\quad R\rightarrow +\infty . \end{aligned}$$

Denote by $\nu ^R$ the second marginal of $\pi ^R$. Consequently, $\nu ^R$ converges to $\nu$ for the ${\mathcal {W}}_1$-distance and $\nu ^R \le _c \nu$ for all $R > 0$. Let ${\tilde{a}}$ and ${\tilde{b}}$ be real numbers such that ${\tilde{a}}\in (\ell ,a)$ and ${\tilde{b}}\in (b,\rho )$, for instance

$$\begin{aligned} {\tilde{a}} = \frac{\ell +a}{2} \vee (a-1) \text { and } {\tilde{b}} = (b + 1) \wedge \frac{b+\rho }{2}. \end{aligned}$$

Since $(\mu ,\nu )$ is irreducible on *I*, according to Remark 2.4, $\nu$ assigns positive mass to any neighbourhood in ${\overline{I}}$ of the endpoints $\ell$ and $\rho$ of *I*. From now on, we use the notational convention that for all $c\in \mathbb {R}\cup \{\pm \infty \}$,

$$\begin{aligned}{}[-\infty ,c)=\{x\in \mathbb {R}\mid x<c\},\quad (c,+\infty ]=\{x\in \mathbb {R}\mid c<x\}\quad \text {and}\quad [-\infty ,+\infty ]=\mathbb {R}. \end{aligned}$$

In particular, $${\overline{I}}=[\ell ,\rho ]\subset \mathbb {R}$$.

Then $[\ell ,{\tilde{a}})$ and $({\tilde{b}}, \rho ]$ are relatively open on ${\overline{I}}$ with $\nu ^R({\overline{I}})=1=\nu ({\overline{I}})$, so Portmanteau’s theorem yields

$$\begin{aligned} \liminf _{R \rightarrow +\infty } \nu ^R( [\ell ,{\tilde{a}}) ) \ge \nu ( [\ell ,{\tilde{a}}) )> 0,\quad \liminf _{R \rightarrow + \infty } \nu ^R( ({\tilde{b}},\rho ] ) \ge \nu ( ({\tilde{b}},\rho ] ) > 0. \end{aligned}$$

Thus, we deduce that we can choose $R>0$ such that

5.3 $$\begin{aligned}&R\ge |a|\vee |b|,\ \quad \int _\mathbb {R}\mathcal {W}_1 (\pi _x^R, \pi _x) \, \mu (dx)<\varepsilon ,\quad \nu ^R([\ell ,\tilde{a}))>0,\quad \text {and} \nonumber \\&\quad \nu ^R(({\tilde{b}},\rho ])>0. \end{aligned}$$

Let $\pi ^{R,\alpha }_x$ be the image of $\pi ^R_x$ by $y \mapsto \alpha (y - x) + x$ when $\alpha \in (0,1)$. Then $\pi ^{R,\alpha }:=\mu \times \pi ^{R,\alpha }_x$ satisfies by Lemma 4.2

$$\begin{aligned}&{\mathcal {A}}{\mathcal {W}}_1(\varepsilon \pi +(1-\varepsilon )\pi ^{R,\alpha },\pi )\\&\quad \le \int _\mathbb {R}{\mathcal {W}}_1(\varepsilon \pi _x+(1-\varepsilon )\pi ^{R,\alpha }_x,\pi _x)\,\mu (dx)\\&\quad \le (1-\varepsilon )\int _\mathbb {R}{\mathcal {W}}_1(\pi ^{R,\alpha }_x,\pi _x)\,\mu (dx)\\&\quad \le \int _\mathbb {R}{\mathcal {W}}_1(\pi ^{R,\alpha }_x,\pi ^R_x)\,\mu (dx)+\int _\mathbb {R}{\mathcal {W}}_1(\pi ^R_x,\pi _x)\,\mu (dx)\\&\quad =(1-\alpha )\int _\mathbb {R}\int _\mathbb {R}\vert x-y\vert \,\pi ^{R}_x(dy)\,\mu (dx)+\int _\mathbb {R}{\mathcal {W}}_1(\pi ^R_x,\pi _x)\,\mu (dx)\\&\quad \le (1-\alpha )\left( \int _\mathbb {R}\vert x\vert \,\mu (dx)+\int _\mathbb {R}\vert y\vert \,\nu ^R(dy)\right) +\int _\mathbb {R}\mathcal W_1(\pi ^R_x,\pi _x)\,\mu (dx), \end{aligned}$$

where the right-hand side converges to $\int _\mathbb {R}\mathcal W_1(\pi ^R_x,\pi _x)\,\mu (dx)<\varepsilon$ for $\alpha \rightarrow 1$. Note that $\frac{2R-a-{\tilde{a}}}{2R-2{\tilde{a}}},\frac{b+\tilde{b}+2R}{2{\tilde{b}}+2R}\in (0,1)$, so we can choose $\alpha \in (0,1)$ such that

5.4 $$\begin{aligned} {\mathcal {A}}{\mathcal {W}}_1(\varepsilon \pi +(1-\varepsilon )\pi ^{R,\alpha },\pi )< \epsilon \quad \text {and}\quad \alpha \ge \frac{2R-a-\tilde{a}}{2R-2{\tilde{a}}} \vee \frac{b+{\tilde{b}}+2R}{ 2{\tilde{b}}+2R} . \end{aligned}$$

Let *L* be a compact subset of *I* such that the interior $\mathring{L}$ of *L* satisfies

$$\begin{aligned}{}[(-R) \vee (\alpha \ell + (1-\alpha ) a), R \wedge (\alpha \rho + (1-\alpha ) b )] \subset \mathring{L}. \end{aligned}$$

Because $R \ge (-a)\vee b$ and thereby $[a,b] = K \subset [-R,R]$, we have that $\mu \vert _K\times \pi ^R_x$ is concentrated on $K\times ([-R,R] \cap \overline{I})$. Furthermore, for any $(x,y)\in K \times ([-R,R] \cap \overline{I})$, we find $\alpha y + (1-\alpha ) x \in \mathring{L}$. Hence, $\mu \vert _K \times \pi ^{R,\alpha }_x$ is concentrated on $K \times \mathring{L}$.

Denote the second marginal of $\pi ^{R,\alpha }$ by $\nu ^{R,\alpha }$. Since

$$\begin{aligned} (x,y) \in (\ell , R) \times [\ell , {\tilde{a}}) \implies \ell< (1 - \alpha ) x + \alpha y < R - \alpha (R - {\tilde{a}}) \le \frac{a + {\tilde{a}}}{2}, \end{aligned}$$

we have that

$$\begin{aligned}&\nu ^{R,\alpha }\left( \left( \ell ,\frac{a+{\tilde{a}}}{2}\right) \right) \\&\quad =\int _{\mathbb {R}^2}\mathbb {1}_{\left( \ell ,\frac{a+{\tilde{a}}}{2}\right) }(y)\,\pi ^{R,\alpha }(dx,dy) = \int _{\mathbb {R}^2}\mathbb {1}_{\left( \ell ,\frac{a+{\tilde{a}}}{2}\right) }(\alpha y+(1-\alpha )x)\,\pi ^R(dx,dy)\\&\quad \ge \int _{\mathbb {R}^2}\mathbb {1}_{(\ell ,R)\times [\ell ,\tilde{a})}(x,y)\,\pi ^R(dx,dy)=\int _{(\ell ,R)}\pi ^R_x((-\infty ,\tilde{a}))\,\mu (dx). \end{aligned}$$

If $x \in [R,+\infty )$, then $\pi ^R_x = \delta _x$ and since $R\ge {\tilde{a}}$, $\pi ^R_x((-\infty ,{\tilde{a}}))=0$. Added to the fact that $\mu$ is concentrated on *I*, we obtain

$$\begin{aligned} \int _{(\ell ,R)}\pi ^R_x((-\infty ,\tilde{a}))\,\mu (dx)= & {} \int _\mathbb {R}\pi ^R_x((-\infty ,\tilde{a}))\,\mu (dx)\\= & {} \nu ^R((-\infty ,{\tilde{a}}))=\nu ^R([\ell ,{\tilde{a}}))>0. \end{aligned}$$

We deduce that

5.5 $$\begin{aligned} \nu ^{R,\alpha }\left( \left( \ell ,\frac{a+\tilde{a}}{2}\right) \right)>0\text {, and similarly, }\nu ^{R,\alpha }\left( \left( \frac{b+\tilde{b}}{2},\rho \right) \right) >0. \end{aligned}$$

To summarise, we have constructed a martingale coupling $\pi ^{R,\alpha }\in \Pi _M(\mu ,\nu ^{R,\alpha })$ close to $\pi$ with respect to the ${\mathcal {A}}{\mathcal {W}}_1$-distance in view of (5.4), whose restriction $\pi ^{R,\alpha }\vert _{K\times \mathbb {R}}$ is compactly supported on $K\times L$ and concentrated on $K\times \mathring{L}$. Moreover, the second marginal distribution $\nu ^{R,\alpha }$ is dominated by $\nu$ in the convex order and assigns positive mass on both sides of *K* according to (5.5).

*Step 2.* In the next step we construct a sequence of sub-probability martingale couplings supported on a compact cube $J\times J$ ($K\subset J\subset I$) converging to $\pi ^{R,\alpha }|_{K\times \mathbb {R}}$.

Our first goal is to find a sequence $\nu ^{R,\alpha ,k}$, $k\in \mathbb {N}$, such that $\mu ^k \le _c \nu ^{R,\alpha ,k} \le _c \nu ^k$ and

5.6 $$\begin{aligned} {\mathcal {W}}_1(\nu ^{R,\alpha ,k},\nu ^{R,\alpha }) \rightarrow 0,\quad k \rightarrow \infty . \end{aligned}$$

Defining $$\nu ^{R,\alpha ,k}$$ by

$$\begin{aligned} \nu ^{R,\alpha ,k} = \nu ^k \wedge _{c} (\mu ^k \vee _{c} T_k(\nu ^{R,\alpha })), \end{aligned}$$

where $T_k$ denotes the translation by the difference between the common barycentre of $\mu ^k$ and $\nu ^k$ and the common barycentre of $\nu$ and $\nu ^{R,\alpha }$, i.e., $\int _\mathbb {R}y\, \nu ^k(dy) - \int _\mathbb {R}y\, \nu ^{R,\alpha }(dy)$, fulfils these requirements. Indeed

$$\begin{aligned} {\mathcal {W}}_1 ( T_k ( \nu ^{R,\alpha } ), \nu ^{R,\alpha }) = \left| \int _\mathbb {R}y \,\nu ^k(dy) - \int _\mathbb {R}y\, \nu (dy)\right| \le {\mathcal {W}}_1(\nu ^k,\nu ) \rightarrow 0, \end{aligned}$$

as *k* goes to $+\infty$. Then Lemma 4.1 provides $\nu ^{R,\alpha ,k} \rightarrow \nu \wedge _c ( \mu \vee _{c} \nu ^{R,\alpha }) = \nu ^{R,\alpha }$ in ${\mathcal {W}}_1$ as *k* goes to $+\infty$. By Proposition 2.1 we can approximate $\pi ^{R,\alpha }$ with couplings $\pi ^{R,\alpha ,k} \in \Pi (\mu ^k,\nu ^{R,\alpha ,k})$ in ${\mathcal {A}}{\mathcal {W}}_1$. Unfortunately the sequence $\pi ^{R,\alpha ,k}$, $k\in \mathbb {N}$ does not have to consist of solely martingale couplings. Therefore, we have to adjust the barycentres of its disintegrations, $(\pi ^{R,\alpha ,k}_x)$ to obtain martingale kernels and thereby martingale couplings. Due to (5.5) and inner regularity of $\nu ^{R,\alpha }$, we find compact sets

$$\begin{aligned} L_- \subset \left( \ell , \frac{a+{\tilde{a}}}{2}\right) ,\quad L_+\subset \left( \frac{b+{\tilde{b}}}{2},\rho \right) \end{aligned}$$

with $\nu ^{R,\alpha }$-positive measure. Let ${\tilde{\ell }},\tilde{\rho }\in I$, be such that ${\tilde{\ell }}<\inf (L\cup L-)$ and $\sup (L\cup L_+)<{\tilde{\rho }}$. Then define

5.7 $$\begin{aligned} {\tilde{L}}_-=\left( {\tilde{\ell }},\frac{a+\tilde{a}}{2}\right) ,\quad {\tilde{L}}_+=\left( \frac{b+{\tilde{b}}}{2},\tilde{\rho }\right) \quad \text {and}\quad {\tilde{K}}=\left( \frac{3a+\tilde{a}}{4},\frac{3b+{\tilde{b}}}{4}\right) , \end{aligned}$$

so that ${\tilde{L}}_-$, ${\tilde{L}}_+$ and ${\tilde{K}}$ are bounded and open intervals covering respectively $L_-$, $L_+$ and *K* and such that the distance *e* between ${\tilde{L}}_-\cup \tilde{L}_+$ and ${\tilde{K}}$ is positive:

$$\begin{aligned} e=\inf \left\{ \vert x-y\vert \mid (x,y)\in ({\tilde{L}}_-\cup \tilde{L}_+)\times {\tilde{K}}\right\} \ge \frac{a-\tilde{a}}{4}\wedge \frac{{\tilde{b}}-b}{4}>0. \end{aligned}$$

**Fig. 2:**
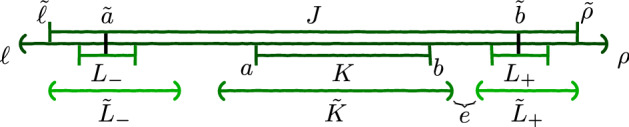
Points and intervals involved in the proof. The boundaries of the closed intervals are vertical bars and those of the open intervals are parentheses

Denoting $J=[{\tilde{\ell }},{\tilde{\rho }}]$, Fig. 2 summarises the construction.

The respective restrictions of $\nu ^{R,\alpha ,k}$ to ${\tilde{L}}_-$ and ${\tilde{L}}_+$ are denoted by $\nu _-^{k}$ and $\nu _+^{k}$, respectively. Since ${\tilde{L}}_-$ and ${\tilde{L}}_+$ are open, Portmanteau’s theorem ensures that eventually (for *k* sufficiently large) $\nu _-^{k}$ and $\nu _+^{k}$ each have more total mass than some constant $\delta > 0$.

By Lemma 3.4 (ii) there are ${\hat{\mu }}^k \le \mu ^k$, ${\hat{\nu }}^k \le \nu ^{R,\alpha ,k}$, ${\hat{\pi }}^{k}={\hat{\mu }}^k\times {\hat{\pi }}^k_x \in \Pi (\hat{\mu }^k, {\hat{\nu }}^k)$ concentrated on ${\tilde{K}} \times \mathring{L}$, and $\epsilon _k \ge 0$ such that

5.8 $$\begin{aligned} {\mathcal {A}}{\mathcal {W}}_1( {\hat{\pi }}^{k}, (1-\epsilon _k) \pi ^{R,\alpha } \vert _{K\times \mathbb {R}} )+\epsilon _k \rightarrow 0,\quad k\rightarrow +\infty . \end{aligned}$$

The following procedure shows that there are for ${\hat{\mu }}^k(dx)$-almost every *x* unique constants $c^k_-(x), c^k_+(x) \in [0,+\infty )$ and $d^k(x) \in [1,+\infty )$ such that

$$\begin{aligned} {\tilde{\pi }}_x^{k} := \frac{{\hat{\pi }}^k_x + c_+^k(x) \nu _+^{k} + c_-^k(x) \nu _-^{k}}{d^k(x)} \in {\mathcal {P}}(\mathbb {R}),\quad \int _\mathbb {R}y\, {\tilde{\pi }}^k_x(dy) = x,\quad c_-^k(x) \wedge c_+^k(x) = 0. \end{aligned}$$

Note that the constraint $c_-^k(x) \wedge c_+^k(x) = 0$ provides

5.9 $$\begin{aligned} \int _\mathbb {R}y\, {\hat{\pi }}^k_x(dy)\le x \implies c_-^k(x) = 0, \quad \int _\mathbb {R}y\,{\hat{\pi }}^k_x(dy) \ge x \implies c_+^k(x) = 0. \end{aligned}$$

We require ${\tilde{\pi }}^k_x$ to be a probability measure with mean *x*, thus,

5.10 $$\begin{aligned}&1 + c_+^k(x) \nu ^k_+(\mathbb {R}) + c_-^k(x) \nu ^k_-(\mathbb {R}) = d^k(x), \end{aligned}$$

5.11 $$\begin{aligned}&\int _\mathbb {R}y \, {\hat{\pi }}^k_x(dy) + c_+^k(x) \int _\mathbb {R}y \, \nu ^k_+(dy) + c_-^k(x) \int _\mathbb {R}y \, \nu _-^k(dy) = x d^k(x). \end{aligned}$$

Combining (5.9) with (5.10) and (5.11) yields

$$\begin{aligned} c_-^k(x)= & {} \frac{\int _\mathbb {R}y \, {\hat{\pi }}^k_x(dy)-x}{ \int _\mathbb {R}(x - y) \, \nu ^k_-(dy)} \vee 0 \in \left[ 0, \frac{\left| x-\int _\mathbb {R}y \, {\hat{\pi }}^k_x(dy)\right| }{ e \nu _-^k(\mathbb {R})} \right] , \\ c_+^k(x)= & {} \frac{x-\int _\mathbb {R}y \, {\hat{\pi }}^k_x(dy)}{\int _\mathbb {R}(y - x) \, \nu _+^k(dy)} \vee 0 \in \left[ 0, \frac{\left| x-\int _\mathbb {R}y \, {\hat{\pi }}^k_x(dy)\right| }{e \nu _+^k(\mathbb {R})}\right] , \\ d^k(x)= & {} 1 + c_-^k(x) \nu _-^k(\mathbb {R}) + c_+^k(x) \nu _+^k(\mathbb {R}) \in \left[ 1, 1 + \frac{\left| x-\int _\mathbb {R}y \, {\hat{\pi }}^k_x(dy)\right| }{e}\right] . \end{aligned}$$

Remember from (5.7) that $L\cup \tilde{L}_-\cup {\tilde{L}}_+\subset [{\tilde{\ell }},{\tilde{\rho }}]\subset I$. Then we obtain for ${\hat{\mu }}^k(dx)$-almost every *x* the estimate

$$\begin{aligned} {\mathcal {W}}_1({\tilde{\pi }}^k_x, {\hat{\pi }}^k_x)&\le {\mathcal {W}}_1\left( \frac{c^k_+(x)\nu ^k_++c_-^k(x) \nu ^k_-}{d^k(x)}, \frac{d^k(x) - 1 }{ d^k(x) } {\hat{\pi }}^k_x \right) \le \frac{d^k(x) - 1}{d^k(x)} |{\tilde{\rho }} - {\tilde{\ell }}|\\&\le \frac{\left| x-\int _\mathbb {R}y \, {\hat{\pi }}^k_x(dy)\right| }{e}\vert {\tilde{\rho }} - \tilde{\ell }\vert . \end{aligned}$$

Hence, the adapted Wasserstein distance between ${\hat{\pi }}^k$ and ${\tilde{\pi }}^k = {\hat{\mu }}^k \times {\tilde{\pi }}^k_x$ satisfies

$$\begin{aligned} {\mathcal {A}}{\mathcal {W}}_1({\tilde{\pi }}^k,{\hat{\pi }}^k)&\le \int _\mathbb {R}{\mathcal {W}}_1({\tilde{\pi }}^k_x,{\hat{\pi }}^k_x) \, {\hat{\mu }}^k(dx)\le \frac{|{\tilde{\rho }} - {\tilde{\ell }}|}{ e }\int _\mathbb {R}\left| x-\int _\mathbb {R}y \, {\hat{\pi }}^k_x(dy)\right| \,{\hat{\mu }}^k(dx)\\&\le \frac{|{\tilde{\rho }} - {\tilde{\ell }}|}{ e } {\mathcal {A}}{\mathcal {W}}_1( {\hat{\pi }}^k, (1 - \epsilon _k) \pi ^{R,\alpha }\vert _{K \times \mathbb {R}}), \end{aligned}$$

where we used Remark 2.2 with exponent $r=1=2^{r-1}$ in the last inequality. The triangle inequality and (5.8) then yield

5.12 $$\begin{aligned} \lim _k {\mathcal {A}}{\mathcal {W}}_1({\tilde{\pi }}^k,(1 - \epsilon _k) \pi ^{R,\alpha }\vert _{K \times \mathbb {R}}) \rightarrow 0,\quad k\rightarrow \infty . \end{aligned}$$

Next we bound the total mass which we require to fix the barycentres. We find that

$$\begin{aligned} \int _\mathbb {R}\frac{c_-^k(x) + c_+^k(x)}{d^k(x)} \, {\hat{\mu }}^k(dx)&\le \frac{1}{e (\nu _-^k(\mathbb {R})\wedge \nu _+^k(\mathbb {R}))}\int _\mathbb {R}\left| x-\int _\mathbb {R}y \, {\hat{\pi }}^k_x(dy)\right| \,{\hat{\mu }}^k(dx)\\&\le \frac{{\mathcal {A}}{\mathcal {W}}_1({\hat{\pi }}^k, (1 - \epsilon _k) \pi ^{R,\alpha }\vert _{K \times \mathbb {R}})}{e (\nu _-^k(\mathbb {R})\wedge \nu _+^k(\mathbb {R}))} \rightarrow 0,\quad k\rightarrow +\infty , \end{aligned}$$

where we used Remark 2.2 again for the last inequality and the fact that $\nu _-^k(\mathbb {R})\wedge \nu _+^k(\mathbb {R})\ge \delta$ for *k* large enough for the limit. Consequently, when ${\tilde{\nu }}^k$ denotes the second marginal of ${\tilde{\pi }}^k$, we have for *k* sufficiently large that

$$\begin{aligned} (1 - 2 \epsilon ) {\tilde{\nu }}^k\le & {} (1 - 2 \epsilon ){\hat{\nu }}^k + (1 - 2 \epsilon )(\nu ^k_- + \nu ^k_+) \int _\mathbb {R}\frac{c_-^k(x) + c_+^k(x)}{d^k(x)} \, {\hat{\mu }}^k(dx) \\\le & {} (1 - \epsilon ) \nu ^{R,\alpha ,k}. \end{aligned}$$

*Step 3.* In this step, we complement the martingale coupling $(1 - 2 \epsilon ) {\tilde{\pi }}^k$ to a martingale coupling with marginals $\mu ^k$ and $\epsilon \nu ^k + (1-\epsilon )\nu ^{R,\alpha ,k}$ for *k* sufficiently large. Recall that ${\tilde{\pi }}^k \in \Pi _M({\hat{\mu }}^k, {\tilde{\nu }}^k)$ and $\pi ^{R,\alpha }\vert _{K \times \mathbb {R}} \in \Pi _M(\mu \vert _K,\check{\nu }^{R,\alpha })$, where ${\check{\nu }}^{R,\alpha }$ is the second marginal distribution of $\pi ^{R,\alpha }\vert _{K\times \mathbb {R}}$, are concentrated on the compact cube $J \times J$ and

$$\begin{aligned} {\mathcal {A}}{\mathcal {W}}_1({\tilde{\pi }}^k, (1 - \epsilon _k) \pi ^{R,\alpha }\vert _{K \times \mathbb {R}}) \rightarrow 0,\quad k\rightarrow +\infty . \end{aligned}$$

Furthermore, since $(1-\epsilon ) \pi ^{R,\alpha } - ( 1 - 2\epsilon ) \pi ^{R,\alpha } \vert _{K\times \mathbb {R}}$ is a martingale coupling with marginals

$$\begin{aligned} (1-\epsilon )\mu - (1 - 2 \epsilon )\mu \vert _K \quad \text {and} \quad (1 - \epsilon ) \nu ^{R,\alpha } - (1 - 2 \epsilon ){\check{\nu }}^{R,\alpha },\end{aligned}$$

we deduce by irreducibility of the pair $(\mu ,\nu )$ on *I* irreducibility of the pair of sub-probability measures

$$\begin{aligned} \varepsilon \mu + (1-\varepsilon )\mu - (1-2\varepsilon ) \mu \vert _K \quad \text {and} \quad \varepsilon \nu + (1-\varepsilon ) \nu ^{R,\alpha } - (1-2\varepsilon ) {\check{\nu }}^{R,\alpha }, \end{aligned}$$

whose potential functions satisfy

$$\begin{aligned} 0 \le u_\mu - u_{(1 - 2\varepsilon ) \mu \vert _K} < u_{\varepsilon \nu + (1-\varepsilon ) \nu ^{R,\alpha }} - u_{(1-2\varepsilon ) \check{\nu }^{R,\alpha }} \quad \text {on }I. \end{aligned}$$

Since those potential functions are continuous, there exists $\tau > 0$ such that they have distance greater $\tau$ on *J*. By uniform convergence of potential functions, for $k\in \mathbb {N}$ sufficiently large we have

$$\begin{aligned} 0 \le u_{\mu ^k} - u_{(1-2\varepsilon ) {\hat{\mu }}^k} + \frac{\tau }{2} \le u_{\varepsilon \nu ^k + (1-\varepsilon ) \nu ^{R,\alpha ,k}} - u_{(1-2\epsilon ) {\tilde{\nu }}^{k}}\quad \text {on }J. \end{aligned}$$

On the complement of *J* we have $u_{(1-2\varepsilon ) {\hat{\mu }}^k} = u_{(1-2\varepsilon ) {\tilde{\nu }}^{k}}$ since both measures are concentrated on *J* and satisfy $(1-2\varepsilon )\int _\mathbb {R}x\, {\hat{\mu }}^k(dx)=(1-2\varepsilon )\int _\mathbb {R}y\,{\tilde{\nu }}^{k}(dy)$. Therefore,

$$\begin{aligned} 0 \le u_{\mu ^k} - u_{(1-2\varepsilon ){\hat{\mu }}^k} \le u_{\varepsilon \nu ^k + (1-\varepsilon ) \nu ^{R,\alpha ,k}} - u_{(1-2\varepsilon ) {\tilde{\nu }}^{k}}\quad \text {on }J^c. \end{aligned}$$

By Strassen’s theorem [52], there exists $\eta ^k\in \Pi _M(\mu ^k - (1-2\varepsilon ) {\hat{\mu }}^k,\varepsilon \nu ^k + (1 - \varepsilon ) \nu ^{R,\alpha ,k} - (1-2\varepsilon ) {\tilde{\nu }}^{k})$. Finally, we write

$$\begin{aligned} {\overline{\pi }}^k = \eta ^k + (1-2\varepsilon ) {\tilde{\pi }}^{k} \in \Pi _M(\mu ^k,\varepsilon \nu ^k + (1-\varepsilon ) \nu ^{R,\alpha ,k}). \end{aligned}$$

*Step 4.* In the last step, we show that the sequence constructed in this way is eventually close to the original martingale coupling $\pi$ in adapted Wasserstein distance.

The marginals of ${\overline{\pi }}^k$ are converging in ${\mathcal {W}}_1$ to $(\mu ,\epsilon \nu + (1 - \epsilon ) \nu ^{R,\alpha })$ as *k* goes to $+\infty$. We have according to (5.12) that

$$\begin{aligned} {\mathcal {A}}{\mathcal {W}}_1\left( (1-2\varepsilon )\frac{1-\varepsilon }{1-\varepsilon _k}{\tilde{\pi }}^k,(1-2\varepsilon )(1-\varepsilon )\pi ^{R,\alpha }\vert _{K \times \mathbb {R}}\right) \rightarrow 0,\quad k \rightarrow \infty .\end{aligned}$$

For *k* large enough so that $\varepsilon _k\le \varepsilon$,

$$\begin{aligned}&{\bar{\pi }}^k(\mathbb {R}^2)-(1-2\varepsilon )\frac{1-\varepsilon }{1-\varepsilon _k}{\tilde{\pi }}^k(\mathbb {R}^2)\\&\quad =\eta ^k(\mathbb {R}^2)+(1-2\varepsilon )\frac{\varepsilon -\varepsilon _k}{1-\varepsilon _k}{\tilde{\pi }}^k (\mathbb {R}^2)\\&\quad =\left( \varepsilon \pi +(1-\varepsilon )\pi ^{R,\alpha }-(1-2\varepsilon )(1-\varepsilon _k)\pi ^{R,\alpha }\vert _{K\times \mathbb {R}}\right) (\mathbb {R}^2)\\&\quad \quad +(1-2\varepsilon )(\varepsilon -\varepsilon _k)\pi ^{R,\alpha }\vert _{K\times \mathbb {R}}(\mathbb {R}^2)\\ {}&\quad =1-(1-2\varepsilon )(1-\varepsilon )\mu (K)\le 4\varepsilon , \end{aligned}$$

where we used $\mu (K) \ge 1-\varepsilon$ for the last inequality. Hence applying Lemma 3.6 (b), with $({\hat{\pi }}^k,{\hat{\pi }},{\tilde{\pi }}^k,{\tilde{\pi }},\varepsilon )$ replaced by

$$\begin{aligned}&\left( (1-2\varepsilon )\frac{1-\varepsilon }{1-\varepsilon _k}{\tilde{\pi }}^k,(1-2\varepsilon )(1-\varepsilon )\pi ^{R,\alpha }\vert _{K \times \mathbb {R}},\right. \\&\qquad \left. \eta ^k+(1-2\varepsilon )\frac{\varepsilon -\varepsilon _k}{1-\varepsilon _k}{\tilde{\pi }}^k,\varepsilon \pi +(1-\varepsilon )\left( \pi ^{R,\alpha }-(1-2\varepsilon )\pi ^{R,\alpha }\vert _{K \times \mathbb {R}}\right) , 4\varepsilon \right) , \end{aligned}$$

we obtain

$$\begin{aligned} \limsup _k{\mathcal {A}}{\mathcal {W}}_1({\overline{\pi }}^k,\varepsilon \pi +(1-\varepsilon )\pi ^{R,\alpha })\le C(I_{4\varepsilon }(\mu )+I_{4\varepsilon }(\varepsilon \nu +(1-\varepsilon )\nu ^{R,\alpha })), \end{aligned}$$

with *C* given by Lemma 3.6 (b) and depending only on the exponent $r = 1$. Since $\nu ^{R,\alpha }\le _c\nu$, then $\varepsilon \nu +(1-\varepsilon )\nu ^{R,\alpha }\le _c\nu$, so using Lemma 3.1 (e), the triangle inequality and (5.4), we get

$$\begin{aligned}&\limsup _k{\mathcal {A}}{\mathcal {W}}_1({\overline{\pi }}^k,\pi )\\&\quad \le \limsup _k\left( {\mathcal {A}}{\mathcal {W}}_1({\overline{\pi }}^k,\varepsilon \pi +(1-\varepsilon )\pi ^{R,\alpha })+{\mathcal {A}}{\mathcal {W}}_1(\varepsilon \pi +(1-\varepsilon )\pi ^{R,\alpha },\pi )\right) \\&\quad \le C(I_{4\varepsilon }(\mu )+I_{4\varepsilon }(\nu ))+\varepsilon . \end{aligned}$$

Since the right-hand side only depends on $\epsilon$ and vanishes as $\epsilon$ goes to 0, we can reason like in the proof of Proposition 2.3 (from (3.27)) to find a null sequence $({\tilde{\epsilon }}_k)_{k \in \mathbb {N}}$, two sequences $(R_k)_{k\in \mathbb {N}}$, $(\alpha _k)_{k\in \mathbb {N}}$ with values respectively in $\mathbb {R}_+^*$ and (0, 1), and martingale couplings

$$\begin{aligned} \mathring{\pi }^k\in \Pi _M(\mu ^k, {\tilde{\epsilon }}_k \nu ^k + (1-{\tilde{\epsilon }}_k) \nu ^{R_k,\alpha _k,k}),\quad k\in \mathbb {N}\end{aligned}$$

such that

5.13 $$\begin{aligned} {\mathcal {A}}{\mathcal {W}}_1(\mathring{\pi }^k,\pi )\rightarrow 0,\quad k\rightarrow +\infty . \end{aligned}$$

In particular, the ${\mathcal {W}}_1$-distance of their second marginal distributions vanishes as *k* goes to $+\infty$, hence the triangle inequality yields, for $k\rightarrow +\infty$,

$$\begin{aligned}&{\mathcal {W}}_1({\tilde{\epsilon }}_k \nu ^k + (1-{\tilde{\epsilon }}_k) \nu ^{R_k,\alpha _k,k},\nu ^k)\\&\quad \le {\mathcal {W}}_1({\tilde{\epsilon }}_k \nu ^k + (1-{\tilde{\epsilon }}_k) \nu ^{R_k,\alpha _k,k},\nu )+\mathcal W_1(\nu ,\nu ^k)\rightarrow 0. \end{aligned}$$

Remember that $\nu ^{R_k,\alpha _k,k}\le _c\nu ^k$, hence $\tilde{\epsilon }_k \nu ^k + (1-{\tilde{\epsilon }}_k) \nu ^{R_k,\alpha _k,k}\le _c\nu ^k$. Then by [37, Theorem 2.12], there exist martingale couplings $M^k \in \Pi _M({\tilde{\epsilon }}_k \nu ^k + (1 - \tilde{\epsilon }_k)\nu ^{R_k,\alpha _k,k},\nu ^k)$, $k\in \mathbb {N}$ such that, for $k\rightarrow +\infty$,

5.14 $$\begin{aligned} \int _{\mathbb {R}\times \mathbb {R}} |x-y|\,M^k(dx,dy) \le 2\mathcal {W}_1(\tilde{\epsilon }_k \nu ^k + (1 - \tilde{\epsilon }_k)\nu ^{R_k,\alpha _k,k},\nu ^k)\rightarrow 0. \end{aligned}$$

Let then

$$\begin{aligned} \pi ^k(dx,dy)=\mu ^k(dx)\int _{z\in \mathbb {R}}M^k_z(dy)\,\mathring{\pi }^k_x(dz)\in \Pi _M(\mu ^k,\nu ^k). \end{aligned}$$

Using the fact that for $\mu ^k(dx)$-almost every *x*, $\mathring{\pi }^k_x(dz)\,M^k_z(dy)\in \Pi (\mathring{\pi }^k_x,\pi ^k_x)$, we get

$$\begin{aligned} {\mathcal {A}}{\mathcal {W}}_1(\pi ^k,\mathring{\pi }^k)&{\le }\int _\mathbb {R}{\mathcal {W}}_1(\pi ^k_x,\mathring{\pi }^k_x)\, \mu ^k(dx){\le }\int _{\mathbb {R}\times \mathbb {R}\times \mathbb {R}}\vert z-y\vert \,\mu ^k(dx)\,M^k_z(dy)\,\,\mathring{\pi }^k_x(dz)\\&=\int _{\mathbb {R}\times \mathbb {R}}\vert z-y\vert \,M^k(dy,dz), \end{aligned}$$

where the right-hand side vanishes by (5.14) as *k* goes to $+\infty$. Then (5.13) and the triangle inequality yield

$$\begin{aligned} {\mathcal {A}}{\mathcal {W}}_1(\pi ^k,\pi )\le {\mathcal {A}}{\mathcal {W}}_1(\pi ^k,\mathring{\pi }^k)+{\mathcal {A}}{\mathcal {W}}_1(\mathring{\pi }^k,\pi )\rightarrow 0,\quad k\rightarrow +\infty , \end{aligned}$$

which concludes the proof. $\square$

## Footnotes

1 Note that this can be made precise in terms of hemicontinuity. We also refer to [45].
